# Design, Synthesis, and Biological Evaluation of *N*-Acyl-Hydrazone-Linked Quinazolinone Derivatives with Antioxidant, Antimicrobial, and Anticancer Potential

**DOI:** 10.3390/ph19010057

**Published:** 2025-12-26

**Authors:** Maria Coandă, Constantin Drăghici, Lucia Pintilie, Erzsébet-Eleonóra Kapronczai, Cornel Chiriță, Ioana-Cristina Marinaș, Robert-Viorel Ancuceanu, Irina Zarafu, Petre Ioniță, Denisa-Ioana Crăciun, Ariana Hudiță, Bianca Gălățeanu, Carmen Limban, Diana Camelia Nuță

**Affiliations:** 1 Department of Pharmaceutical Chemistry, Faculty of Pharmacy, “Carol Davila” University of Medicine and Pharmacy, Traian Vuia No. 6, 020956 Bucharest, Romania; maria.coanda@drd.umfcd.ro (M.C.); carmen.limban@umfcd.ro (C.L.); diana.nuta@umfcd.ro (D.C.N.); 2 “C. D. Nenitzescu” Institute of Organic and Supramolecular Chemistry, 202B Splaiul Independenței, 060023 Bucharest, Romania; cst_drag@yahoo.com; 3 National Institute for Chemical-Pharmaceutical Research and Development, 112 Vitan Av., 031299 Bucharest, Romania; lucia.pintilie@gmail.com; 4 Department of Chemistry, Supramolecular Organic and Organometallic Chemistry Centre, Faculty of Chemistry and Chemical Engineering, Babeş-Bolyai University, 11 Arany János, 400028 Cluj-Napoca, Romania; erzsebet.denes@ubbcluj.ro; 5 Department of Pharmacology and Clinical Pharmacy, Faculty of Pharmacy, “Carol Davila” University of Medicine and Pharmacy, Traian Vuia No. 6, 020956 Bucharest, Romania; 6 The National Research Institute of the University of Bucharest (ICUB), University of Bucharest, Splaiul Independenței 91-95, 050095 Bucharest, Romania; ioana.cristina.marinas@gmail.com (I.-C.M.); ariana.hudita@bio.unibuc.ro (A.H.); bianca.galateanu@unibuc.ro (B.G.); 7 Department of Pharmaceutical Botany and Cell Biology, Faculty of Pharmacy, “Carol Davila” University of Medicine and Pharmacy, Traian Vuia No. 6, 020956 Bucharest, Romania; robert.ancuceanu@umfcd.ro; 8 Department of Organic Chemistry, Biochemistry and Catalysis, Faculty of Chemistry, University of Bucharest, 4-12 Regina Elisabeta, 030018 Bucharest, Romania; zarafuirina@yahoo.fr (I.Z.); petre.ionita@chimie.unibuc.ro (P.I.); 9 Department of Biochemistry and Molecular Biology, Faculty of Biology, University of Bucharest, Splaiul Independenței 91-95, 050095 Bucharest, Romania; craciun.denisa-ioana@bio.unibuc.ro

**Keywords:** hydrazone, quinazolinone, antimicrobial, anticancer, antioxidant

## Abstract

**Objectives**: Combining two pharmacophores into one molecule with multiple applications presents interest in the field of medicinal chemistry. Quinazolinones are among privileged scaffolds due to their wide biological activities, whereas hydrazones are versatile linkers with pharmacological potential. Thus, this article focused on a green method for the synthesis of new *N*-acyl-hydrazones of 2-(2-methyl-4-oxoquinazolin-3(4*H*)-yl)acetohydrazide and the exploration of their biological potential. **Methods**: The novel *N*-acyl-hydrazones (**1a**–**1f**) were synthesized under microwave irradiation, using various substituted salicylaldehydes and benzaldehydes. The products were characterized by FT-IR, ^1^H-NMR, ^13^C-NMR, and HRMS. Their pharmacological profile was assessed by in silico methods and docking simulations. Biological evaluation included antioxidant, antimicrobial, and cytotoxic activities, as well as preliminary toxicity on *Artemia franciscana*. **Results**: Spectroscopic data indicated *syn-E* and *anti-E* isomers. Compound **1c** showed the highest antioxidant activity. Antimicrobial assays indicated narrow-spectrum activity, with compounds **1a** and **1b** being most effective against *C. albicans* and *S. aureus*. Biofilm inhibition assays revealed that **1a** and **1c** interfered with microbial adhesion, highlighting their potential in combating biofilm-associated infections. Cytotoxicity tests on HT-29 and A431 cancer cell lines showed selective anticancer effects for compounds **1a**–**1d**, with minimal toxicity on normal Vero cells, especially for **1b** and **1d**. Toxicity against *Artemia franciscana* correlated with in vitro cytotoxicity data, revealing low lethality for all *N*-acyl-hydrazones. Docking studies indicate that the antibacterial activity may involve inhibition of *S. aureus* DNA gyrase B, whereas the cytotoxic effects could be mediated by interaction with the EGFR kinase. **Conclusions**: These findings may increase the chances of identifying a lead compound in this class, supporting the further development of selected *N*-acyl-hydrazones and their pharmacological exploration.

## 1. Introduction

Heterocyclic scaffolds represent important structural elements in medicinal chemistry, as they play a role in the design and optimization of bioactive molecules. Among these, quinazolin-2(1*H*)-ones and quinazolin-4(3*H*)-ones, are widely recognized as privileged structural motifs. They have been reported to exhibit a large spectrum of biological activities, including anticancer [1,2,3,4,5,6,7,8,9,10], antiviral [11], antibacterial [12,13], antitubercular [7], antifungal [12,14], antioxidant and analgesic [15] properties. This scaffold is also incorporated into clinically used drugs, such as the second-line anticancer agent, idelalisib, a phosphatidylinositol-4,5-bisphosphate 3-kinase inhibitor [16].

The structural versatility of the quinazolinone core allows for the generation of numerous derivatives with distinct biological profiles, thereby facilitating detailed investigations into structure–activity relationships (SAR) [17]. One such strategy is molecular hybridization, which involves the combination of two or more pharmacophores into a single molecule to increase activity [18,19] or alleviate side effects [20,21]. In this context, research focused on obtaining hybrid molecules that combine two pharmacophoric nuclei, the quinazolinone core and the *ortho*-hydroxy-substituted aromatic moiety, has gained significant relevance. Equally important is the linker connecting these two pharmacophores, as it plays a crucial role in modulating the physicochemical and biological properties of the resulting compounds [21]. Hydrazones, due to their high hydrolytic stability and ease of synthesis, are suitable linkers in this regard [21,22,23].

**Figure 1:**
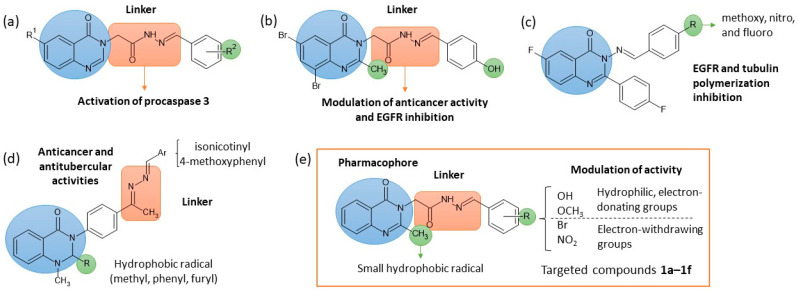
Representative quinazolinone–hydrazone derivatives previously reported in the literature (**a**–**d**) and the proposed molecular model (**e**).

For example, (*E*)-*N*′-benzylidene-2–(4-oxoquinazolin-3(4*H*)-yl)acetohydrazide was identified as a pharmacophore with cytotoxic potential against cancer cell lines, acting as a procaspase activator (Figure 1a) [2,3,4,5]. Substitution on the benzene ring with 2-hydroxy-4-methoxy or 2-hydroxy-3-allyl groups (R^2^) is the most suitable for anticancer activity [2,3,5]. Adding neutral (methyl) or electron-withdrawing groups (halogen or nitro) at position 6 of the quinazolinone ring (R^1^) significantly increases cytotoxicity and procaspase activation [2,3,4,5]. Regarding the mechanism of action, the 2-hydroxy-substituted *N*-acyl-hydrazone scaffold is essential for activating procaspase by chelating the Zn^2+^ ion of the allosteric site [2,3,5].

3-Amino-6,8-dibromo-2-methylquinazolin-4(3*H*)-one was identified as a lead compound, with submicromolar to nanomolar IC_50_ against MCF-7 and MDA-MB-231, inducing both extrinsic and intrinsic apoptosis and downregulating epidermal growth factor receptor (EGFR) expression [1]. The effects of N3-linkers (imine, *N*-acyl-hydrazone, oxadiazole) on anticancer activity and EGFR kinase inhibition of this scaffold was also examined. It was shown that the aliphatic *N*-acyl-hydrazone linker increased potency against MDA-MB-231 cells while reducing activity against HepG2 cells (Figure 1b) [24].

In a similar way, 3-(benzylideneamino)-6-fluoro-2-(4-fluorophenyl)quinazolin-4(3*H*)-ones exhibited cytotoxicity against MCF-7 and MDA-MB-231 cells, exceeding that of geftinib, and inhibited EGFR and tubulin polymerization. The selectivity varied with the substituents on the benzylidene moiety. Thus, methoxy, nitro, and fluoro groups (R) favoured the activity on MDA-MB-231 cells, whereas hydroxy increased the activity on MCF-7 (Figure 1c) [9].

Because cancer patients are particularly vulnerable to opportunistic infections due to their immunocompromised status, the development or identification of oncolytic agents with intrinsic antimicrobial properties could provide dual therapeutic benefit [25]. For example, dual anti-tuberculosis agents and EGFR inhibitors were designed based on 3-(4-{[(arylidene)hydrazono]ethyl}phenyl)-1-methyl-2,3-dihydroquinazolin-4(1*H*)-one scaffold (Figure 1d). The SAR study underscored the importance of the lipophilic substituents (methyl, phenyl) in position 2 of the quinazolinone ring for both anticancer and antitubercular activities. In contrast, polar substituents (furyl) were preferred for EGFR inhibition. The arylidene moiety must contain either isonicotinyl or 4-methoxyphenyl for the compounds to be active [7].

Based on the pharmacological relevance of quinazolinone scaffolds and the potential of the *N*-acyl-hydrazone moiety to enhance biological activity, the present study focused on developing a preliminary series of *N*-acyl-hydrazone-linked quinazolinone derivatives. The following drug design rationale was applied (Figure 1e): (i) retaining (*E*)-*N′*-benzylidene-2–(4-oxoquinazolin-3(4*H*)-yl)acetohydrazide as the structure backbone; (ii) introducing a small lipophilic methyl group in position 2 of the quinazolinone ring to enhance activity; (iii) varying the substituents of the phenyl ring to modulate activity. A hydroxy group is mandatorily present, the 2-hydroxy-substituted *N*-acyl-hydrazone scaffold being essential for activity [2,3,5]. Methoxy and bromo groups were also considered due to their importance for antibacterial, antifungal and cytotoxic properties [3,9,26,27,28,29]. A microwave-assisted synthetic approach was employed to provide an environmentally sustainable and time-efficient method for compound preparation [30].

Biological evaluation included cytotoxicity profiling on cancer and non-tumoral cell lines, antimicrobial testing against Gram-positive and Gram-negative bacteria and fungi, and assessment of antibiofilm activity. Antioxidant capacity was also examined, given the presence of the hydroxyphenylimino moiety [31]. In silico studies were performed to predict pharmacokinetic behavior and drug-likeness, and docking simulations were used to explore potential molecular targets underlying both antimicrobial and anticancer effects. Finally, preliminary in vivo toxicity was assessed using the *Artemia franciscana* model. Collectively, these investigations provide an integrated foundation for defining the pharmacological potential of this series of quinazolinone-based hydrazone derivatives.

## 2. Results

### 2.1. Chemistry and Spectral Data

**Scheme 1:**
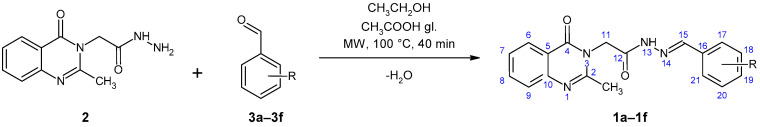
Synthesis of *N*-acyl-hydrazones **1a**–**1f**. Radical R is: 2-OH-5-Br (**a**); 2-OH-3,5-Br_2_ (**b**); 2-OH-3-OCH_3_ (**c**); 2-OH-4-OCH_3_ (**d**); 2-OH-5-OCH_3_ (**e**); 3-OCH_3_-4-OH-5-NO_2_ (**f**). The atom numbering for NMR attributions is presented in blue.

In this research, *N*-acyl-hydrazones **1a**–**1f** were obtained starting from 2-(2-methyl-4-oxoquinazolin-3(4*H*)-yl)acetohydrazide (**2**) and different substituted benzaldehydes and salicylaldehydes (**3a**–**3f**) (Scheme 1). The products were obtained in yields ranging from 50% to 80%, with compound **1b** showing the lowest yield. The 2-(2-methyl-4-oxoquinazolin-3(4*H*)-yl)acetohydrazide was prepared according to the protocol previously described [32].

The FTIR analysis confirmed the condensation by the disappearance of the bands specific to the stretching (ν) and bending (δ) vibrations of NH_2_ group of 2-(2-methyl-4-oxoquinazolin-3(4*H*)-yl)acetohydrazyde (ν 3300 and δ 1642 cm^−1^) in the spectra of the compounds. The imine band appears at about 1608–1592 cm^−1^, as well as characteristic bands for the substituents of the benzaldehyde ring are present: stretching vibrations for the hydroxyl group (ν 3315–3223 cm^−1^), the nitro group (ν_asym_ 1520 cm^−1^, ν_sym_ 1343 cm^−1^), the methoxy group (ν_asym_ 1087 cm^−1^, ν_sym_ 1037 cm^−1^).

NMR spectroscopy elucidated the isomers present as result of the *N*-acyl-hydrazone bond. The ^1^H-NMR spectra recorded in DMSO-d_6_ reveal a singular imine signal at δ_H_ 8 ppm, which confirms the formation of the *E* isomer on the imine bond. The *Z* isomer, being more hindered, would exhibit its imine signal at δ_H_ 14 ppm [33,34]. The *N*-acyl-hydrazones may exist as two *syn-anti* isomers on the amide bond. Their presence is indicated by the double signals for the imine group (δ_H_ 8.0–8.6 ppm, δ_C_ 141.9–147.5 ppm), the methylene group (δ_H_ 4.8–5.3 ppm, δ_C_ 45.2–46.2 ppm), the NH of the amide group (δ_H_ 11.6–12.0 ppm), and the methyl group (δ_H_ 2.50–2.55 ppm, δ_C_ 22.8–23.5 ppm) in ^1^H-NMR and ^13^C-NMR spectra.

**Figure 2:**
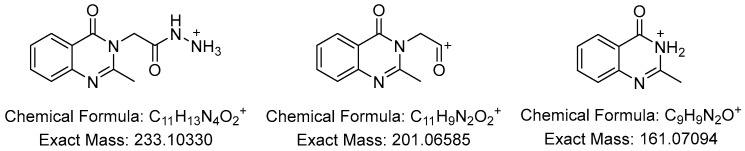
Fragments frequently observed in the APCI+ mass spectra of *N*-acyl-hydrazones **1a**–**1f**.

All the APCI+ high resolution mass spectra were recorded in a mixture of dimethylsulfoxide (DMSO) and methanol (MeOH), due to the low solubility of the compounds. The [M + H]^+^ molecular peaks were observed as base peaks in all the mass spectra, which confirm the identity of the synthesized compounds. For some of the compounds, other fragments were generated during the measurements. In the spectrum of **1c** the peak corresponding to the [M − Br]^+^ cation was also observed. The most frequent peaks appear at *m/z* 233.10, 201.06 and 161.07, corresponding to the fragments depicted on Figure 2.

***N′*-[(*E*)-(5-bromo-2-hydroxyphenyl)methylidene]-2-(2-methyl-4-oxoquinazolin-3(4*H*)-yl)acetohydrazide (1a)**

Appearance: white powder. Yield (%): 76. m.p (°C): 271–272. Rf: 0.41. Solubility: DMSO, DMF, acetone—soluble; methanol—slightly soluble; water—insoluble.

FT-IR (ATR, cm^−1^, solid sample): 3223w, 3171w, 3061w, 2968w, 1700s, 1657vs, 1596s, 1472m, 1439m, 1409m, 1388s, 1342m, 1274s, 1208m, 1182m, 1144w, 1075w, 1035w, 986m, 962w, 942w, 921m, 874w, 855w, 833m, 775vs, 695m, 679m, 660m, 624vs.

^1^H-NMR (DMSO-d6, δ ppm, J Hz): 11.83 (s, 1H, H-13); 10.41 (s, 1H, OH); 8.42 (s, 1H, Hm-15); 8.32 (s, 1H, H_M_-15); 8.09 (bd, 8.2, 1H, H-6); 7.91 (d, 2.6, 1H, H_M_-21); 7.82 (td, 1.2, 7.0, 1H, H-8); 7.70 (d, 2.6, 1H, H_m_-21); 7.62 (bd, 8.2, 1H, H-9); 7.50 (t, 7.9, 1H, H-7); 7.42 (dd, 2.6, 8.3, 1H, H_m_-19); 7.39 (m, 1H, H_M_-19); 6.89 (d, 8.8, 1H, H_M_-18); 6.88 (d, 8.8, 1H, H_m_-18); 5.32 (s, 2H, H_M_-11); 4.90 (s, 2H, H_m_-11); 2.56 (s, 3H, CH_3m_); 2.52 (s, 3H, CH_3M_).

^13^C-NMR (DMSO-d6, δ ppm): 168.59 (C-12); 161.66 (C-4); 156.03 (C-17); 155.91 (C-2); 147.64 (C-10); 145.36 (C_m_-15); 140.08 (C_M_-15); 134.96 (C-8); 134.04 (C-19); 128.16 (C-21); 127.03 (C-9); 126.79 (C-7); 126.64 (C-6); 122.90 (C_M_-5); 121.63 (C_m_-5); 120.06 (C-16); 119.04 (C_m_-18); 118.87 (C_M_-18); 111.34 (C_M_-20); 110.97 (C_m_-20); 46.17 (C_m_-11); 45.73 (C_M_-11); 23.47 (CH_3m_); 23.28 (CH_3M_).

MS: Chemical Formula C_18_H_15_BrN_4_O_3_, Exact Mass 414.03275, HRMS (APCI+, DMSO + MeOH), *m/z* calcd. for [M + H]^+^ 415.04003, found 415.03995 (100%, mass error ∆m = −0.19 ppm).

***N′*-[(*E*)-(3,5-bromo-2-hydroxyphenyl)methylidene]-2-(2-methyl-4-oxoquinazolin-3(4*H*)-yl)acetohydrazide (1b)**

The product was purified from anhydrous ethanol. Appearance: White powder. Yield (%): 50. m.p (°C): 280.9–281.6. Rf: 0.38. Solubility: DMSO, DMF, acetone—soluble; methanol, 2-propanol—slightly soluble; water—insoluble

FT-IR (ATR, cm^−1^, solid sample): 3312w, 3188w, 3072w, 2978w, 2894w, 1690s, 1666vs, 1608m, 1543w, 1512w, 1471m, 1445m, 1413m, 1392w, 1363w, 1341w, 1301m, 1282w, 1248w, 1222m, 1174w, 1144w, 1087w, 1028w, 991w, 976m, 901w, 875m, 767s, 734m, 712m, 693m.

^1^H-NMR (DMSO-d6, δ ppm, J Hz): 12.52 (s, 1H, OH); 12.03 (s, 1H, H-13); 8.37 (s, 1H, H_M_-15); 8.30 (s, 1H, H_m_-15); 8.09 (dd, 1.5, 7.9, 1H, H-6); 7.89 (d, 2.3, 1H, H_M_-21); 7.75–7.86 (m, 2H, H-19, H-8); 7.62 (bd, 9.7, 1H, H-9); 7.52 (t, 7.9, 1H, H-7); 5.33 (s, 2H, H_m_-11); 4.93 (s, 2H, H_M_-11); 2.56 (s, 3H, CH_3m_); 2.52 (s, 3H, CH_3M_). *Syn-anti* ratio: 4:3.

^13^C-NMR (DMSO-d6, δ ppm): 168.07 (C-12); 163.90 (C-17); 161.18 (C-4); 155.39 (C_m_-2); 155.22 (C_M_-2); 147.09 (C-10); 146.76 (C_m_-15); 141.48 (C_M_-15); 135.69 (C_M_-19); 135.61 (C_m_-19); 134.63 (C_M_-8); 134.54 (C_m_-8); 129.28 (C-21); 126.57 (C-7); 126.45 (C-9); 126.13 (C-6); 123.88 (C-16); 120.87 (C_m_-5); 119.54 (C_M_-5); 112.75 (C_m_-20); 111.70 (C_M_-20); 111.19 (C_m_-18); 110.50 (C_M_-18); 45.72 (C_M_-11); 44.78 (C_m_-11); 23.01 (CH_3M_); 22.81 (CH_3m_).

MS: Chemical Formula C_18_H_14_Br_2_N_4_O_3_, Exact Mass 491.94327, HRMS (APCI+, DMSO + MeOH), *m/z* calcd. for [M + H]^+^ 494.94850, found 494.94888 (100%, mass error ∆m = +0.77 ppm), calcd. for [C_11_H_13_N_4_O_2_]^+^ 233.10330, found 233.10355 (99%, mass error ∆m = +1.07 ppm).

***N′*-[(*E*)-(2-hydroxy-3-methoxyphenyl)methylidene]-2-(2-methyl-4-oxoquinazolin-3(4*H*)-yl)acetohydrazide (1c)**

Appearance: White powder. Yield (%): 79. m.p (°C): 221.6–222.8. Rf: 0.38. Solubility: DMSO, DMF, acetone—soluble; methanol—slightly soluble; water—insoluble

FT-IR (ATR, cm^−1^, solid sample): 3312w, 3162w, 2966w, 2883w, 2672w, 1686vs, 1672s, 1602s, 1572s, 1545m, 1522m, 1511m, 1493m, 1471s, 1461s, 1435m, 1417m, 1392s, 1352m, 1339m, 1323m, 1284w, 1255vs, 1209s, 1175m, 1114w, 1087m, 1037m, 994m, 976m, 944m, 878m, 838m, 789s, 766s, 739s, 724s.

^1^H-NMR (DMSO-d_6_, δ ppm, J Hz): 11.78 (s, 1H, H-13); 10.58 (s, 1H, OH); 8.47 (s, 1H, H_m_-15); 8.42 (s, 1H, H_M_-15); 8.12 (dd, 1.2, 7.6, 1H, H-6); 7.82 (td, 0.9, 8.0, 1H, H-8); 7.62 (bd, 8.2, 1H, H-9); 7.49 (t, 7.3, 1H, H-7); 7.39 (dd, 1.2, 7.9, 1H, H-21); 7.12 (d, 7.9, 1H, H_m_-21); 7.00 (d, 8.2, 1H, H-19); 6.83 (td, 1.4, 7.9, 1H, H-20); 5.29 (s, 2H, H_m_-11); 4.89 (s, 2H, H_M_-11); 3.83 (s, 3H, OCH_3M_); 3.79 (s, 3H, OCH_3m_); 2.55 (s, 3H, CH_3m_); 2.52 (s, 3H, CH_3M_).

^13^C-NMR (DMSO-d_6_, δ ppm): 168.39 (C-12); 161.67 (C-4); 155.91 (C_M_-2); 155.78 (C_m_-2); 148.48 (C-18); 147.62 (C-10); 146.50 (C-17); 142.10 (C-15); 134.98 (C-8); 127.02 (C-7); 126.81 (C-9); 126.64 (C-6); 120.88 (C-5); 120.05 (C-21); 119.65 (C-21); 119.35 (C-16); 118.04 (C-20); 114.26 (C_m_-19); 113.51 (C_M_-19); 56.34 (OCH_3_); 46.14 (C_m_-11); 45.64 (C_M_-11); 23.45 (CH_3m_); 23.28 (CH_3M_).

MS: Chemical Formula C_19_H_18_N_4_O_4_, Exact Mass 366.13281, HRMS (APCI+, DMSO + MeOH), *m/z* calcd. for [M + H]^+^ 367.14008, found 367.14030 (100%, mass error ∆m = +0.60 ppm), calcd. for [C_11_H_9_N_2_O_2_]^+^ 201.06585, found 201.06604 (96%, mass error ∆m = +0.94 ppm).

***N′*-[(*E*)-(2-hydroxy-4-methoxyphenyl)methylidene]-2-(2-methyl-4-oxoquinazolin-3(4*H*)-yl)acetohydrazide (1d)**

The product was purified from anhydrous ethanol. Appearance: White powder. Yield (%): 71. m.p (°C): 233.6–234.3. Rf: 0.73. Solubility: DMSO, DMF, acetone—soluble; methanol—slightly soluble; water—insoluble

FT-IR (ATR, cm^−1^, solid sample): 3281w, 3194w, 3048w, 2994w, 1682s, 1632m, 1602s, 1567m, 1508w, 1471w, 1441w, 1411w, 1385w, 1343m, 1283m, 1237s, 1184w, 1162w, 1131w, 1110w, 1027m, 990w, 960m, 868w, 822w, 798w, 772s.

^1^H-NMR (DMSO-d_6_, δ ppm, J Hz): 12.00 (s, 1H, H_m_-13); 11.64 (s, 1H, H_M_-13); 11.24 (s, 1H, H-13); 10.19 (s, 1H, OH); 8.37 (s, 1H, H_m_-15); 8.29 (s, 1H, H_M_-15); 8.09 (dd, 1.5, 7.9, 1H, H-6); 7.82 (td, 1.5, 8.2, 1H, H-8); 7.67 (d, 8.5, 1H, H_M_-21); 7.62 (bd, 8.2, 1H, H-9); 7.50 (t, 7.6, 1H, H-7); 7.47 (d, 8.4, 1H, H_m_-21); 7.44 (d, 8.5, 1H, H-21); 6.45–6.54 (M, 2H, H-18, H-20); 5.27 (s, 2H, H_M_-11); 4.88 (s, 2H, H_m_-11); 3.75 (s, 3H, OCH_3m_); 3.35 (s, 3H, OCH_3M_); 2.56 (s, 3H, CH_3M_); 2.51 (s, 3H, CH_3m_). *Syn-anti* ratio: 3:4

^13^C-NMR (DMSO-d_6_, δ ppm): 167.53 (C-12); 162.14 (C-4); 162.00 (C-4); 161.18 (C-19); 159.19 (C-17); 155.45 (C_M_-2); 155.32 (C_m_-2); 147.97 (C-15); 147.16 (C-10); 142.21 (C-15); 134.55 (C-8); 134.49 (C-21); 126.55 (C-7); 126.38 (C-9); 126.32 (C-6); 119.58 (C-5); 112.95 (C-16); 111.61 (C-16); 106.44 (C-20); 101.09 (C-18); 100.88 (C-18); 55.28 (OCH_3_); 55.17 (OCH_3_); 45.61 (C_m_-11); 45.10 (C_M_-11); 22.99 (CH_3m_); 22.81 (CH_3M_).

MS: Chemical Formula C_19_H_18_N_4_O_4_, Exact Mass 366.13281, HRMS (APCI+, DMSO + MeOH), *m/z* calcd. for [M + H]^+^ 367.14008, found 367.14035 (100%, mass error ∆m = +0.73 ppm), calcd. for [C_11_H_9_N_2_O_2_]^+^ 201.06585, found 201.06622 (31%, mass error ∆m = +1.84 ppm).

***N′*-[(*E*)-(2-hydroxy-5-methoxyphenyl)methylidene]-2-(2-methyl-4-oxoquinazolin-3(4*H*)-yl)acetohydrazide (1e)**

Appearance: White powder. Yield (%): 61. m.p (°C): 245.4–246.8. Rf: 0.39. Solubility: DMSO, DMF—soluble; methanol—slightly soluble; water—insoluble

FT-IR (ATR, cm^−1^, solid sample): 3315w, 3218w, 3079w, 3008w, 2970w, 2834w, 1680vs, 1592m, 1573m, 1493s, 1472m, 1462m, 1434m, 1413m, 1388s, 1344m, 1311m, 1276vs, 1250m, 1220w, 1180m, 1159m, 1085w, 1038m, 985m, 942m, 863m, 826m, 804m, 770s.

^1^H-NMR (DMSO-d_6_, δ ppm, J Hz): 12.06 (s, 1H, H_m_-13); 11.76 (s, 1H, H_M_-13); 10.37 (s, 1H, OH_m_); 9.63 (s, 1H, OH_M_); 8.44 (s, 1H, H_m_-15); 8.35 (s, 1H, H_M_-15); 8.10 (dd, 1.5, 7.9, 1H, H-6); 7.82 (td, 1.5, 7.8, 1H, H-8); 7.63 (bd, 7.9, 1H, H-9); 7.50 (td, 7.9, 1.1, 1H, H-7); 7.31 (d, 2.6, 1H, H_M_-21); 7.12 (d, 2.9, 1H, H_m_-21); 6.82–6.87 (m, 2H, H-18, H-19); 5.31 (s, 2H, H_M_-11); 4.90 (s, 2H, H_m_-11); 3.71 (s, 3H, OCH_3M_); 3.34 (s, 3H, OCH_3m_); 2.56 (s, 3H, CH_3M_); 2.51 (s, 3H, CH_3m_). *Syn-anti* ratio: 1:2

^13^C-NMR (DMSO-d_6_, δ ppm): 168.42 (C-12); 163.85 (C-19); 161.67 (C-4); 155.89 (C-2); 152.73 (C_M_-17); 152.61 (C_m_-17); 151.79 (C_m_-20); 151.15 (C_M_-20); 147.4 (C-10); 147.18 (C_m_-15); 141.93 (C_M_-15); 134.96 (C-8); 127.03 (C-7); 126.80 (C-9); 126.63 (C-6); 120.75 (C-5); 120.06 (C-5); 119.35 (C-16); 118.54 (C-19); 117.65 (C-18); 112.26 (C_m_-21); 109.99 (C_M_-21); 55.92 (OCH_3_); 46.17 (C_m_-11); 45.69 (C_M_-11); 23.47 (CH_3m_); 23.30 (CH_3M_).

MS: Chemical Formula C_19_H_18_N_4_O_4_, Exact Mass 366.13281, HRMS (APCI+, DMSO + MeOH), *m/z* calcd. for [M + H]^+^ 367.14008, found 367.14055 (100%, mass error ∆m = +1.28 ppm), calcd. for [C_11_H_9_N_2_O_2_]^+^ 201.06585, found 201.06623 (68%, mass error ∆m = +1.89 ppm).

***N′*-[(*E*)-(4-hydroxy-3-methoxy-5-nitrophenyl)methylidene]-2-(2-methyl-4-oxoquinazolin-3(4*H*)-yl)acetohydrazide (1f)**

Appearance: Orange powder. Yield (%): 80. m.p (°C): 281.0–281.3. Rf: 0.32. Solubility: DMSO, DMF, acetone—soluble; methanol—slightly soluble; water—insoluble

FT-IR (ATR, cm^−1^, solid sample): 3236w, 3121w, 3062w, 2973w, 2902w, 2832w, 1699vs, 1657vs, 1601m, 1554w, 1533vs, 1511m, 1471m, 1461w, 1409m, 1390m, 1341m, 1291s, 1255s, 1182m, 1145w, 1120w, 1066w, 1031w, 985w, 949w, 916m, 865m, 825w, 807w, 773vs.

^1^H-NMR (DMSO-d_6_, δ ppm, J Hz): 11.89 (s, 1H, H-13); 8.19 (d, 1.4, 1H, H_m_-21); 8.09 (dd, 1.5, 7.9, 1H, H-6); 8.02 (s, 1H, H-15); 7.82 (td, 1.5, 7.0, 1H, H-8); 7.80 (d, 2.0, 1H, H_M_-21); 7.63 (d, 2.0, 1H, H_M_-17); 7.62 (bd, 6.9, 1H, H-9); 7.56 (d, 1.7, 1H, H_m_-17); 7.50 (td, 1.1, 8.2, 1H, H-7); 5.34 (s, 2H, H_M_-11); 4.90 (s, 2H, H_m_-11); 3.95 (s, 3H, OCH_3M_); 3.92 (s, 3H, OCH_3m_); 2.55 (s, 3H, CH_3m_); 2.52 (s, 3H, CH_3M_).

^13^C-NMR (DMSO-d_6_, δ ppm): 168.23 (C_M_-12); 168.12 (C_m_-12); 161.20 (C-4); 155.39 (C-2); 149.75 (C-10); 147.14 (C-10); 143.94 (C-19); 142.72 (C-15); 137.22 (C-20); 134.51 (C-8); 126.56 (C-9); 126.35 (C-7); 126.15 (C-6); 124.64 (C-16); 119.56 (C-5); 116.25 (C_m_-17); 115.47 (C_M_-17); 112.65 (C_M_-21); 112.08 (C_m_-21); 56.69 (OCH_3_); 45.66 (C-11_m_); 45.30 (C-11_M_); 22.96 (CH_3m_); 22.84 (CH_3M_).

MS: Chemical Formula C_19_H_17_N_5_O_6_, Exact Mass 411.11788, HRMS (APCI+, DMSO + MeOH), *m/z* calcd. for [M + H]^+^ 412.12516, found 412.12582 (100%, mass error ∆m = +1.60 ppm).

All corresponding FT-IR, NMR and MS spectra are presented in the Figures S1–S24.

### 2.2. Prediction of Physicochemical Properties and ADMET Profile

The druglikeness and leadlikeness properties for compounds **1a**–**1f** were determined using SwissADME web tool [35] and are presented in Table S1. Except for **1f**, all derivatives comply with Lipinski rule of five, Veber rule, Egan rule, and Muegge rule for druglike molecules. In the case of Ghose criteria, the only incriminated molecule is **1b**, which has a molecular weight over than 480. Compound **1f** has a total polar surface area above 130 and a total number of N and O atoms greater than ten, which makes it unsuitable for oral drug absorption. The only violation of the leadlikeness criterion is molecular weight, which exceeds the limit of 350. Figure S25 presents the predicted solubility and lipophilicity of compounds **1a**–**1f**. *N*-acyl-hydrazones are lipophilic (logP > 1.5) and present low solubility (logS < −3 mol/L), compound **1b** with two bromine atoms has the lowest parameters among the series.

Regarding pharmacokinetics, Table S2 summarizes the most important parameters. Compounds **1a**–**1e** are predicted to exhibit high gastrointestinal absorption, whereas the nitro-substituted analogue **1f** showed markedly reduced uptake. None of the *N*-acyl-hydrazones displays measurable affinity for P glycoprotein (P-gp), and all are predicted to exhibit negligible permeability across the blood-brain barrier (BBB) and the skin. All derivatives, except **1f**, may be inhibitors of CYP2C9, whereas halogen containing compounds may inhibit CYP1A2 (**1b**) and CYP3A4 (**1a** and **1b**) as well. All compounds present low body clearance, especially **1a** and **1b**. In terms of toxicity, the derivatives **1b** and **1f** are the only ones incriminated as cardiotoxic, whereas **1f** is also mutagenic. Hepatotoxicity may be considered for all compounds, except for **1a**.

In order to identify the potential targets and activities for the new series of *N*-acyl-hydrazones **1a**–**1f**, a in silico target determination was applied using three web services: SwissTargetPrediction [36], SuperPred database [37] and PassOnline [38]. The top target predictions thus obtained are presented in Figure S26, Tables S3–S5.

SwissTargetPrediction indicates proteases, family A G protein-coupled receptor, kinases and oxidoreductases as the most frequent targets (Figure S26a). There are indicated more than one enzyme implicated in cancer, such as telomerase reverse transcriptase, sphingosine kinase 2, pyruvate dehydrogenase kinase isoform 1, tyrosine-protein kinase receptor FLT3 (Table S3). On the other hand, SuperPred focused on casein kinase II alpha/beta, DNA-lyase, protein Mdm4, DNA topoisomerase II alpha as possible cancer targets, and also Ras-related protein Rab-9A which is involved in fungal infection (Figure S26b, Table S4).

In addition, PassOnline indicates both possible anticancer and antimicrobial activities: antitubercular, antineoplastic (bone cancer, brain cancer, multiple myeloma), antiprotozoal (*Trichomonas*, *Coccidial*, *Amoeba*) (Table S5).

### 2.3. Total Antioxidant Capacity (TAC)

Antioxidant capacity can influence anticancer or antimicrobial activity, but it’s not predictive on its own [39,40]. Even in the absence of a direct relationship, in many cases, antioxidant compounds do exhibit anticancer properties, possibly due to dual roles in redox balance and cell signalling [41,42,43]. The overall chemical structure, target pathways, and cellular context are significant factors that influence these properties [42,44].

The total antioxidant capacity (TAC) was evaluated using diphenyl-1-picrylhydrazyl free radical (DPPH) method. The TAC value was determined in relation to the antioxidant capacity of the reference (ascorbic acid, TAC = 100%). All tested new compounds, as well as 2-(2-methyl-4-oxoquinazolin-3(4*H*)-yl)acetohydrazide (**2**) exhibited some antioxidant activity (Table 1). The maximum value was recorded for **1c** (~45%), followed by **1f** and **1a** (~43%), while the minimum for **1e** (~19%).

### 2.4. Antimicrobial Evaluation

Given the escalating problem of microbial resistance and the urgent need for innovative antimicrobial agents, evaluating the antimicrobial activity of newly synthesized chemical derivatives is a critical step toward developing novel therapeutic strategies that address current medical challenges [45,46,47].

**Figure 3:**
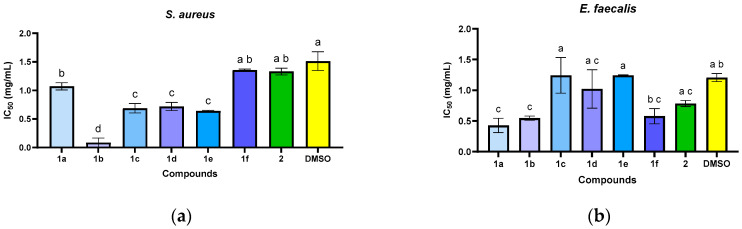

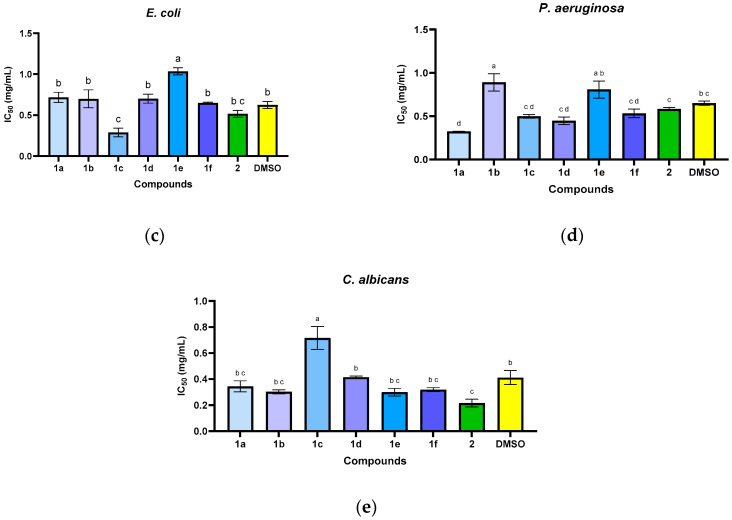
The antimicrobial activity (IC_50_) of *N*-acyl-hydrazones **1a**–**1f** against *S. aureus* (**a**), *E. faecalis* (**b**), *E. coli* (**c**), *P. aeruginosa* (**d**), and *C. albicans* (**e**). The letters (a–d) above the bars in the graph indicate significant differences between the samples (*p <* 0.05).

The antimicrobial and antibiofilm activities of *N*-acyl-hydrazones **1a**–**1f** against *S. aureus* (a), *E. faecalis* (b), *E. coli* (c), *P. aeruginosa* (d), and *C. albicans* (e) are presented in Table 2 and Figure 3. In qualitative testing, antimicrobial activity was evaluated by inhibition zone diameter (IZD), which was recorded in the case of two derivatives. Compound **1c** was active against *S. aureus* (IZD = 7.67 ± 0.58 mm), *E. faecalis* (7.67 ± 0.58 mm), and *C. albicans* (6.67 ± 0.58 mm), while derivative **1b** inhibited *S. aureus* (12.33 ± 0.58 mm). On the other hand, the 2-(2-methyl-4-oxoquinazolin-3(4*H*)-yl)acetohydrazide (**2**) showed activity against *C. albicans* (6.33 ± 1.53 mm).

In the quantitative assays, the compounds exhibited specific antimicrobial activity, with minimum inhibitory concentrations (MICs) ranging from 0.3125 to 5 mg/mL, depending on the compound structure and the tested microbial strain. Some compounds showed a narrow spectrum of action, being specifically active against Gram-positive bacteria (*S. aureus*: **1a**, **1b**, **1e**; *E. faecalis*: **1a**, **1f**) and yeasts (*C. albicans*: **1a**, **1b**). The most active compound against *S. aureus* was **1b** (MIC = 0.3125 mg/mL). The results were inferior to those of the reference drugs.

Regarding the minimum microbicidal concentrations (MMC) values, in the case of the *S. aureus* strain, none of the tested compounds exhibited a more pronounced microbicidal effect compared to the solvent, while for *E. faecalis*, the most active compound was **1a** (highlighted in blue, Table 2), but with higher values than the MIC. In the case of the *E. coli* strain, some compounds exhibited a microbicidal effect at concentrations lower than the solvent used: **1a**, **1b**, **1f** and **2**. The microbicidal effect on the *P. aeruginosa* strain was observed only for **1d**, at concentrations similar to the MIC values. The microbicidal activity against the *C. albicans* strain was observed only for compound **1e**. For the remaining compounds, the microbicidal effect was due to the solvent.

The influence of the newly synthesized compounds on microbial adherence, the initial stage of microbial biofilm development [48] was lastly evaluated. In the case of the *S. aureus, E. faecalis*, and *E. coli* strains, no compound inhibited adherence (Table 2). In the case of the *P. aeruginosa* strain, only compound **1a** inhibited biofilm formation (MICMA values lower than the MIC and the MICMA for DMSO), whereas for the *C. albicans* cells, adherence inhibition was observed only for compounds **1a** and **1c** (Table 2).

The study included ciprofloxacin and ketoconazole as reference drugs used in clinical practice to validate the approach and enable a pertinent comparison of the antibacterial activity of the synthesised derivatives. The benchmark for assessing antibacterial activity was ciprofloxacin (100 µg/mL), a broad-spectrum antibiotic from the fluoroquinolone family that acts on both Gram-positive and Gram-negative bacteria. The antifungal activity against *C. albicans* was tested using ketoconazole (140 µg/mL), an antifungal belonging to the imidazole class, as a reference. With notable inhibition zones (e.g., 34.00 ± 2.00 mm for *E. coli*) and MIC values ranging from 0.20 to 0.78 µg/mL, ciprofloxacin demonstrated superior antimicrobial activity when compared to DMSO, confirming the effectiveness of the procedure and the parameters employed. Ketoconazole showed antifungal activity against *C. albicans*, with a MIC of 8.75 µg/mL and an inhibition zone of 6.5 ± 0.71 mm. The reference drugs’ strong activity validated the methodology and offered a framework for comparison in assessing the tested compounds.

Through the statistical analysis of the inhibitory concentration 50% (IC_50_) values (Figure 3), it was observed that some of the compounds have significantly higher antibacterial activity than the solvent used (DMSO, *p <* 0.05) in the case of the *S. aureus* strain (Figure 3a), manifested in the order: **1b** > **1e** > **1c** > **1d** > **1a** > **2** > **1f** > DMSO. The *E. faecalis* strain differences are not significant (Figure 3b) compared to the solvent used for compound **1a** (*p >* 0.05). For the *E. coli* strain, it can be seen that only compound **1c** showed significantly lower IC_50_ values than the solvent control (Figure 3c). The IC_50_ values were not significantly different from the solvent for the *P. aeruginosa* strain (Figure 3d), except for compound **1a**.

The compounds demonstrated inhibitory effects against *C. albicans* strain (Figure 3e) in the following descending order of potency: **2** > **1e** > **1b** > **1f** > **1a** > DMSO > **1d** > **1c**.

**Figure 4:**
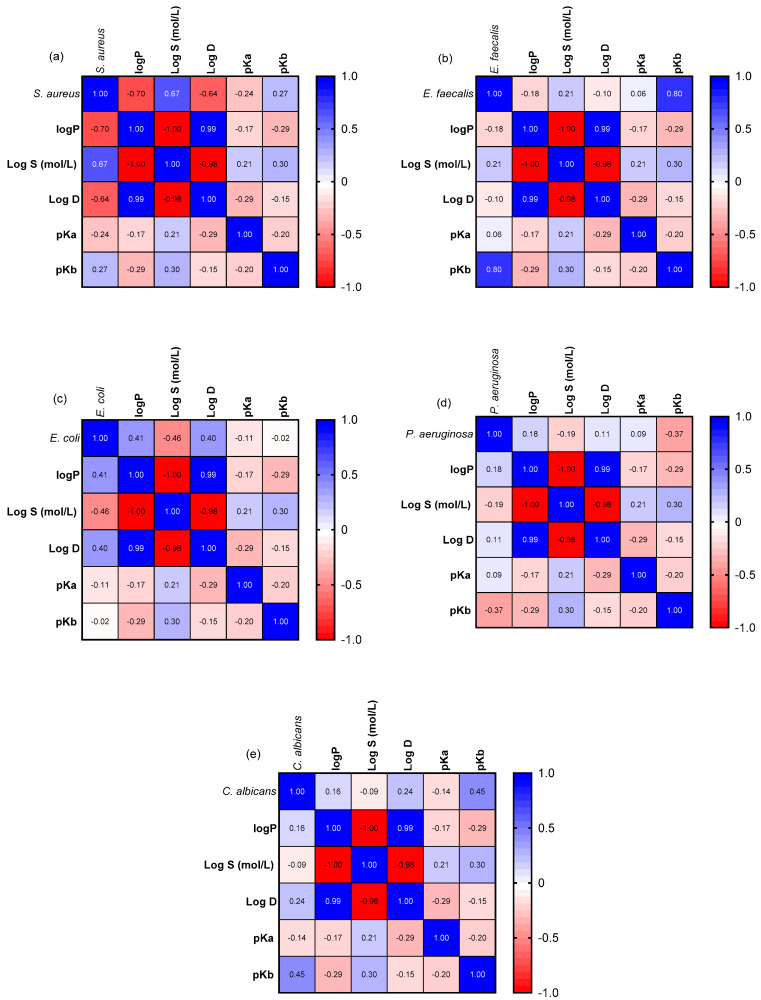
Pearson correlation matrix illustrating the relationship between physicochemical parameters and antimicrobial activity of *N*-acyl-hydrazones **1a**–**1f** against *S. aureus* (**a**), *E. faecalis* (**b**), *E. coli* (**c**), *P. aeruginosa* (**d**), *C. albicans* (**e**).

To enhance understanding of the relationship between structural properties and antimicrobial activity, a Pearson correlation matrix was employed (Figure 4). The correlation between physicochemical attributes (LogP, LogD, and solubility) and antibacterial efficacy (IC_50_ values) varies according to the bacterial species examined. Lipophilicity correlates positively with antibacterial activity, as evidenced by the low to significant negative correlation between IC_50_ values and LogP for both Gram-positive bacteria, *S. aureus* (Figure 4a) and *E. faecalis* (Figure 4b). A similar negative correlation was observed between LogD values. Conversely, solubility (LogS) demonstrated a positive correlation (r = 0.67 for *S. aureus* and r = 0.21 for *E. faecalis*), indicating that lower IC_50_ values are associated with reduced solubility. This trend aligns with prior reports emphasizing the influence of lipid solubility on membrane permeability in Gram-positive bacteria [49,50,51].

Gram-negative bacteria, such as *E. coli* and *P. aeruginosa*, showed an opposite pattern. Water solubility and IC_50_ showed a significant negative correlation, r = −0.46 for *E. coli* (Figure 4c) and r = −0.19 for *P. aeruginosa* (Figure 4d), indicating that higher solubility enhances antibacterial activity. Lipophilicity (LogP) showed limited or negligible positive relationships, implying that high lipophilicity had no significant effect on antibacterial efficacy against Gram-negative bacteria. Concerning the fungal strain *C. albicans* (Figure 4e), a weak negative correlation was demonstrated between IC_50_ values and LogP (r = 0.16) and LogD (r = 0.24), similar to Gram-negative bacteria.

### 2.5. Cytotoxicity Evaluation Against Normal and Cancer Cell Lines

The cytotoxic potential of *N*-acyl-hydrazones **1a**–**1f** and compound **2** was evaluated by MTT assay after 24 h of treatment on two cancer cell lines, HT-29 (human colorectal adenocarcinoma) and A431 (human epidermoid carcinoma), as well as on the normal kidney epithelial cell line VERO, to assess their selectivity. The compounds were benchmarked against the reference drug 5-fluorouracil (5-FU).

**Figure 5:**
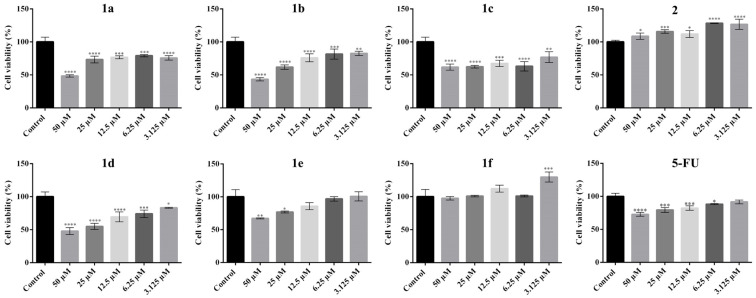
Effect of *N*-acyl-hydrazones derivatives **1a**–**1f**, **2**, and 5-FU on HT-29 tumor cell viability after 24 h of treatment as revealed by the MTT assay. Cell viability is expressed as a percentage relative to the untreated control cells (100% cell viability). Data are presented as mean ± SD of three independent experiments performed in triplicate. Statistical significance: * *p ≤* 0.05, ** *p ≤* 0.01, *** *p ≤* 0.001, **** *p ≤* 0.0001 treatment vs. control.

In HT-29 cell cultures (Figure 5), compounds **1a**–**1d** exhibited a significant and concentration-dependent decrease in cell viability compared to the untreated control. All four compounds demonstrated strong cytotoxic effects against human colorectal adenocarcinoma cells, as cell viability was significantly reduced at all tested concentrations. Compound **1a** exhibited a pronounced cytotoxic effect, reducing cell viability to approximately 50% at 50 µM (IC_50_) compared to the control. At lower concentrations (3.125–25 µM), the cytotoxic effect remained significant and relatively uniform, suggesting a plateau in the dose–response relationship within this range. Similarly, compound **1b** exhibited marked cytotoxicity, particularly at concentrations of 50 µM and 25 µM, where cell viability was reduced by nearly 50% compared to the control. The effect at lower concentrations was also evident but did not differ significantly among them. Compound **1c** exhibited potent inhibitory effects at all concentrations tested, with statistically significant reductions in viability compared to the control. However, in this case, the tested concentration range did not produce a viability drop sufficient to determine an IC_50_ value.

In the case of compound **1d,** all tested concentrations caused a significant decrease in cell viability, with the most pronounced effects observed at 50 µM and 25 µM, where viability was reduced by approximately 50% (Figure 5). By contrast, compound **1e** displayed weaker cytotoxic activity, with statistically significant reductions in viability observed only at the highest concentrations. Compound **1f** had no inhibitory effect on HT-29 cells; notably, at 3.125 µM, a slight but statistically significant increase in cell viability was observed, suggesting a potential proliferative or stimulatory effect. Finally, compound **2** induced an overall increase in cell viability at all tested concentrations, indicating a lack of anticancer activity on this cell line and possibly a cytoprotective or proliferative influence.

For the 5-FU treatment on HT-29 cells, a concentration-dependent decrease in cell viability was observed, which was statistically significant across higher doses. However, the cytotoxic effect of the reference drug was less pronounced compared to novel derivatives (particularly **1a**, **1b**, **1d**), which reduced cell viability below 50% at corresponding concentrations. At the same time, the IC_50_ for 5-FU was not reached within the tested concentration range.

**Figure 6:**
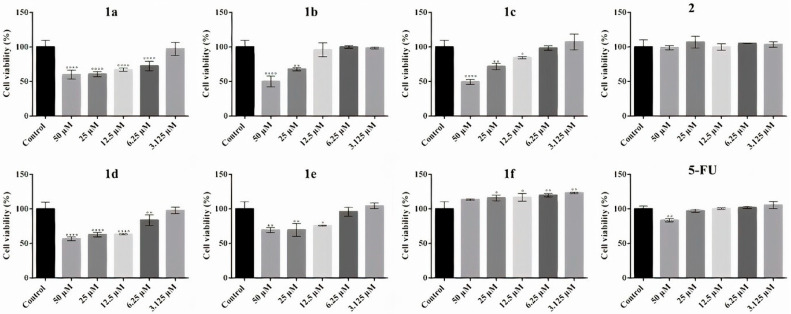
Effect of *N*-acyl-hydrazones derivatives **1a**–**1f**, **2**, and 5-FU on A431 tumor cell viability after 24 h of treatment as revealed by the MTT assay. Cell viability is expressed as percentage relative to the untreated control cells (100% cell viability). Data are presented as mean ± SD of three independent experiments performed in triplicate. Statistical significance: * *p ≤* 0.05, ** *p ≤* 0.01, **** *p ≤* 0.0001 treatment vs. control.

In A431 cell cultures (Figure 6), compounds **1a**–**1d** also demonstrated a concentration-dependent decrease in cell viability, although the cytotoxic effect appeared slightly less pronounced than in HT-29 cells. Compound **1a** significantly reduced cell viability at 50 µM, 25 µM, and 12.5 µM, with the most substantial effect observed at 50 µM, where viability dropped to approximately 60% compared to the control. At lower concentrations (6.25 µM and 3.125 µM), the decrease in viability was less pronounced and not statistically significant. Compound **1b** also exhibited potent cytotoxicity, particularly at 50 µM, where viability was reduced to approximately 50% (IC_50_). A moderate but significant effect was observed at 25 µM, while no relevant cytotoxicity was detected at lower concentrations. Compound **1c** induced a significant reduction in A431 cell viability across all concentrations tested. A dose-dependent response was observed, with viability reduced to approximately 50% at 50 µM (IC_50_), and moderate but statistically significant effects at 25 µM and 12.5 µM. Although the overall cytotoxic profile of compound **1c** mirrors the profile determined in HT-29 cells, the extent of cell viability reduction was slightly intense. Compound **1d** displayed a consistent cytotoxic effect, with statistically significant reductions in viability at all concentrations, most notably at 50 µM and 25 µM, where cell viability dropped below 60%. In contrast, compound **1e** showed only mild cytotoxicity on A431 cells. Statistically significant effects were observed only at 50 µM and 25 µM, with viability remaining above 70% at all doses. Compound **1f** did not exert any inhibitory effect on A431 cells. In fact, a slight but consistent increase in cell viability was noted at all concentrations, statistically significant at 3.125–12.5 µM, suggesting potential proliferative effects. Compound **2** exhibited no cytotoxic activity; cell viability remained comparable to control values across all concentrations, and a subtle increase in viability was observed, though not statistically significant.

Similar to *N*-acyl-hydrazones derivatives, the effect of 5-FU was weaker on A431 cells compared to HT-29. Moreover, certain compounds exerted a stronger cytotoxic response than the reference drug at corresponding concentrations (Figure 6).

**Figure 7:**
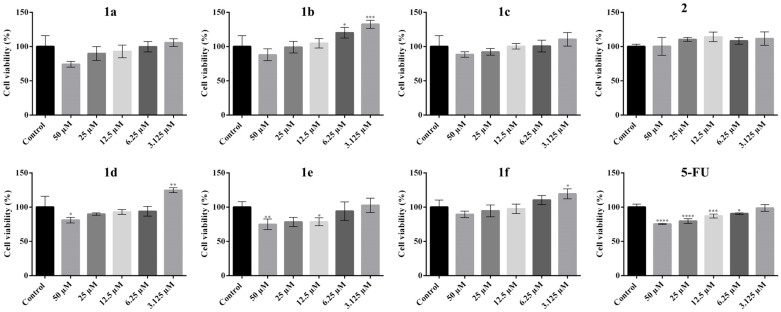
Effect of *N*-acyl-hydrazones derivatives **1a**–**1f**, **2**, and 5-FU on VERO normal cell viability after 24 h of treatment as revealed by the MTT assay. Cell viability is expressed as percentage relative to the untreated control cells (100% cell viability). Data are presented as mean ± SD of three independent experiments performed in triplicate. Statistical significance: * *p ≤* 0.05, ** *p ≤* 0.01, *** *p ≤* 0.001, **** *p ≤* 0.0001 treatment vs. control.

The cytotoxicity profile of the tested compounds was notably different in Vero cell cultures (Figure 7), with most derivatives exhibiting a milder impact compared to cancerous cells. Compounds **1a**–**1d** induced only a slight reduction in cell viability at 50 µM, but among them, only compound **1d** caused a statistically significant decrease in viability compared to the untreated control. Interestingly, for compounds **1b** and **1d**, treatment at lower concentrations resulted in a statistically significant increase in cell viability, suggesting a possible stimulatory effect on normal cells and further supporting the selective cytotoxicity of these compounds against tumor cells. Notably, compound **1e**, which exhibited only mild cytotoxicity on tumor cell lines, showed strong cytotoxic effects on Vero cells. A statistically significant reduction in cell viability was observed at concentrations above 12.5 µM, while no significant effects were detected below this threshold, indicating a lack of selectivity and a potential safety concern for normal tissues. Finally, compounds **1f** and **2** were well tolerated by Vero cells at all tested concentrations. These findings are consistent with their lack of cytotoxicity and the slightly proliferative effects observed in tumor cells, confirming their non-toxic profile.

For 5-FU treatment on Vero cells, a statistically significant decrease in cell viability was observed at higher applied concentrations. The reference drug produced the most potent toxic effect on the normal Vero cells compared to all tested compounds; however, the IC_50_ value was not reached within the tested range, with the maximum reduction in viability being approximately 25% at 50 µM (Figure 7).

### 2.6. Artemia franciscana Toxicity Assay

The toxicity profile of *N*-acyl-hydrazones **1a**–**1f** and 2-(2-methyl-4-oxoquinazolin-3(4*H*)-yl)acetohydrazide (**2**) was evaluated using *Artemia franciscana* Kellogg as the study animal model. Brine shrimp species, such as *Artemia franciscana* Kellogg and *Artemia salina* (L.), are commonly used as alternative animal models in toxicity testing. They present several advantages that recommend them for toxicological assays: commercial availability, no need for ethical approval, a short life cycle, high hatching performance, no need for prior breeding, high sensitivity to toxic substances, and the possibility of working with both small and large sample throughput, thus obtaining rapid results. The nauplii stage is frequently used in acute lethality testing based on the mortality at 24- and 48-h post-treatment [52].

**Figure 8:**
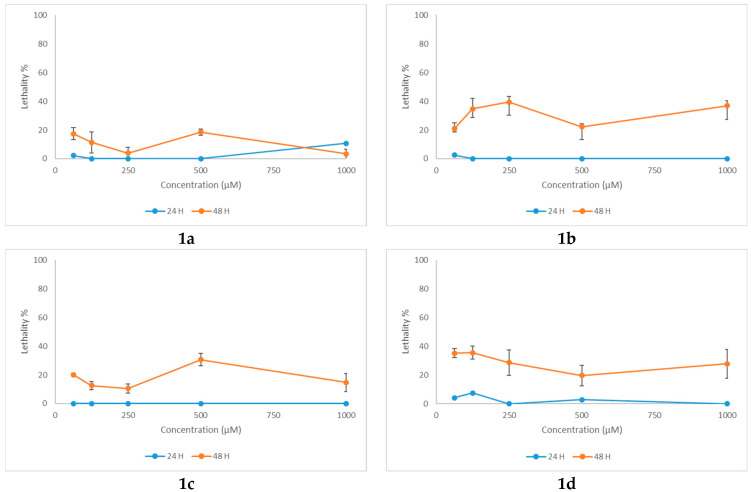

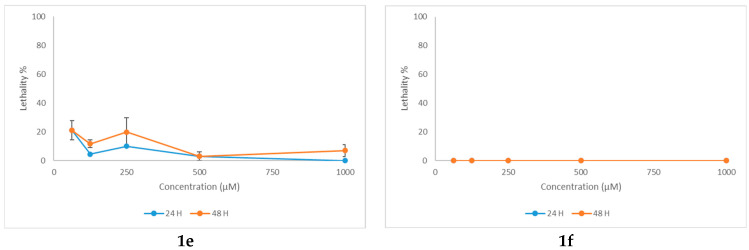
Lethality curves at 24 and 48 h for *N*-acyl-hydrazones **1a**–**1f** against *Artemia franciscana*.

All *N*-acyl-hydrazones **1a**–**1f** exhibited low or negligible mortality at 24 h, with subsequent deaths showing no clear dose-response relationship (Figure 8). At 48 h, mortality rates increased; however, they did not exceed 40%, thus precluding the determination of the lethal concentration 50% (LC_50_). It is noteworthy that all *N*-acyl-hydrazones **1a**–**1f** demonstrated low aqueous solubility, which may have influenced toxicity outcomes by mechanical effects. The positive control, potassium dichromate, had calculated LC_50_ of 53.5 μg/mL at 24 h (95% confidence interval, 95% CI 5.56-101.44 μg/mL), and 5.1 μg/mL (95% CI 2.69-7.61 μg/mL) at 48 h of exposure, respectively (Figure S27).

On the other hand, 2-(2-methyl-4-oxoquinazolin-3(4*H*)-yl)acetohydrazide (**2**) presented a dose-dependent mortality at 48 h (Figure S27). The LC_50_ was 335.4 μM (95% confidence interval, 95% CI 288.1–382.7 μM) or 77.2 mg/L, which corresponds to moderate toxicity based on standard aquatic toxicity classifications [53].

### 2.7. Molecular Docking

A molecular docking study was conducted to elucidate the plausible mechanism of action for the present series of *N*-acyl-hydrazones. The most active compounds were selected and docked against relevant enzymes. The 4-oxoquinazolin-4(3*H*)-one and related 4-hydroxy-2-quinolone-3-carboxamide scaffolds may act as potential pharmacophores for bacterial gyrase B (GyrB) inhibition, with in vitro and docking results supporting their activity [54,55]. Additionally, bacterial dihydrofolate reductase (DHFR) was also considered due to the ability of quinazolinone-hydrazones to act as inhibitors [56]. For antifungal activity, sterol 14α-demethylase (LDM), part of the CYP51 family involved in ergosterol biosynthesis, was included, as it is a known target of azole antifungals [57,58,59]. For anticancer activity, EGFR kinase was analyzed as a relevant target for quinolone derivatives [60,61,62].

#### 2.7.1. Potential Binding Mode of **1b** to DNA Gyrase and DHFR of *S. aureus*

**Figure 9:**
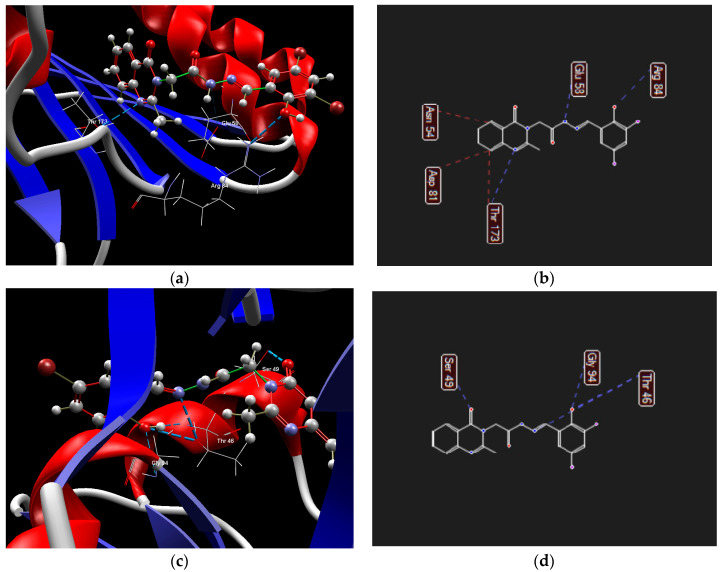
Hydrogen bond (blue dotted lines) and steric interactions (red dotted lines) between **1b** and amino acid residues from the binding site of *S. aureus* gyrase B ((**a**)—3D structure, (**b**)—2D structure) and *S. aureus* DHFR ((**c**)—3D structure, (**d**)—2D structure).

The potential binding mode of the most active antibacterial compound **1b** was determined using *S. aureus* gyrase B in complex with novobiocin (PDB ID: 4URO) [63] and *S. aureus* DHFR in complex with trimethoprim (PDB ID: 2W9H) [64]. The results are presented in Figure 9 and Figures S28 and S29, Tables S6 and S7.

Regarding *S. aureus* gyrase B, compound **1b** presented a lower binding score compared with novobiocin (−91.57 vs. −125.51) (Table S6). The derivative is oriented in the same region as the coumarin core and the sugar fragment of novobiocin (Figure S28). The quinazolin-4(3*H*)-one scaffold occupies the hydrophobic pocket, interacting with Asn54 and Asp81 through steric bonds, and with Thr173 via both steric and hydrogen bonds. The hydrazone group forms hydrogen bonds with Glu58, while the hydroxyl group of the phenyl ring interacts with Arg84, being oriented in the hydrophilic pocket (Figure 9a,b, Table S6).

In the case of *S. aureus* DHFR, compound **1b** exhibited a higher docking score compared to the co-crystallized ligand trimethoprim (−113.49 versus −95.26) (Table S7). Trimethoprim’s diaminopyrimidine ring is positioned within the active site pocket and its phenyl ring resides in an external hydrophobic pocket (Figure S29). Compound **1b,** on the other hand, adopts an opposite orientation. The quinazolin-4(3*H*)-one scaffold interacts with Ser49 at the entrance of the binding site via hydrogen bonds. The *N*-acyl-hydrazone moiety directs the phenyl ring into the hydrophobic pocket, with the imine group forming hydrogen bonds with Thr46, and the *ortho*-hydroxyl group of the phenyl ring engaging with Gly94 (Figure 9c,d).

#### 2.7.2. Potential Binding Mode of Compounds **1b** to Sterol 14α-Demethylase

Compounds **1b** were docked against *C. albicans* sterol 14α-demethylase in complex with posaconazole (PDB ID: 5FSA) [65].

**Figure 10:**
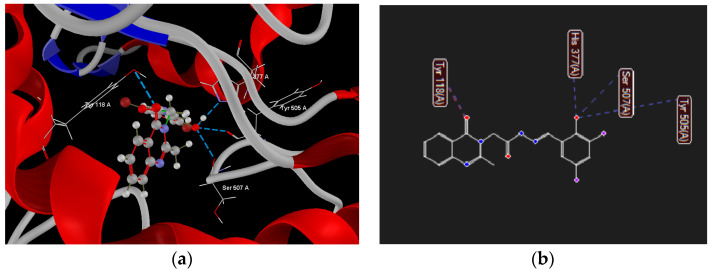
Hydrogen bond (blue dotted lines) and steric interactions (red dotted lines) between compounds **1b** and amino acid residues from the binding site of sterol 14α-demethylase ((**a**)—3D visualization, (**b**)—2D visualization).

Compound **1b** presented a higher binding score than its parent compound **2** (−111.45 vs. −95.45), but inferior to the co-crystrallized ligand, posaconazole, or reference ketoconazole (−175.10, −178.94 respectively) (Table S8). It forms hydrogen bonds with His377, Ser507 and Tyr505 through its hydroxyl group and steric interactions with Tyr118 through the keto group from position 4 of the quinazolinone core (Figure 10). Posaconazole does not form any hydrogen bonds with the aminoacids of the binding site, whereas ketoconazole interacts with Tyr132 via hydrogen bonds and with Ile131 via steric bonds (Table S8 and Figure S30).

#### 2.7.3. Potential Binding Mode of Compounds **1a**–**1e** to the EGFR Kinase

The active compounds **1a**–**1e** were docked against inactive EGFR kinase in complex with lapatinib (PDB ID: 1XKK) [66].

**Figure 11:**
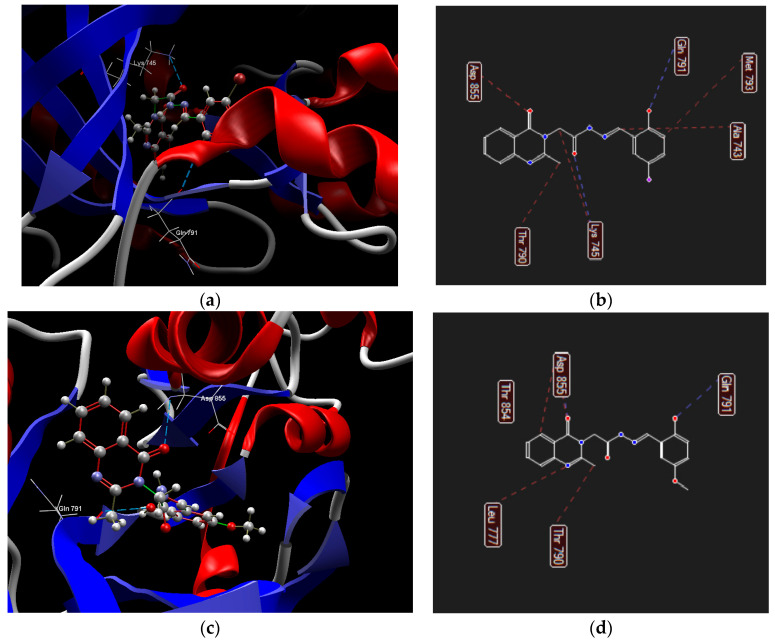
Hydrogen bonds (blue dotted lines) and steric interactions (red dotted lines) between selected ligands and the amino acid residues from the binding site of 1XKK receptor: compound **1a** ((**a**)—3D structure, (**b**)—2D structure), compound **1e** ((**c**)—3D structure, (**d**)—2D structure).

Derivatives **1a** and **1e** yielded the highest docking scores (−130.68 and −128.01 kcal/mol, respectively), sharing similar binding modes (Figure 11, Table S9). The keto group of quinazolin-4(3*H*)-one is engaged in steric interactions with Asp855, while the methyl substituent at position 3 interacts with Thr790. The *o*-hydroxyl group on the phenyl ring formed hydrogen bonds with Gln791. Additionally, compound **1a** interacted with Ala 743 via its imine carbon, with Lys745 through the hydrazone bond, and with Met793 via the phenyl ring (Figure 11b). Compound **1e** exhibited steric interactions with Thr854 and Leu777 via the quinazolin-4(3*H*)-one core (Figure 11c).

The compounds **1b**–**1d** adopted different orientations in the binding pocket, resulting in lower docking scores (Table S9, Figure S31). Compound **1b** interacted with Asp855 and Lys745 via the *o*-hydroxyl of the phenyl ring and with Thr790 via the keto group of the quinazolin-4(3*H*)-one core. Compound **1c** formed hydrogen bonds with Asp855 and Thr 854 via the *o*-hydroxyl of the phenyl ring, while compound **1d** interacted with the same amino acids using the quinazolin-4(3*H*)-one core.

## 3. Discussion

**Figure 12:**
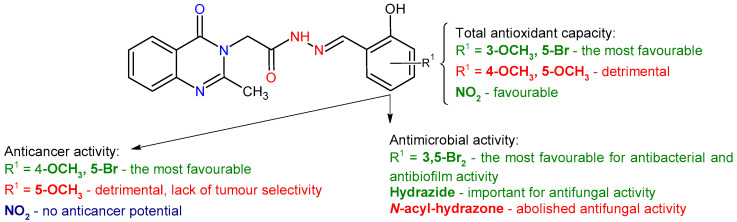
The correlations between the chemical structure of *N*-acyl-hydrazones **1a**–**1f** and their biological activities.

Correlations between structural features and observed biological activities can be identified, providing relevant insights into structure–activity relationships (Figure 12).

Antioxidant capacity of the methoxy-salicylaldehyde derivatives (**1c**–**1e**) depends on the position of the methoxy group on the phenyl ring, being maximum in *ortho* position relative to the hydroxyl group (**1c**—TAC 45.1%). This may be explained by the fact that the *ortho*-methoxy group stabilizes the oxygen radical by conjugation [31], which is not possible in the case of *meta* and *para* positions. Changing the *para*-methoxy group with an electron-withdrawing bromine increased antioxidant capacity (**1a**—TAC 42.7% compared with **1d**—TAC 19.3%). Introducing a second bromine atom in *ortho* position to the hydroxyl group did not lead to a further increase in activity (**1b**—TAC 38.9%). Additionally, nitro groups are beneficial for antioxidant capacity (**1f**—TAC 43.4%) (Figure 12).

The assessment of the antimicrobial activity of the synthesized compounds indicated selective efficacy, notably against Gram-positive bacteria and yeasts, with *S. aureus* and *C. albicans* being the most susceptible species. Compound **1b** exhibited both qualitative and quantitative antimicrobial activity against *S. aureus* (IZD: 12.33 ± 0.58 mm; MIC: 0.3125 mg/mL; IC_50_: 0.0857 mg/mL). This highlights the importance of dibromo substitution, as the corresponding monobromo (**1a**) and methoxy derivatives (**1c**–**1e**) exhibited markedly reduced or no antibacterial activity (Figure 12). These findings align with previous studies emphasizing the influence of dihalogen substitution on the activity of salicylaldehyde-derived hydrazones and imines [26,28,67,68].

Regarding the effect on *C. albicans*, 2-(2-methyl-4-oxoquinazolin-3(4*H*)-yl)acetohydrazide (**2**) demonstrated superior antifungal activity, surpassing both *N*-acyl-hydrazone derivatives and the DMSO control, whereas compound **1c** exhibited minimal activity (Figure 12). Compounds **1a** and **1c** reduced *C. albicans* adherence on inert substrate; additionally, compound **1a** inhibited *P. aeruginosa* adherence, both pathogens commonly associated with chronic biofilm infections.

Bromine-containing derivatives **1a** and **1b** also showed higher LogP values, consistent with increased hydrophobicity. Compounds with intermediate −LogS values (e.g., **1c**, −LogS = −3.59) displayed antibacterial effects, indicating that a minimal level of aqueous solubility is required, particularly for activity against *E. coli*. These findings underscore the importance of balancing lipophilicity and solubility when developing antibacterial.

Compounds **1a**–**1d** consistently showed cytotoxic activity against both HT-29 and A431 cancer cell lines. In contrast, these four derivatives indicated limited or no toxicity in the Vero cell line at concentrations below 50 µM. Notably, dibromo-substituted and 4-methoxy-substituted derivatives (**1b** and **1d**) even increased Vero cell viability at lower doses, supporting their selective activity toward cancer cells (Figure 12). On the other hand, the 5-methoxy-substituted derivative **1e** exhibited an opposite profile: its effects on cancer cells were reduced, whereas it induced marked cytotoxicity in Vero cells at ≥12.5 µM, indicating poor selectivity and raising concerns about off-target toxicity.

Compounds **1f** and **2** did not reduce the viability of the tested cell lines; instead, they slightly increased cell viability, suggesting excellent safety but no anticancer relevance. The reference drug 5-FU showed a dose-dependent reduction in cancer cell viability, although its effect within the tested concentration range was weaker than that of several *N*-acyl hydrazone derivatives. These findings suggest that specific chemical modifications have a notable influence on antimicrobial efficacy.

As a preliminary toxicity screening, the *Artemia* test provides significant predictive value due to its correlation with broader biological systems. While it does not replace mammalian models, it effectively detects general cytotoxic effects and acute hazards, helping to prioritize compounds for more specific tests such as liver or DNA toxicity. In this study, the *N*-acyl-hydrazones **1a**–**1f** exhibited lower overall toxicity, their lethality not exceeding 40% at 48 h of exposure.

In correlation with the in vitro results, the docking study supports some of the observed effects. Gyrases are prokaryotic type II DNA topoisomerases, which catalyse the supercoiling of bacterial DNA [69]. They are involved in transient breakage of both DNA strands during replication and in introducing negative supercoils via ATP hydrolysis (a difference from eubacterial topoisomerase IV) [70,71,72,73]. They consist of two subunits: GyrA, which interacts with DNA, and GyrB, the ATPase [74,75]. The second subunit is the target of aminocoumarin antibiotics, such as novobiocin, which act as competitive inhibitors at the ATP-binding site [63,74].

The predicted binding mode of derivative **1b** to *S. aureus* gyrase B aligns with previous proposed orientations for quinazoline-based inhibitors. In the series of *N*-(4-oxo-quinazolin-3(4*H*)-yl)-1-ethyl-4-hydroxy-2-oxo-1,2-dihydroquinoline-3-carboxamides developed by Xue et al., the 4-oxoquinazoline fragment is of utmost importance for gyrase B inhibition. It occupies the hydrophobic pocket surrounded by Asn54 and Ile86 and interacts with Thr173, Asp81, and Gly85 via a water bridge [54,55]. The same orientation of the quinazolinone fragment was observed for compound **1b**, with the mention that no water was included in the study. On the other hand, the hydrazide and the phenyl ring occupy the hydrophilic pocket, interacting with Arg84 and Glu58, similarly to the dihydroquinoline-3-carboxamide of the above-mentioned inhibitors [54,55].

In the case of lanosterol 14α-demethylase, the active site contains a heme residue, which is the key element that drives the oxidative removal of the 14α-methyl group of the sterol core [57,59]. All azole antifungals act by coordinating the iron ion of the heme core through their diazole and triazole rings [59] and competing with lanosterol for the space inside the enzyme’s active site [65]. Compared with the reference azole antifungals, the *N*-acyl-hydrazone **1b** occupies the middle portion of the binding site. It forms hydrogen bonds with Tyr132 and Tyr118, two of the aminoacids that anchor the prosthetic heme [65], the keto group of the quinazolinone ring being essential for these interactions.

Regarding the EGFR kinase, it is part of the Erb receptor tyrosine kinase family, also known as HER1 or Erb1. It has a role in cell survival, proliferation and inhibition of apoptosis, representing a key drug target in cancer therapy [24]. The active site of the tyrosine kinase may be divided into two regions: the ATP binding site, which contains the adenine binding pocket, a solvent-accessible region, and the hinge region, and the non-ATP region, reached by inhibitors, formed by two hydrophobic pockets and the K pocket [24,60].

In the case of present *N*-acyl-hydrazones, the substitution on the phenyl ring had a significant influence on the binding mode. In the case of 2,3 and 2,4-substituted derivatives **1c** and **1d**, the quinazolin-4(3*H*)-one core occupies the adenine pocket, and the phenyl ring lies into the K pocket (Figure S31e–h). Literature data support this alignment. In the series of *N*′-benzylidene-2-(6,8-dibromo-2-methyl-4-oxoquinazolin-3(4*H*)-yl)acetohydrazides developed by El-Mahdy et al., the hydrazone linker occupied the deep K pocket of the EGFR kinase active site and positioned the terminal phenyl ring in the second binding pocket [24].

In the case of 2,5-substituted derivatives **1a** and **1e**, the orientation is quite the opposite. The quinazolin-4(3*H*)-one core occupies the K pocket, interacting with Asp855 of the DFG motif, while the phenyl ring lies into the adenine pocket, forming hydrogen bonds with Gln791. The dibromo derivative **1b** makes an exception, because the quinazolin-4(3*H*)-one ring is directed into the hydrophobic pocket II, while the phenyl ring occupies the K pocket (Figure S31c,d).

## 4. Materials and Methods

### 4.1. Chemistry and Spectral Data

#### 4.1.1. General Information

All reactions were performed under microwave irradiation using a Biotage^®^ Initiator Classic 2.0 (Biotage, Uppsala, Sweden), in sealed 2–5 mL reaction vials, under magnetic stirring and high absorbance level. The reagents and the solvents were purchased from: Sigma-Aldrich, St. Louis, MO, USA (5-bromosalicylaldehyde, 3,5-dibromosalicylaldehyde); Merck Schuchardt, Hohenbrunn, Germany (2-hydroxy-3-methoxybenzaldehyde, 2-hydroxy-4-methoxybenzaldehyde, 2-hydroxy-5-methoxybenzaldehyde, 4-hydroxy-3-methoxy-5-nitrobenzaldehyde); Chemical Company S.A., Iasi, Romania (ethanol) and Chimopar Tranding SRL, Bucharest, Romania (glacial acetic acid).

For thin-layer chromatography (TLC), glass TLC plates coated with silica gel 60 F_254_, 5 cm × 10 cm (Metria) were used. The mobile phase consisted of a mixture of chloroform–methanol (9:1, *v*/*v*) (migration distance of 7 cm). The spots were visualized under UV light, at 254 nm. The reference substance was 2-(2-methyl-4-oxoquinazolin-3(4*H*)-yl)acetohydrazide (**2**).

The infrared spectra were recorded on JASCO FT/IR-4200 spectrometer ATR PRO 450S (diamond crystal). Absorption maxima are reported in wave numbers (cm^−1^), using the range 400–4000 cm^−1^ and transmittance is recorded on the abscissa. The following abbreviations were used: w (weak), m (medium), s (strong), and vs (very strong).

NMR spectra were recorded on Varian Mercury Plus 300 MHz spectrometer equipped with Probe 300Auto Sw PFG4 NUC 30–122 MHz operating at 300 MHz for ^1^H-NMR spectra and at 75 MHz for and ^13^C-NMR spectra. The compounds were dissolved in deuterated dimethyl sulfoxide, DMSO-d_6_, and tetramethylsilane, TMS, was used as internal standard. The chemical shifts are reported in parts per million (ppm, δ scale) relative to TMS and all coupling constant (J) values are in Hertz (Hz). To explain the multiplicities of proton signals, the following abbreviations were used: s (singlet), d (doublet), t (triplet), m (multiplet), dd (doublet of doublets), bd (broad doublet) and td (triplet of doublets). For ^1^H-NMR spectra, the data order is as follows: chemical shifts, multiplicity, coupling constants, number of protons and signal attribution; for ^13^C-NMR spectra, chemical shifts and signal attribution are reported. The subscripts M and m indicate major and minor signals.

Uncorrected melting points (m.p.) were determined using Electrothermal 9100 apparatus (Bibby Scientific Ltd., Stone, UK) by open capillary method.

The APCI+ high resolution mass spectra for compounds **1a**–**1f** and **2** were recorded on a Thermo Scientific LTQ-Orbitrap XL spectrometer equipped with a standard ESI/APCI source. Thermo Xcalibur software (version 4.0.27.19) was used for the processing of the mass spectra (XcaliburTM Software, Thermo Fisher Scientific, 168 3rd Avenue, Waltham, MA, USA, www.thermofisher.com, accessed on 15 March 2025).

#### 4.1.2. Synthesis and Spectral Characterization

The 2–5 mL reaction vials were loaded with a magnetic stirrer, 1 mmol of each reactant, acetohydrazide **2** and benzaldehydes, 4–4.5 mL of anhydrous ethanol and 2 drops of glacial acetic acid. Reaction conditions included 5 min of pre-stirring, followed by 40 min of irradiation at 100 °C, with the absorbance level set to “high”. The vials were left overnight in a refrigerator. The precipitates formed were filtered under vacuum, washed with cold ethanol and dried at room temperature. Thin-layer chromatography was performed to analyze and monitor the reaction. The compounds were confirmed by spectroscopic methods (FT-IR, ^1^H-NMR and ^13^C-NMR spectroscopy and mass spectrometry) and characterized by melting points, solubility and retention factors (R_f_).

### 4.2. In Silico ADMET Study and Prediction of Pharmacological Profile

Free web services were used to predict all physicochemical and pharmacological parameters: SwissADME [35]—druglikeness, leadlikeness, ADMET profile; pkCSM [76]—ADMET profile; SwissTargetPrediction [36], SuperPred database [37] and PassOnline [38]—pharmacological profile. To generate the predictions, SMILES of *E* isomers were obtained using ACD/ChemSketch (Freeware) 2020.1.2 (www.acdlabs.com) [77].

In the case of target selection, the following criteria were applied: the top 5 targets for each compound (SwissTargetPrediction) [78], targets with probability over 90% and with model accuracy over 85% (SuperPred) [37], activities with Pa greater than Pi and Pi under 0.2 (PassOnline). The graphs in Figure S26 were generated based on the selected targets.

### 4.3. Total Antioxidant Capacity (TAC)

In this study, the TAC values were obtained using the most-known DPPH method, in which the intense violet colour of the free radical solution is decreasing. Stock solutions of compounds **1a**–**1f** and **2** were prepared in absolute methanol at a concentration of 1 mg/mL. A stock solution of 2,2-diphenyl-1-picrylhydrazyl free radical was obtained in the same solvent, at a concentration of 2 × 10^−4^ M. To 1.8 mL of DPPH solution was added 0.2 mL of each solution of the compounds **1a**–**1f** and **2** and the mixture was left in the dark for 30 min, followed by measurements of the absorbance at 517 nm [79,80,81,82,83]. The following equation was used to calculate TAC:

$$TAC\left(\%\right)=\frac{{Abs}_{0\;min}-{Abs}_{30\;min}}{{Abs}_{0min}}\cdot 100$$

where Abs_0 min_ refers to the initial absorbance of the DPPH free radical solution, and Abs _30 min_ refers to the absorbance of the solution of DPPH and the added compound after 30 min. Ascorbic acid was used as a reference.

### 4.4. Antimicrobial Evaluation

#### 4.4.1. Microbial Strains

For the evaluation of antimicrobial activity, the following reference strains were used: *S. aureus* ATCC 25923, *E. faecalis* ATCC 29212, *E. coli* ATCC 25922, *P. aeruginosa* ATCC 27853 and *C. albicans* ATCC 10231.

#### 4.4.2. Qualitative Assessment of Antimicrobial Activity

The microbial suspensions were prepared at a density of 1.5 × 10^8^ CFU/mL, corresponding to the McFarland 0.5 nephelometric standard. These suspensions were obtained from 18–24 h cultures developed on solid media (Mueller-Hinton for bacteria, Sabouraud for yeast). Antimicrobial activity was assessed using an adapted Kirby-Bauer method according to CLSI (Clinical and Laboratory Standards Institute). In addition to the 10 mg/mL stock solutions prepared in DMSO for each compound, a solvent control was used. Each stock solution (10 μL) was distributed in spot on the medium inoculated with the interest strain. After measuring the diameter of the inhibition zone (IZD) around the spot (mm), the results were expressed as the mean ± standard deviation (SD) for each compound tested in duplicate.

#### 4.4.3. Quantitative Assessment of Antimicrobial Activity

Quantitative analysis was performed using the method of serial binary microdilutions in liquid medium (Tryptone Soy Broth for bacteria and Sabouraud Broth for yeasts) in plates with 96 wells. Stock solutions of 10 mg/mL were made in DMSO, and the range of concentrations tested for each compound was 5000–0.16 μg/mL. Simultaneously, the DMSO control was carried out under the same working conditions. Each well was inoculated with 10 μL of microbial suspension, adjusted to a density of 1.5 × 10^8^ CFU/mL prepared from 18 to 24 h cultures. After 24 h of incubation at 37 °C, microbial growth and multiplication were evaluated both macroscopically and spectrophotometrically at 620 nm to establish the MIC values. The data obtained were analyzed using the Inhibitor vs Response—Variable Slope (four parameters) analysis function in GraphPad Prism 10.0 software to calculate the IC_50_ (concentration of the sample that inhibits the growth of 50% of the untreated microbial inoculum). Blanks were made for each sample concentration, and the formula was applied:

$$Microbial\;viability(\%)=\frac{A_{sample}-A_{blank}}{A_{strain\;control}-A_{sterility\;control}}\cdot 100$$

A_sample_ = the absorbance of the inoculated sample

A_blank_ = the absorbance of the blank sample

A_strain control_ = the absorbance of the strain control

A_sterility control_ = the absorbance of the sterility control

The MIC value was considered to correspond to the lowest concentration at which a viability < 10% was observed.

#### 4.4.4. Microbicidal Activity Assessment

To determine the MMCs, 5 μL of culture was taken from each well and distributed on solid medium. The plates were incubated for 20–24 h at 37 °C. The lowest concentration at which no microbial growth was observed was considered MMC.

#### 4.4.5. Microbial Adhesion

Following quantitative analysis of antimicrobial activity, microbial adhesion was evaluated after methanol fixation and violet crystal staining (0.1%). The absorbance of the adhered biomass colored with violet crystal and resuspended in 33% acetic acid was measured at 490 nm.

#### 4.4.6. Statistical Analysis

Data are expressed as means ± standard deviation (SD) (n = 2). Statistical analysis was performed using GraphPad Prism 10.0. To compare the IC_50_ values of the newly synthesized compounds and solvent, one-way ANOVA was applied, followed by the Tukey multiple comparison test with a single combined variance. The significance level has been set to *p* < 0.05. The physicochemical data used in Figure 4 were calculated using ADMETlab3.0 [80].

### 4.5. Cytotoxicity Evaluation Against Normal and Cancer Cell Lines

#### 4.5.1. Cell Lines

For the cytotoxicity screening of the *N*-acyl-hydrazone derivatives, three cell lines were employed: HT-29 (human colorectal adenocarcinoma), A431 (human epidermoid carcinoma), and VERO (non-tumoral African green monkey kidney epithelial cells). All cell lines were obtained from the American Type Culture Collection (ATCC, Manassas, VA, USA), and maintained in appropriate culture media as follows: HT-29 and A431 cells were cultured in Dulbecco’s Modified Eagle Medium (DMEM), while VERO cells were cultured in Minimum Essential Medium (MEM). Both culture media were supplemented with 10% fetal bovine serum (FBS) and 1% penicillin-streptomycin mix, to formulate the complete culture medium. All cell lines were incubated at 37 °C in a humidified atmosphere containing 5% CO_2_. Media were refreshed every 2–3 days, and cells were subcultured upon reaching ~80% confluence using 0.25% trypsin-EDTA.

#### 4.5.2. Cytotoxicity Screening

For the cytotoxicity screening, cells were seeded into sterile 96-well plates at an initial density of 1 × 10^4^ cells/well and allowed to adhere for 24 h. Subsequently, cells were treated with increasing concentrations (3.125 to 50 µM) of compounds **1a**–**1f** and **2** for 24 h. In parallel, 5-fluorouracil (5-FU, Sigma Aldrich, St. Louis, MO, USA) was included as a reference drug and tested over the same concentration range under identical experimental conditions. For treatment, stock solutions of all tested compounds and 5-FU were freshly prepared in dimethyl sulfoxide (DMSO), stock solutions being diluted in complete culture medium to final concentrations of 50, 25, 12.5, 6.25, and 3.125 µM, ensuring that the final DMSO concentration did not exceed 0.1% (*v*/*v*) in any treatment condition. For the experimental control, culture medium was refreshed at the time of treatment application.

#### 4.5.3. Cell Viability

After 24 h of treatment, cell viability was assessed using the MTT assay, which employed 3-[4,5-dimethylthiazol-2-yl]- 2,5-diphenyltetrazolium bromide (Sigma-Aldrich, St. Louis, MO, USA). Briefly, the culture media were discarded from each well and replaced with a fresh solution of MTT (1 mg/mL). Plates were incubated for 4 h at 37 °C, and the resulting formazan crystals were solubilized with isopropanol. The absorbance was measured at 550 nm using the FlexStation III multimodal reader (Molecular Devices, San Jose, CA, USA).

#### 4.5.4. Statistical Analysis

All experiments were performed in triplicate and repeated in three independent assays. Cell viability was calculated as a percentage relative to the untreated control using the formula Cell Viability (%) = (Abs treated/Abs control) × 100. Data were processed using GraphPad Prism 6.0 and presented as mean ± standard deviation (SD). Statistical analysis was performed using one-way analysis of variance (ANOVA) followed by Dunnett’s post hoc test for comparison with the untreated control group. A *p*-value < 0.05 was considered statistically significant.

### 4.6. Artemia franciscana Toxicity Assay

The brine shrimp cysts (*Artemia franciscana*) were sourced from Great Salt Lake (USA) through Ocean Star International (Burlingame, CA, USA) via S.K. Trading (Bangkok, Thailand), which carried out the repackaging. Artificial seawater was prepared by dissolving a CoralMarine salt mixture (Grotech) in distilled water to achieve a concentration of about 30 g/L. The cysts were placed in artificial seawater at 25 °C under vigorous aeration. They were allowed to hatch 48 h before the onset of the test, the brine shrimp nauplii stage being selected for the toxicity assay.

The analyzed compounds were dissolved in 100 μL DMSO and then suspended in seawater to a final volume of 10 mL. The starting concentration was 1000 μM, followed by binary dilutions with seawater: 500 μM, 250 μM, 125 μM and 62.5 μM. A blank solution of 100 μL DMSO and seawater up to 10 mL was also prepared. 24 well-plates (6 × 4) were used and the assay was conducted in triplicate for each concentration and the blank solution. 15 ± 3 nauplii were added to each well, and the plates were placed into the dark. The viability of the nauplii was monitored at 24 and 48 h. Potassium dichromate (K_2_Cr_2_O_7_; Sigma-Aldrich) was used as a positive control, in concentrations ranging from 200 to 6.25 μg/mL (binary dilution).

The LC_50_ was estimated by interpolation using RStudio 2025.09.2 [84,85], the drc package [86]. Several non-linear regression models were applied: generalized log logistic regressions (LL.2, LL2.2, LL.4, LL.5) and Weibull functions (W1.2, W1.3, W2.2, W2.3). Based on the Akaike criterion, a four-parameter log-logistic model (LL.4) was selected as the best-fitting regression model for compound 2, whereas, for potassium dichromate, a Weibull function (W2.3) was indicated.

Ethical review and approval were not required for this toxicity assay using *Artemia franciscana*. This was confirmed exempt under institutional policy and in accordance with applicable legislation, because *Artemia* spp. are simple invertebrates not considered sentient. Within EU, Directive 2010/63/EU on the protection of animals used for scientific purposes is specifically limited (article 1(3)) to live non-human vertebrate animals and live cephalopods [87]. The Directive encourages the promotion of alternative methods to working with live vertebrate animals, such as studies on lower-sensitivity species, which we used in the study to determine the toxicity of new compounds.

### 4.7. Molecular Docking

The docking study was performed using Molegro Virtual Docker (2019) [88], and the structures of the compounds were prepared using Spartan’24 software [89]. All protein structures were downloaded from the Protein Data Bank (https://www.rcsb.org/, accessed on 8 October 2025).

The docking procedure was performed in accordance with the requirements specified by the software. The protocol can be outlined as follows: extraction of the co-crystallized ligand, identification of the binding site and binding pocket, re-docking of the ligand, and validation of the docking protocol. Hydrogen bonds between the co-crystallized ligand and amino acid residues were characterized, and the interaction network was established. Subsequently, novel ligands were incorporated into the docking system, and their interaction profiles and docking scores were computed. The co-crystallized ligands served as reference compounds for the comparative study.

## 5. Conclusions

In summary, seven *N*-acyl-hydrazone derivatives **1a**–**1f** were synthesized from 2-(2-methyl-4-oxoquinazolin-3(4*H*)-yl)acetohydrazide and various substituted benzaldehydes and salicylaldehydes under microwave irradiation.

The cytotoxicity assay revealed the selectivity of compounds **1a**–**1d** for cancer cell lines over normal cells, which supports further exploration. In addition, compound **1b** exhibited moderate antibacterial activity against *S. aureus*, indicating both anticancer and antimicrobial potential. In particular, greater lipophilicity appears to favor activity against Gram-positive bacteria by facilitating membrane permeation, whereas hydrosolubility is essential for activity against Gram-negative species, which have an outer membrane. The safety profile of the derivatives on Vero cell lines correlates with the low toxicity observed for *Artemia franciscana*.

While the present findings demonstrate that the synthesized compounds exhibit notable cytotoxic, antioxidant, and antimicrobial activities, the precise molecular mechanisms underlying these effects remain to be clarified. The docking study suggests some possible mechanisms, targeting of *S. aureus* gyrase B for antibacterial activity and EGFR kinase for the cytotoxic effect. The results support further exploration of the biological activity of this *N*-acyl-hydrazones series, particularly in terms of expanding the number of derivatives and gaining insight into the possible mechanism of action through molecular dynamics simulation and in vitro analysis.

## Figures and Tables

**Table 1:** The total antioxidant activity for compounds **1a**–**1f** and **2**.

Compound	TAC (%)
**1a**	42.7
**1b**	38.9
**1c**	45.1
**1d**	20.5
**1e**	19.3
**1f**	43.4
**2**	19.7

**Table 2:** Results of the qualitative and quantitative assessment of antimicrobial and antibiofilm activity. Notations: inhibition zone diameter (IZD), minimum inhibitory concentration (MIC), minimum microbicidal concentration (MMC), minimum inhibitory concentration for microbial adherence (MICMA). Highlighted in orange—MIC values that are lower than those of the solvent; highlighted in blue—MMC values that are lower than those of the solvent; highlighted in pink—MICMA values lower than the MIC values and the MICMA for DMSO. The more active compounds are indicated in bold.

Compound	Parameter	*S. aureus* ATCC 25923	*E. faecalis* ATCC 9212	*P. aeruginosa* ATCC 27853	*E. coli* ATCC 25922	*C. albicans* ATCC 10231
**1a**	IZD (mm)	0.00 ± 0.00	0.00 ± 0.00	0.00 ± 0.00	0.00 ± 0.00	0.00 ± 0.00
	MIC (mg/mL)	**1.25**	**1.25**	1.25	1.25	**0.625**
	MMC (mg/mL)	>5	5	2.5	2.5	5
	MICMA (mg/mL)	2.5	1.25	0.625	1.25	0.625
**1b**	IZD (mm)	**12.33 ± 0.58**	0.00 ± 0.00	0.00 ± 0.00	0.00 ± 0.00	0.00 ± 0.00
	MIC (mg/mL)	**0.3125**	>5	5	1.25	**0.625**
	MMC (mg/mL)	>5	>5	5	2.5	5
	MICMA (mg/mL)	1.25	5	1.25	2.5	5
**1c**	IZD (mm)	**7.67 ± 0.58**	**7.67 ± 0.58**	0.00 ± 0.00	0.00 ± 0.00	**6.67 ± 0.58**
	MIC (mg/mL)	2.5	2.5	1.25	1.25	2.5
	MMC (mg/mL)	5	>5	2.5	5	5
	MICMA (mg/mL)	2.5	2.5	1.25	2.5	0.625
**1d**	IZD (mm)	0.00 ± 0.00	0.00 ± 0.00	0.00 ± 0.00	0.00 ± 0.00	0.00 ± 0.00
	MIC (mg/mL)	2.5	5	1.25	1.25	5
	MMC (mg/mL)	>5	>5	1.25	5	>5
	MICMA (mg/mL)	2.5	5	1.25	2.5	1.25
**1e**	IZD (mm)	0.00 ± 0.00	0.00 ± 0.00	0.00 ± 0.00	0.00 ± 0.00	0.00 ± 0.00
	MIC (mg/mL)	**1.25**	5	5	5	1.25
	MMC (mg/mL)	5	>5	5	5	2.5
	MICMA (mg/mL)	2.5	5	1.25	5	2.5
**1f**	IZD (mm)	0.00 ± 0.00	0.00 ± 0.00	0.00 ± 0.00	0.00 ± 0.00	0.00 ± 0.00
	MIC (mg/mL)	2.5	**1.25**	1.25	1.25	1.25
	MMC (mg/mL)	5	>5	2.5	2.5	5
	MICMA (mg/mL)	2.5	2.5	1.25	2.5	1.25
**2**	IZD (mm)	0.00 ± 0.00	0.00 ± 0.00	0.00 ± 0.00	0.00 ± 0.00	**6.33 ± 1.53**
	MIC (mg/mL)	2.5	2.5	1.25	1.25	1.25
	MMC (mg/mL)	>5	>5	5	2.5	5
	MICMA (mg/mL)	2.5	>5	1.25	1.25	1.25
Ciprofloxacin	IZD (100 μg/mL, mm)	17.33 ± 0.58	8.33 ± 0.58	15.33 ± 0.58	34.00 ± 2.00	-
	MIC (μg/mL)	0.78	0.78	0.39	0.20	-
	MMC (μg/mL)	1.56	6.25	0.39	0.78	-
	MICMA (μg/mL)	0.39	0.78	0.39	<0.10	-
Ketoconazole	IZD (140 μg/mL, mm)	-	-	-	-	6.5 ± 0.71
	MIC (μg/mL)	-	-	-	-	8.75
	MMC (μg/mL)	-	-	-	-	35
	MICMA (μg/mL)	-	-	-	-	0.27
DMSO	IZD (mm)	0.00 ± 0.00	0.00 ± 0.00	0.00 ± 0.00	0.00 ± 0.00	0.00 ± 0.00
	MIC (mg/mL)	2.5	2.5	1.25	1.25	1.25
	MMC (mg/mL)	5	>5	2.5	5	5
	MICMA (mg/mL)	5	2.5	1.25	5	1.25

## Data Availability

The original contributions presented in this study are included in the article/Supplementary Materials. Further inquiries can be directed to the corresponding author.

## Supplementary Materials

The following supporting information can be downloaded at: https://www.mdpi.com/article/10.3390/ph19010057/s1. Figure S1. ^1^H-RMN spectrum of *N′*-[(*E*)-(5-bromo-2-hydroxyphenyl)methylidene]-2-(2-methyl-4-oxoquinazolin-3(4*H*)-yl)acetohydrazide (**1a**). Figure S2. ^13^C-RMN spectrum of *N′*-[(*E*)-(5-bromo-2-hydroxyphenyl)methylidene]-2-(2-methyl-4-oxoquinazolin-3(4*H*)-yl)acetohydrazide (**1a**). Figure S3. FT-IR spectrum of *N′*-[(*E*)-(5-bromo-2-hydroxyphenyl)methylidene]-2-(2-methyl-4-oxoquinazolin-3(4*H*)-yl)acetohydrazide (**1a**). Figure S4. APCI+ MS spectrum of **1a** in methanol. Experimental (up) and calculated (down) APCI+ MS spectra of **1a** in DMSO + MeOH. Figure S5. ^1^H-RMN spectrum of *N′*-[(*E*)-(3,5-bromo-2-hydroxyphenyl)methylidene]-2-(2-methyl-4-oxoquinazolin-3(4*H*)-yl)acetohydrazide (**1b**). Figure S6. ^13^C-RMN spectrum of *N′*-[(*E*)-(3,5-bromo-2-hydroxyphenyl)methylidene]-2-(2-methyl-4-oxoquinazolin-3(4*H*)-yl)acetohydrazide (**1b**). Figure S7. FT-IR spectrum of *N′*-[(*E*)-(3,5-bromo-2-hydroxyphenyl)methylidene]-2-(2-methyl-4-oxoquinazolin-3(4*H*)-yl)acetohydrazide (**1b**). Figure S8. APCI+ MS spectrum of **1b** in methanol. Experimental (up) and calculated (down) APCI+ MS spectra of **1b** in DMSO + MeOH. Figure S9. ^1^H-RMN spectrum of *N′*-[(E)-(2-hydroxy-3-methoxyphenyl)methylidene]-2-(2-methyl-4-oxoquinazolin-3(4H)-yl)acetohydrazide (**1c**). Figure S10. ^13^C-RMN spectrum of *N′*-[(*E*)-(2-hydroxy-3-methoxyphenyl)methylidene]-2-(2-methyl-4-oxoquinazolin-3(4*H*)-yl)acetohydrazide (**1c**). Figure S11. FT-IR spectrum of *N′*-[(*E*)-(2-hydroxy-3-methoxyphenyl)methylidene]-2-(2-methyl-4-oxoquinazolin-3(4*H*)-yl)acetohydrazide (**1c**). Figure S12. APCI+ MS spectrum of **1c** in methanol. Experimental (up) and calculated (down) APCI+ MS spectra of **1c** in DMSO + MeOH. Figure S13. ^1^H-RMN spectrum of *N′*-[(*E*)-(2-hydroxy-4-methoxyphenyl)methylidene]-2-(2-methyl-4-oxoquinazolin-3(4*H*)-yl)acetohydrazide (**1d**). Figure S14. ^13^C-RMN spectrum of *N′*-[(*E*)-(2-hydroxy-4-methoxyphenyl)methylidene]-2-(2-methyl-4-oxoquinazolin-3(4H)-yl)acetohydrazide (**1d**). Figure S15. FT-IR spectrum of *N′*-[(*E*)-(2-hydroxy-4-methoxyphenyl)methylidene]-2-(2-methyl-4-oxoquinazolin-3(4*H*)-yl)acetohydrazide (**1d**). Figure S16. APCI+ MS spectrum of **1d** in methanol. Experimental (up) and calculated (down) APCI+ MS spectra of **1d** in DMSO + MeOH. Figure S17. ^1^H-RMN spectrum of *N′*-[(*E*)-(2-hydroxy-5-methoxyphenyl)methylidene]-2-(2-methyl-4-oxoquinazolin-3(4*H*)-yl)acetohydrazide (**1e**). Figure S18. ^13^C-RMN spectrum of *N′*-[(*E*)-(2-hydroxy-5-methoxyphenyl)methylidene]-2-(2-methyl-4-oxoquinazolin-3(4*H*)-yl)acetohydrazide (**1e**). Figure S19. FT-IR spectrum of *N′*-[(*E*)-(2-hydroxy-5-methoxyphenyl)methylidene]-2-(2-methyl-4-oxoquinazolin-3(4*H*)-yl)acetohydrazide (**1e**). Figure S20. APCI+ MS spectrum of **1e** in methanol. Experimental (up) and calculated (down) APCI+ MS spectra of **1e** in DMSO + MeOH. Figure S21. ^1^H-RMN spectrum of *N′*-[(*E*)-(4-hydroxy-3-methoxy-5-nitrophenyl)methylidene]-2-(2-methyl-4-oxoquinazolin-3(4*H*)-yl)acetohydrazide (**1f**). Figure S22. ^13^C-RMN spectrum of N′-[(E)-(4-hydroxy-3-methoxy-5-nitrophenyl)methylidene]-2-(2-methyl-4-oxoquinazolin-3(4H)-yl)acetohydrazide (**1f**). Figure S23. FT-IR spectrum of *N′*-[(*E*)-(4-hydroxy-3-methoxy-5-nitrophenyl)methylidene]-2-(2-methyl-4-oxoquinazolin-3(4*H*)-yl)acetohydrazide (**1f**). Figure S24. Experimental (up) and calculated (down) APCI+ MS spectra of **1f** in DMSO + MeOH. Figure S25. LogP (a) and −LogS (b) predicted with different softwares (SwissADME, pkCSM) for compounds **1a**–**1f** and acetohydrazide 2. Figure S26. Top target predictions for the series of N-acyl-hydrazones **1a**–**1f** using SwissTargetPrediction (a) and SuperPred database (b). Figure S27. Dose-response curves for 2-(2-methyl-4-oxoquinazolin-3(4*H*)-yl)acetohydrazide (a) and potassium dichromate (b) in the lethality test on *Artemia franciscana*. Lethality is expressed as the mean lethality %. Figure S28. 4URO protein: Binding site (a) and binding pocket (b) of the co-crystallized novobiocin. Hydrogen bond (blue dotted lines) and steric interactions (red dotted) between novobiocin and amino acid residues from the binding site of 4URO (c—3D structure, d—2D structure). Figure S29. 2W9H protein: Binding site (a) and binding pocket (b) of the co-crystallized trimethoprim. Hydrogen bond (blue dotted lines) and steric interactions (red dotted) between trimethoprim and amino acid residues from the binding site of 2W9H (c—3D structure, d—2D structure). Figure S30. 6AYB protein: Binding site (a) and binding pocket (b) of the co-crystallized ligand KKK. Hydrogen bond (blue dotted lines) between ketoconazole and amino acid residues from the binding site of 6AYB receptor (c—3D structure, d—2D structure). Figure S31. Hydrogen bond (blue dotted lines) and steric interactions (red dotted) selected ligands and the amino acid residues from the binding site of 1XKK protein: lapatinib (a—3D structure, b—2D structure), compound **1b** (c—3D structure, d—2D structure), compound **1c** (e—3D structure, f—2D structure), compound **1d** (g—3D structure, h—2D structure). Table S1. Predicted absorption, distribution, metabolism, excretion and toxicity parameters for *N*-acyl-hydrazones **1a**–**1f**. Table S2. Top 5 protein targets for *N*-acyl-hydrazones **1a**–**1f** (predicted with SwissTargetPrediction). Table S3. Top 5 protein targets for N-acyl-hydrazones **1a**–**1f** (predicted with SwissTargetPrediction). Table S4. Target prediction, indications, probability and model accuracy for *N*-acyl-hydrazones **1a**–**1f** (predicted with SuperPRED). Table S5. Predicted activity for *N*-acyl-hydrazones **1a**–**1f** (Passonline simulations). Table S6. Docking scores, molecule contributions, binding interactions of selected ligands in the active site of 4URO protein. Table S7. Docking scores, molecule contributions, binding interactions of selected ligands in the active site of 2W9H protein. Table S8. Docking scores, molecule contributions, and binding interactions of selected ligands in the active site of 6AYB protein. Table S9. Docking scores, molecule contributions, and binding interactions of selected ligands in the active site of 1XKK protein.
