# Supplementary material for: Design, Synthesis, and Biological Evaluation of N-Acyl-Hydrazone-Linked Quinazolinone Derivatives with Antioxidant, Antimicrobial, and Anticancer Potential
Source: Pharmaceuticals (Basel). 2025 Dec 26;19(1):57. doi: 10.3390/ph19010057 (PMC12845261; doi:10.3390/ph19010057)
Supplement: Supplementary file 1 [file pharmaceuticals-19-00057-s001.zip › pharmaceuticals-4068945-supplementary.pdf]

Supplementary materials

Design, Synthesis, and Biological Evaluation of *N*-Acyl-Hydrazone-Linked Quinazolinone Derivatives with Antioxidant, Antimicrobial, and Anticancer Potential

Maria Coandă, Constantin Drăghici, Lucia Pintilie, Erzsébet-Eleonóra Kapronczai, Cornel Chiriță, Ioana-Cristina Marinaş, Robert-Viorel Ancuceanu, Irina Zarafu, Petru Ioniță, Denisa-Ioana Crăciun, Ariana Hudiță, Bianca Gălățeanu, Carmen Limban, Diana Camelia Nuță

Experimental Procedures

Spectral characterization of *N*-acyl-hydrazones 1a–1f

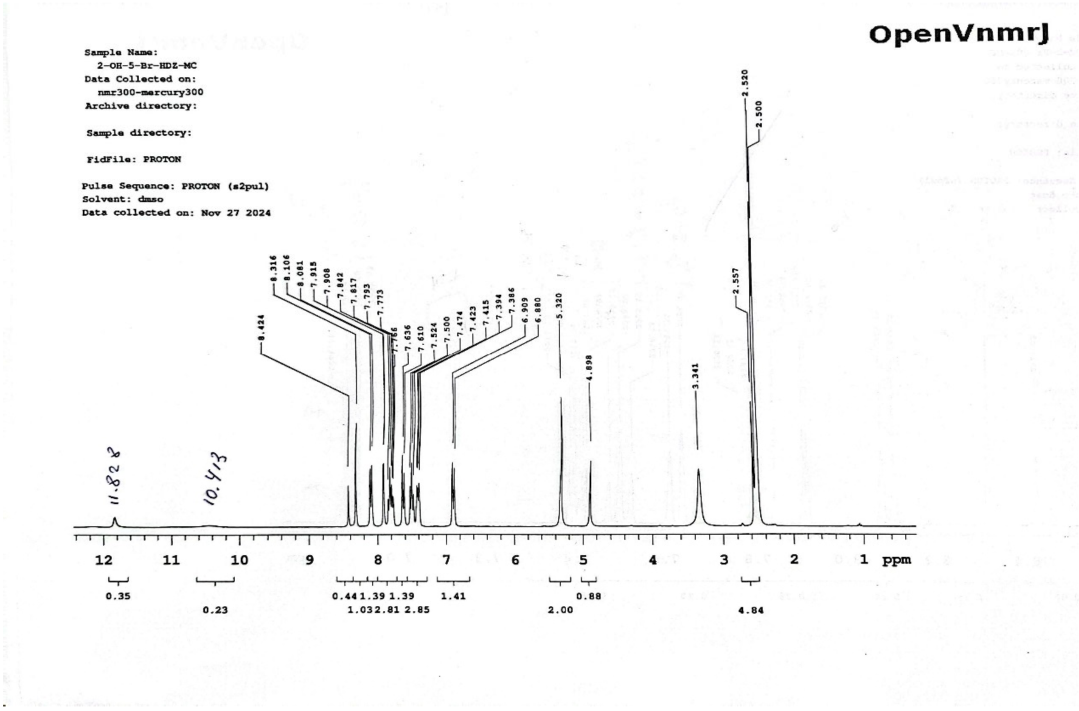

Figure S1. ^{1}H-RMN spectrum of N'-[(E)-(5-bromo-2-hydroxyphenyl)methylidene]-2-(2-methyl-4-oxoquinazolin-3(4H)-yl)acetohydrazide (1a).

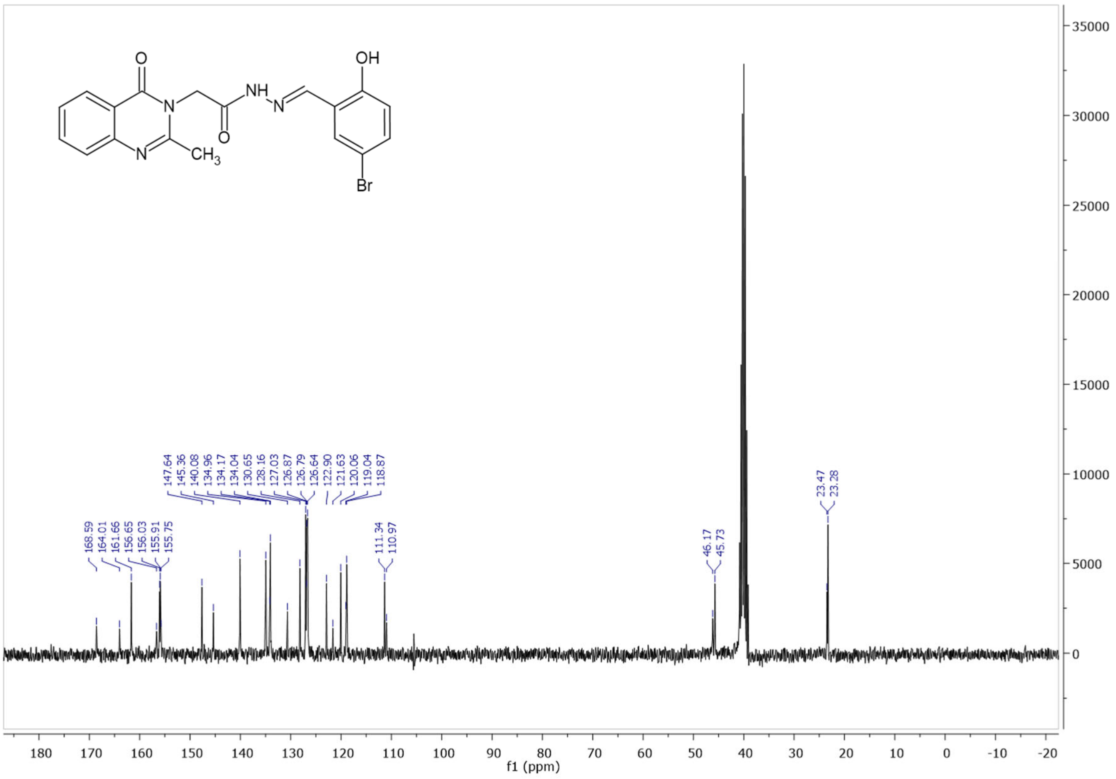

Figure S2. ^{13}C-RMN spectrum of N'-[(E)-(5-bromo-2-hydroxyphenyl)methylidene]-2-(2-methyl-4-oxoquinazolin-3(4H)-yl)acetohydrazide (1a).

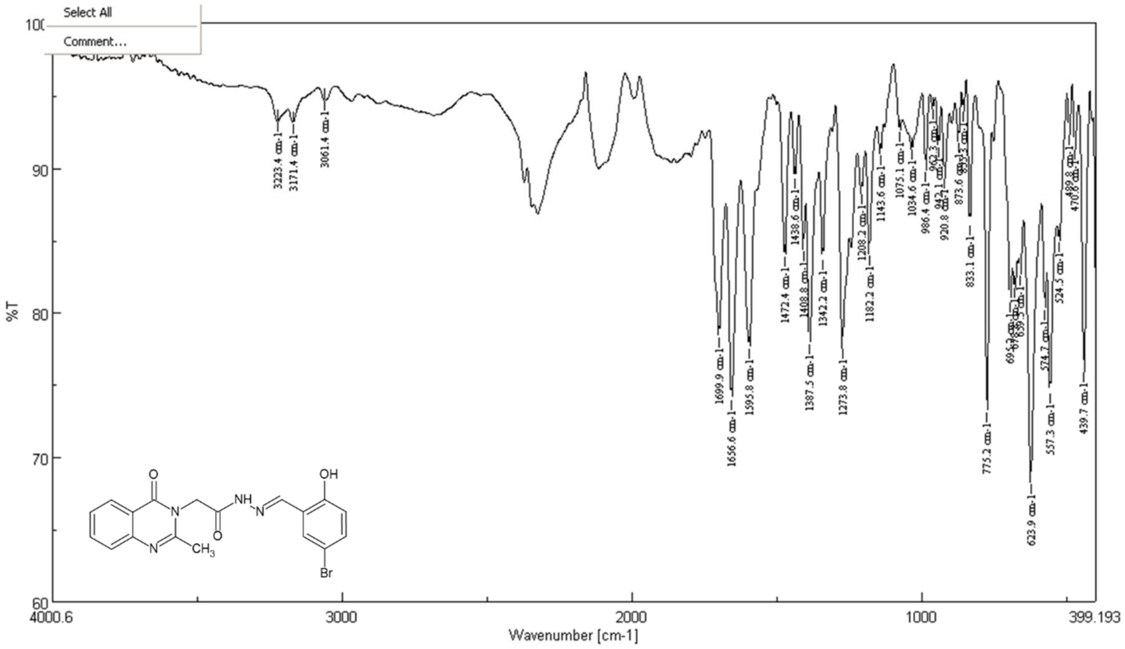

Figure S3. FT-IR spectrum of N'-[(E)-(5-bromo-2-hydroxyphenyl)methylidene]-2-(2-methyl-4-oxoquinazolin-3(4H)-yl)acetohydrazide (1a).

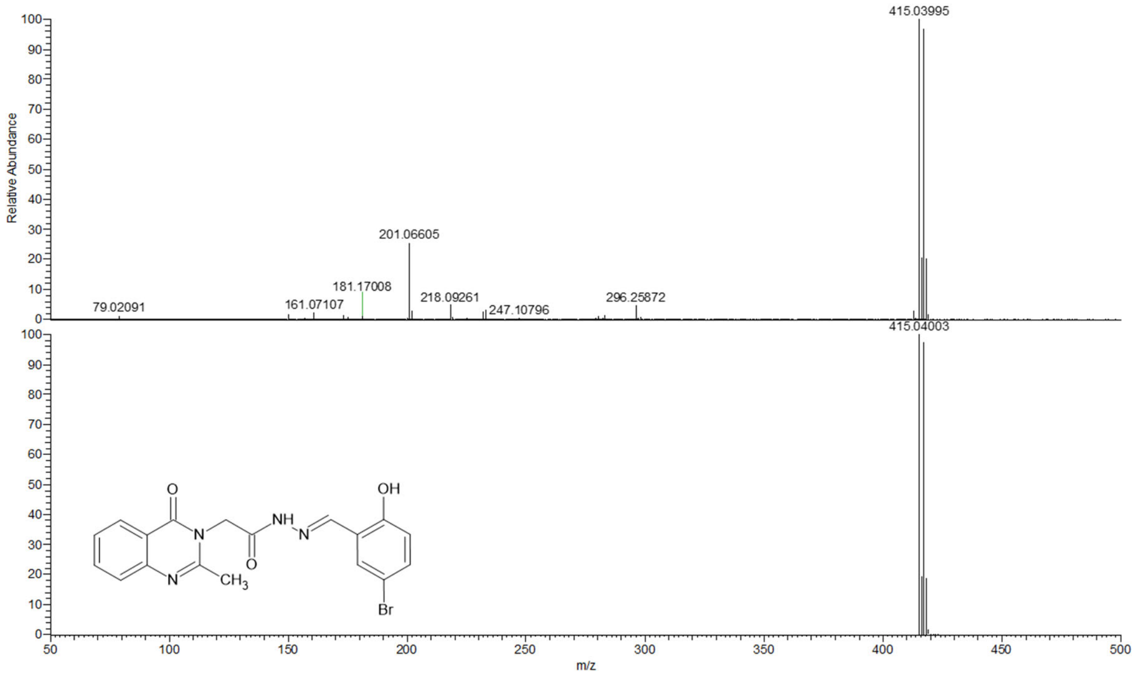

Figure S4. APCI+ MS spectrum of 1a in methanol. Experimental (up) and calculated (down) APCI+ MS spectra of 1a.

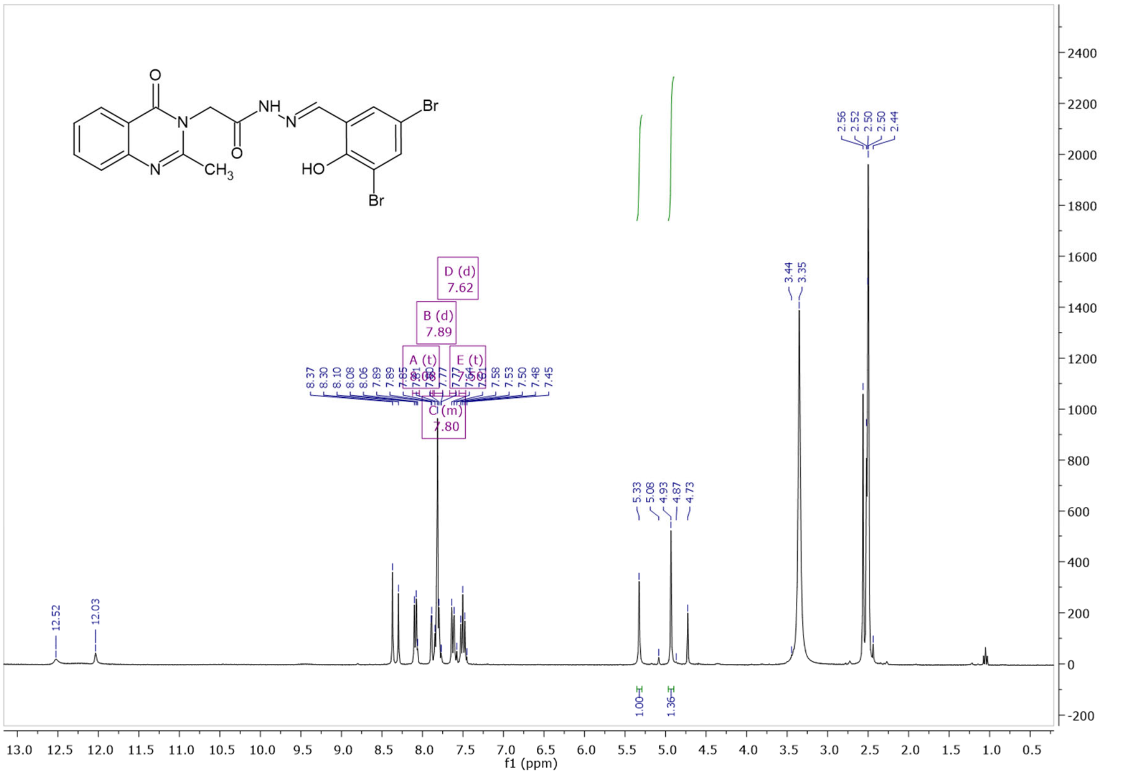

Figure S5. ^{1}H-RMN spectrum of N'-[(E)-(3,5-bromo-2-hydroxyphenyl)methylidene]-2-(2-methyl-4-oxoquinazolin-3(4H)-yl)acetohydrazide (1b).

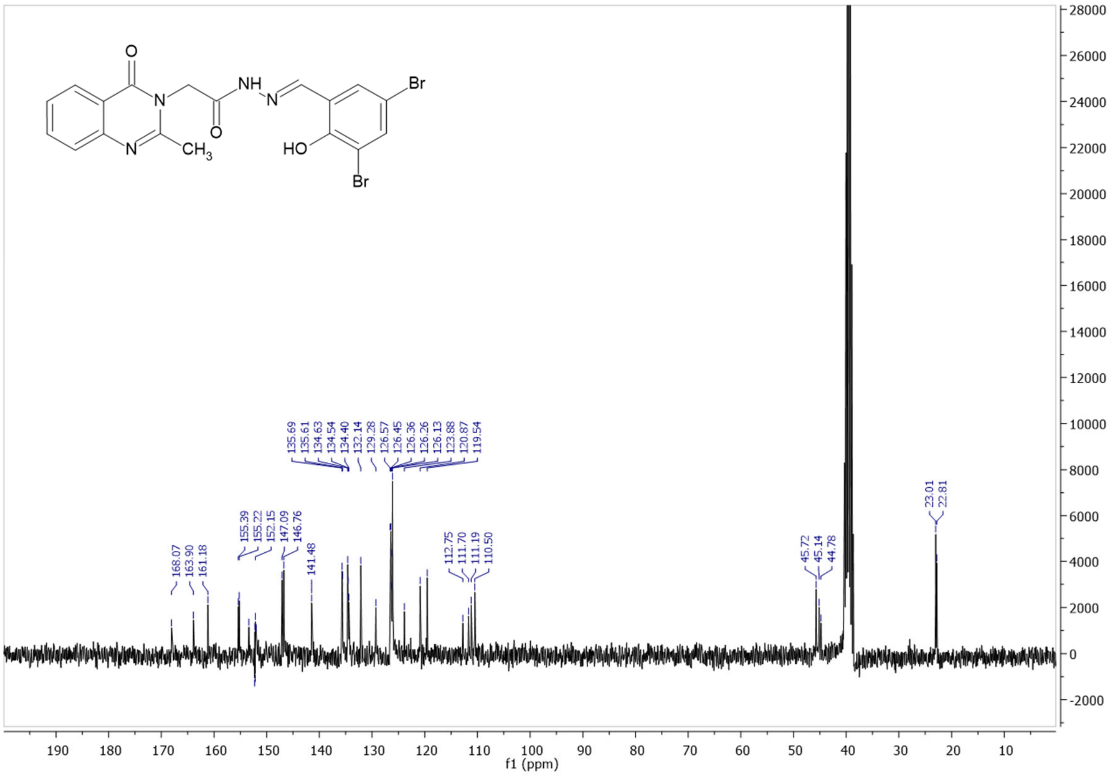

Figure S6. ^{13}C-RMN spectrum of N'-[(E)-(3,5-bromo-2-hydroxyphenyl)methylidene]-2-(2-methyl-4-oxoquinazolin-3(4H)-yl)acetohydrazide (1b).

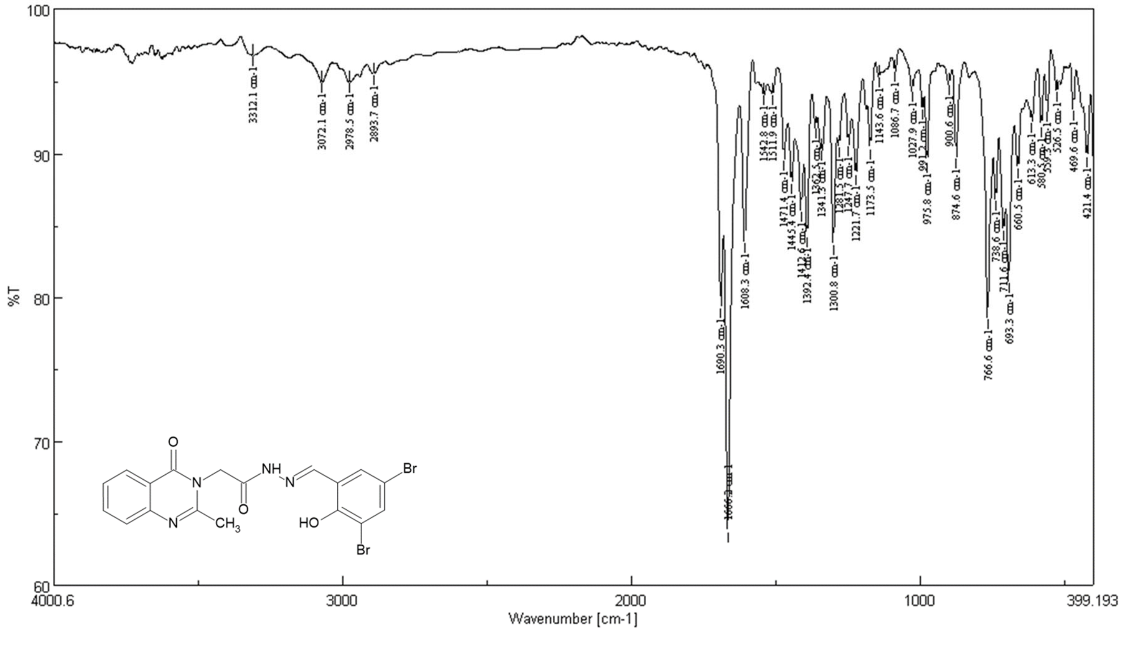

Figure S7. FT-IR spectrum of N'-[(E)-(3,5-bromo-2-hydroxyphenyl)methylidene]-2-(2-methyl-4-oxoquinazolin-3(4H)-yl)acetohydrazide (1b).

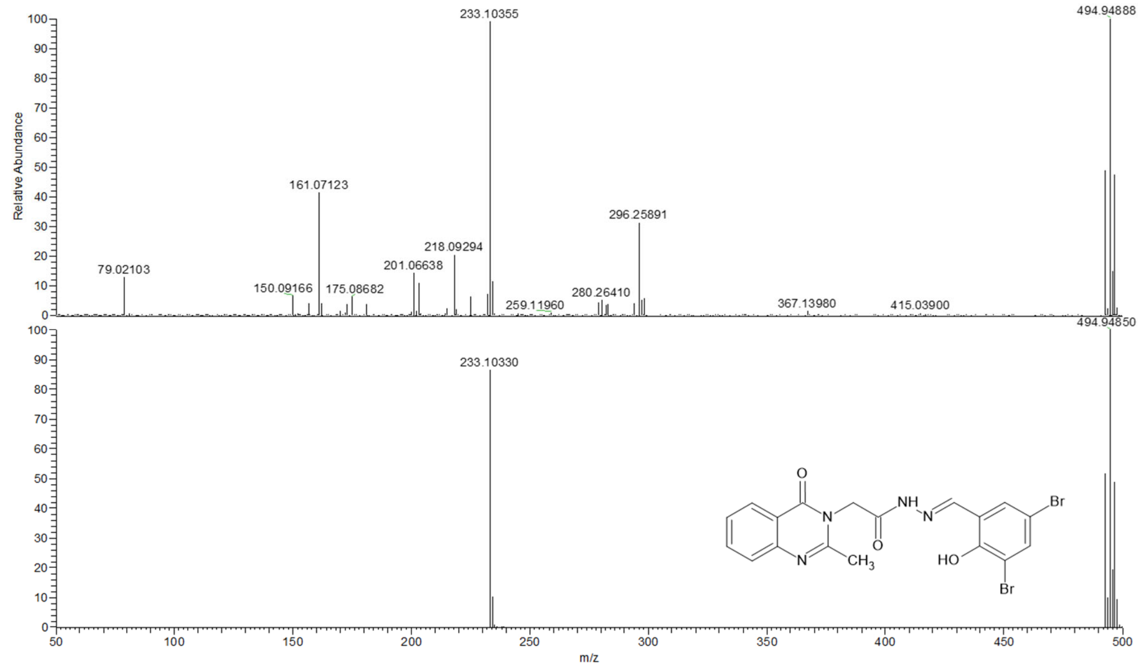

Figure S8. APCI+ MS spectrum of 1b in methanol. Experimental (up) and calculated (down) APCI+ MS spectra of 1b.

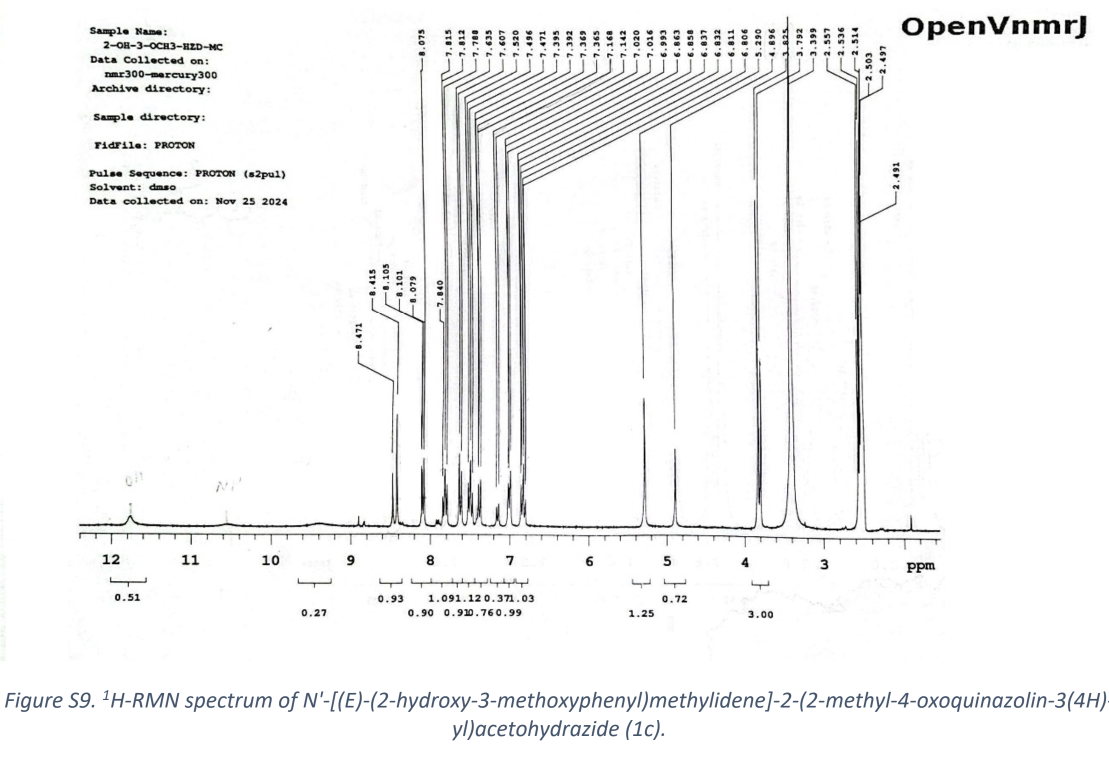

Figure S9. ¹H-RMN spectrum of N'-[(E)-(2-hydroxy-3-methoxyphenyl)methylidene]-2-(2-methyl-4-oxoquinazolin-3(4H)-yl)acetohydrazide (1c).

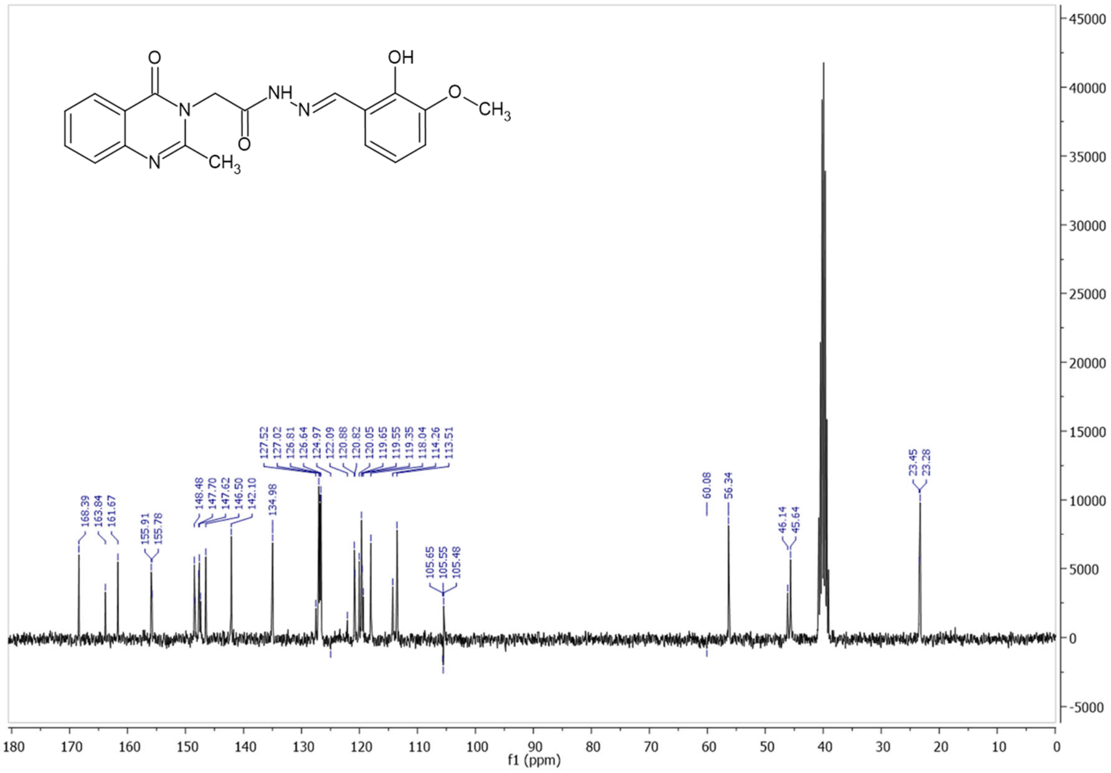

Figure S10. ^{13}C-RMN spectrum of N'-[(E)-(2-hydroxy-3-methoxyphenyl)methylidene]-2-(2-methyl-4-oxoquinazolin-3(4H)-yl)acetohydrazide (1c).

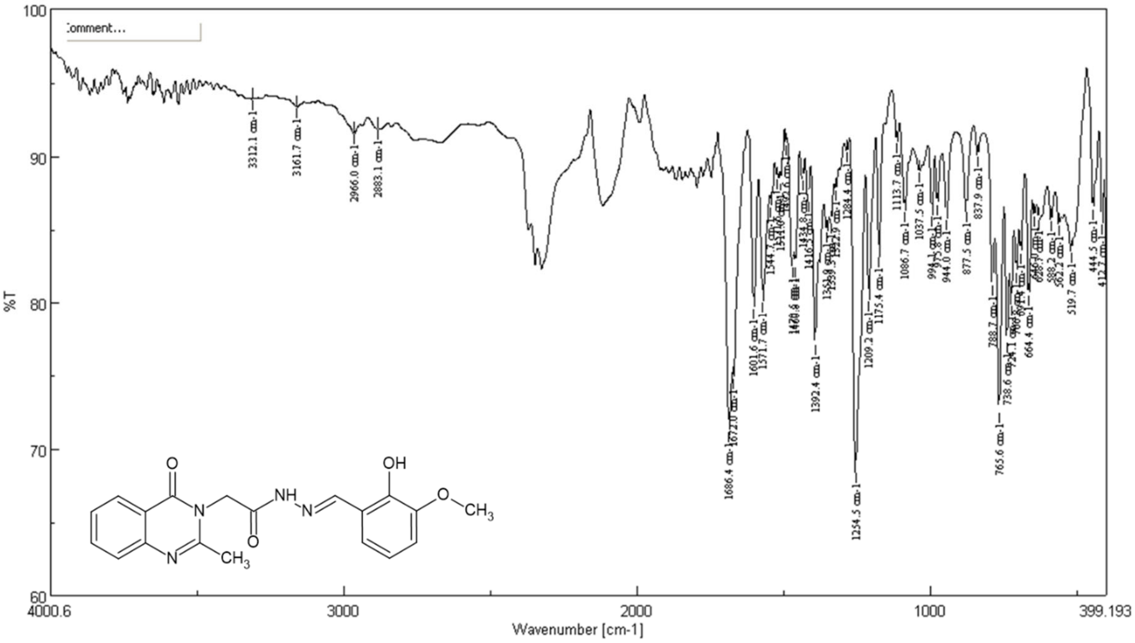

Figure S11. FT-IR spectrum of N'-[(E)-(2-hydroxy-3-methoxyphenyl)methylidene]-2-(2-methyl-4-oxoquinazolin-3(4H)-yl)acetohydrazide (1c).

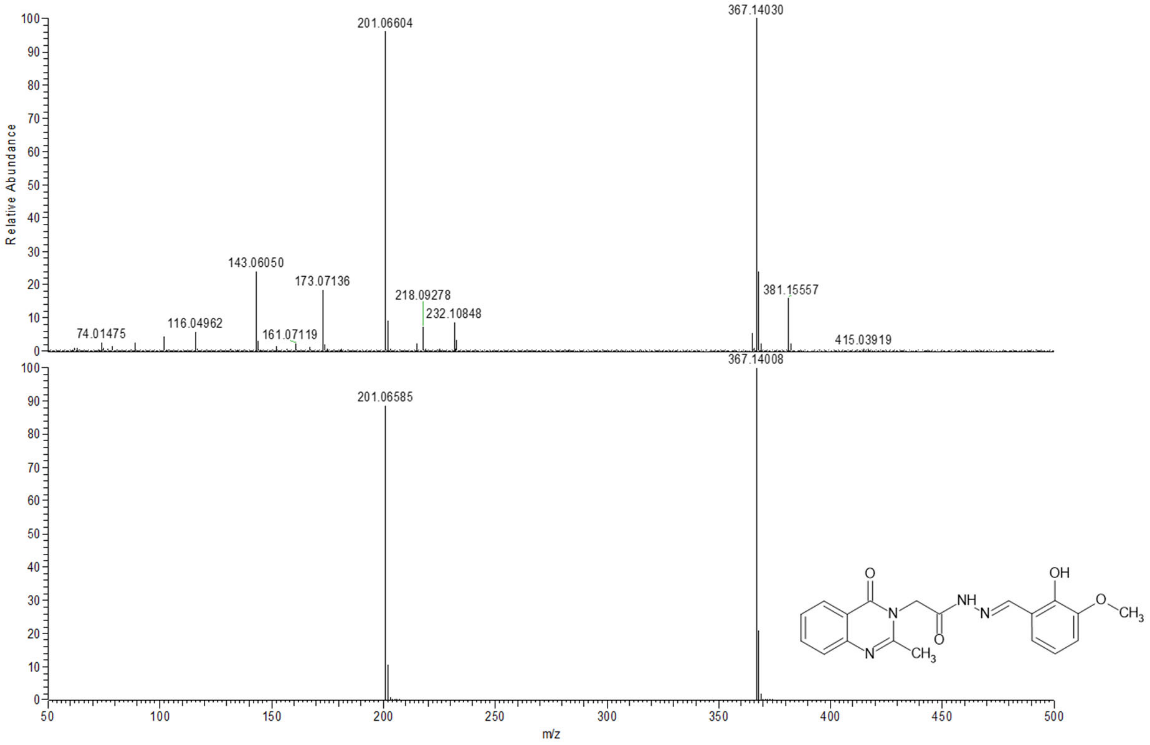

Figure S12. APCI+ MS spectrum of 1c in methanol. Experimental (up) and calculated (down) APCI+ MS spectra of 1c.

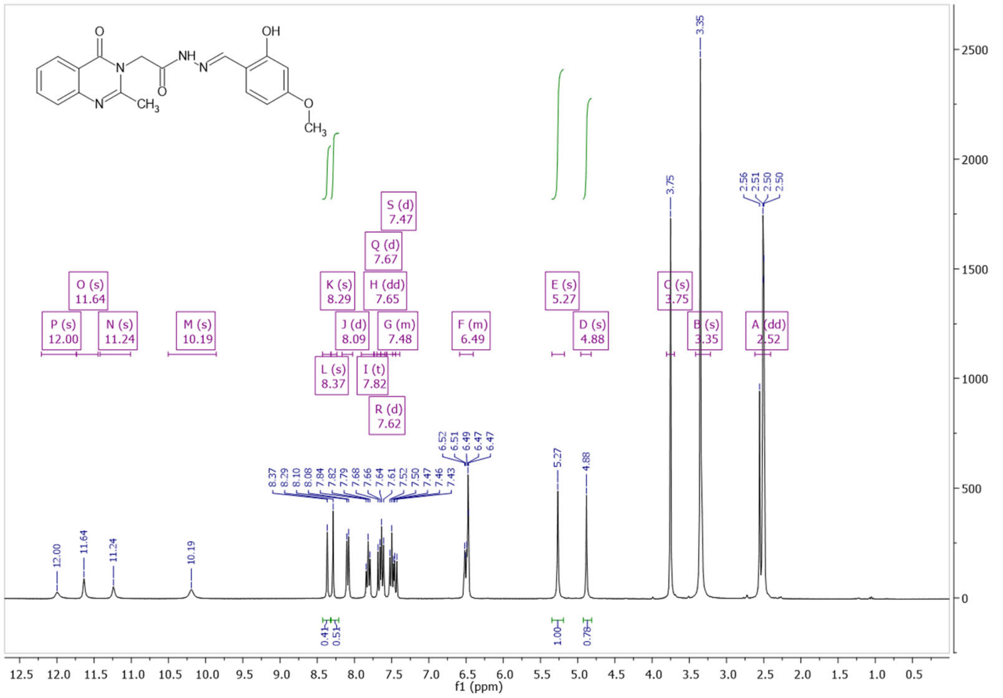

Figure S13. ^{1}H-RMN spectrum of N'-[(E)-(2-hydroxy-4-methoxyphenyl)methylidene]-2-(2-methyl-4-oxoquinazolin-3(4H)-yl)acetohydrazide (1d).

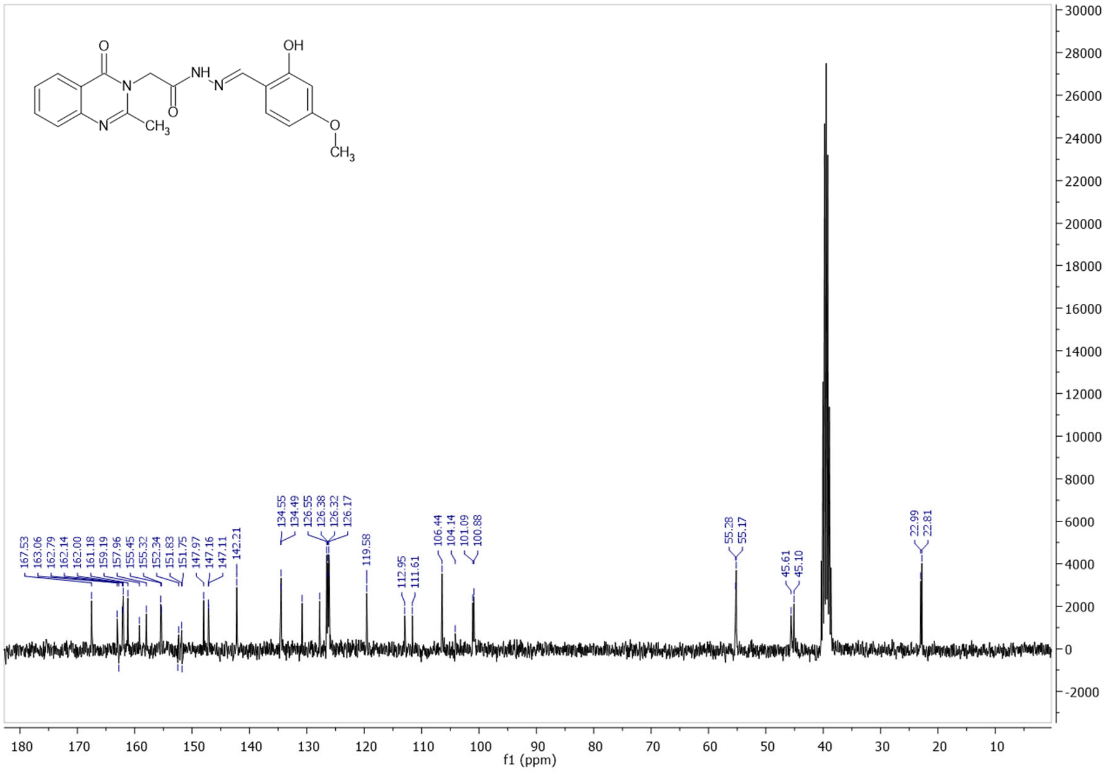

Figure S14. ^{13}C-RMN spectrum of N'-[(E)-(2-hydroxy-4-methoxyphenyl)methylidene]-2-(2-methyl-4-oxoquinazolin-3(4H)-yl)acetohydrazide (1d).

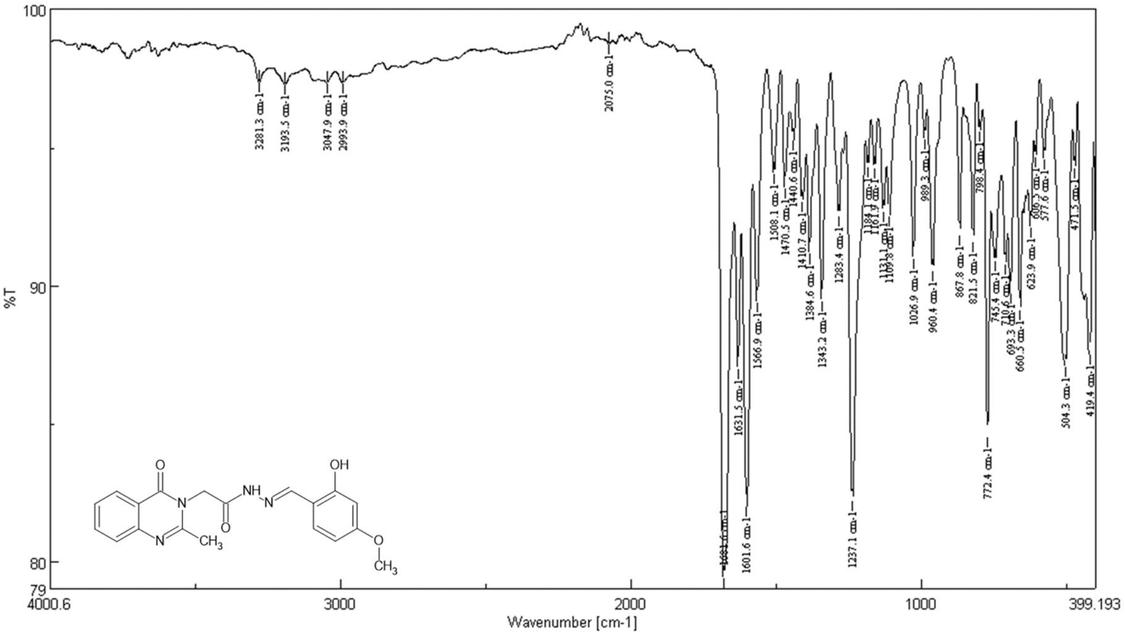

Figure S15. FT-IR spectrum of N'-[(E)-(2-hydroxy-4-methoxyphenyl)methylidene]-2-(2-methyl-4-oxoquinazolin-3(4H)-yl)acetohydrazide (1d).

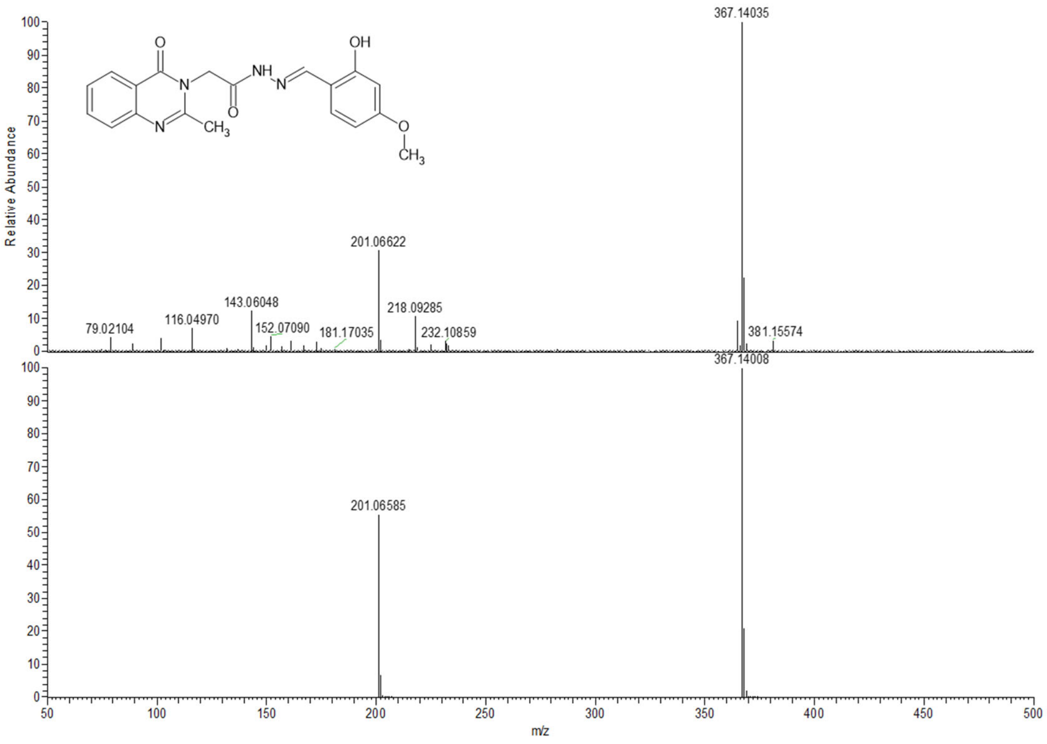

Figure S16. APCI+ MS spectrum of 1d in methanol. Experimental (up) and calculated (down) APCI+ MS spectra of 1d.

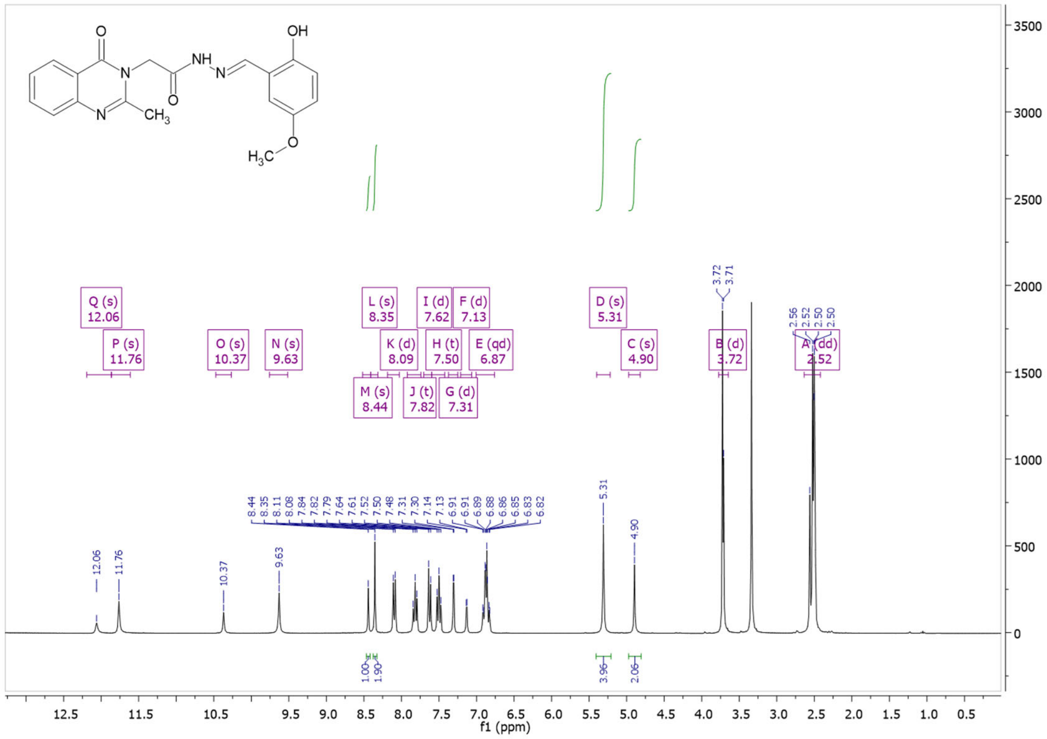

Figure S17. ^{1}H-RMN spectrum of N'-[(E)-(2-hydroxy-5-methoxyphenyl)methylidene]-2-(2-methyl-4-oxoquinazolin-3(4H)-yl)acetohydrazide (1e).

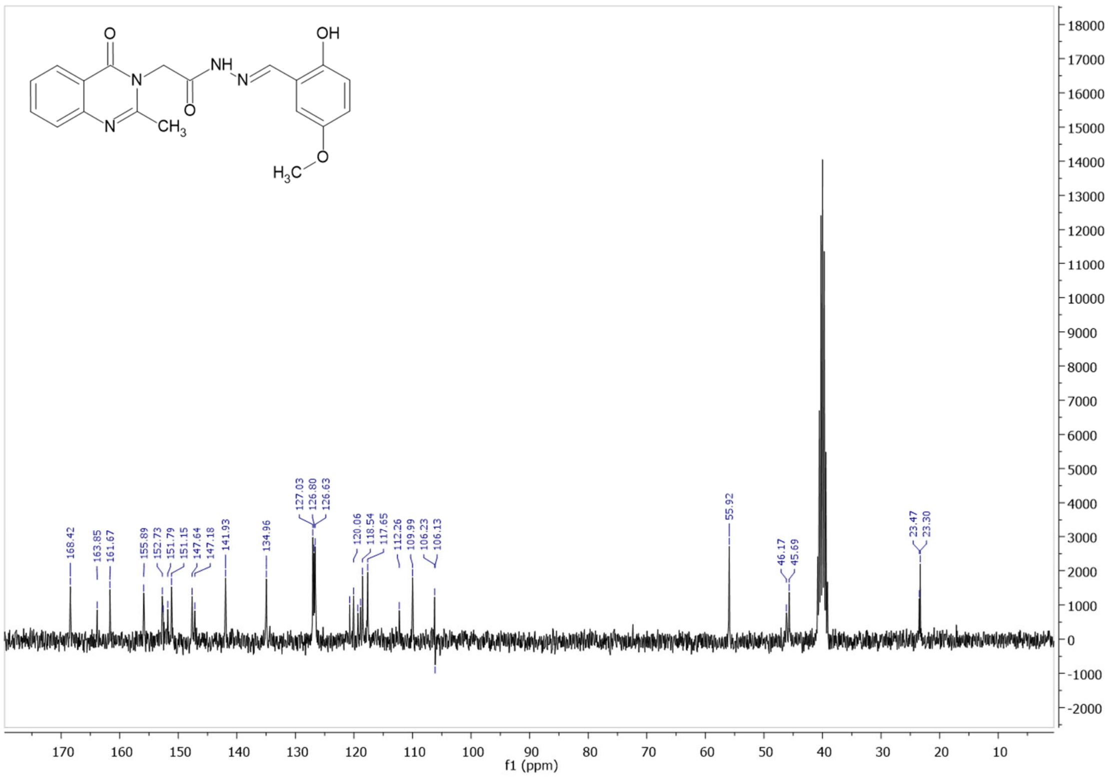

Figure S18. ^{13}C-RMN spectrum of N'-[(E)-(2-hydroxy-5-methoxyphenyl)methylidene]-2-(2-methyl-4-oxoquinazolin-3(4H)-yl)acetohydrazide (1e).

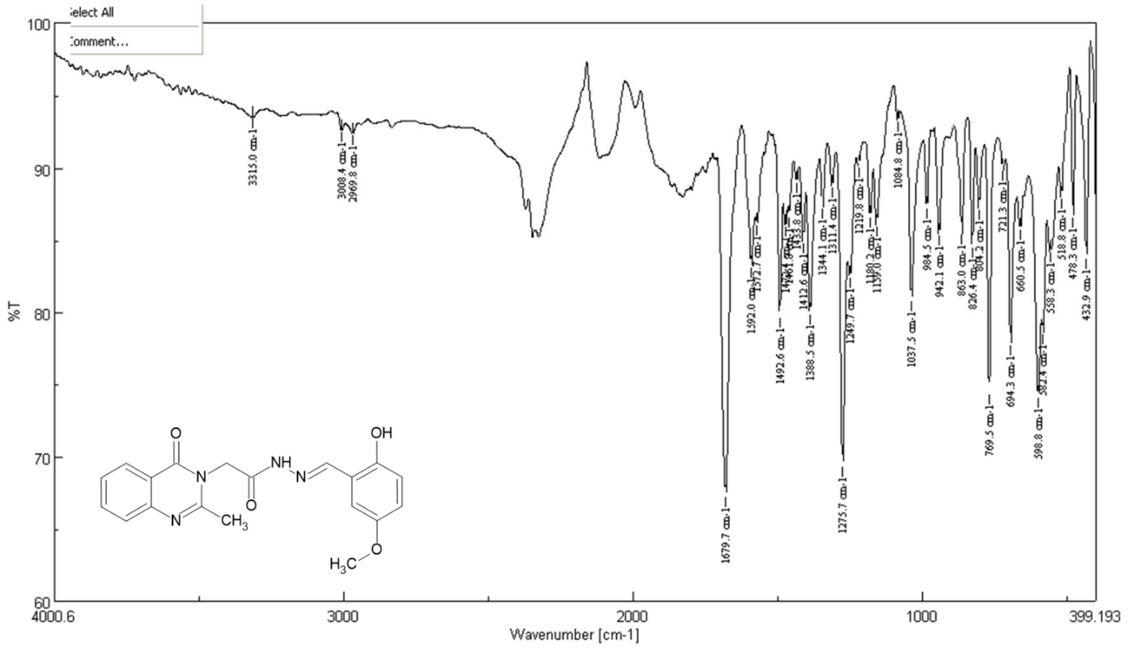

Figure S19. FT-IR spectrum of N'-[(E)-(2-hydroxy-5-methoxyphenyl)methylidene]-2-(2-methyl-4-oxoquinazolin-3(4H)-yl)acetohydrazide (1e).

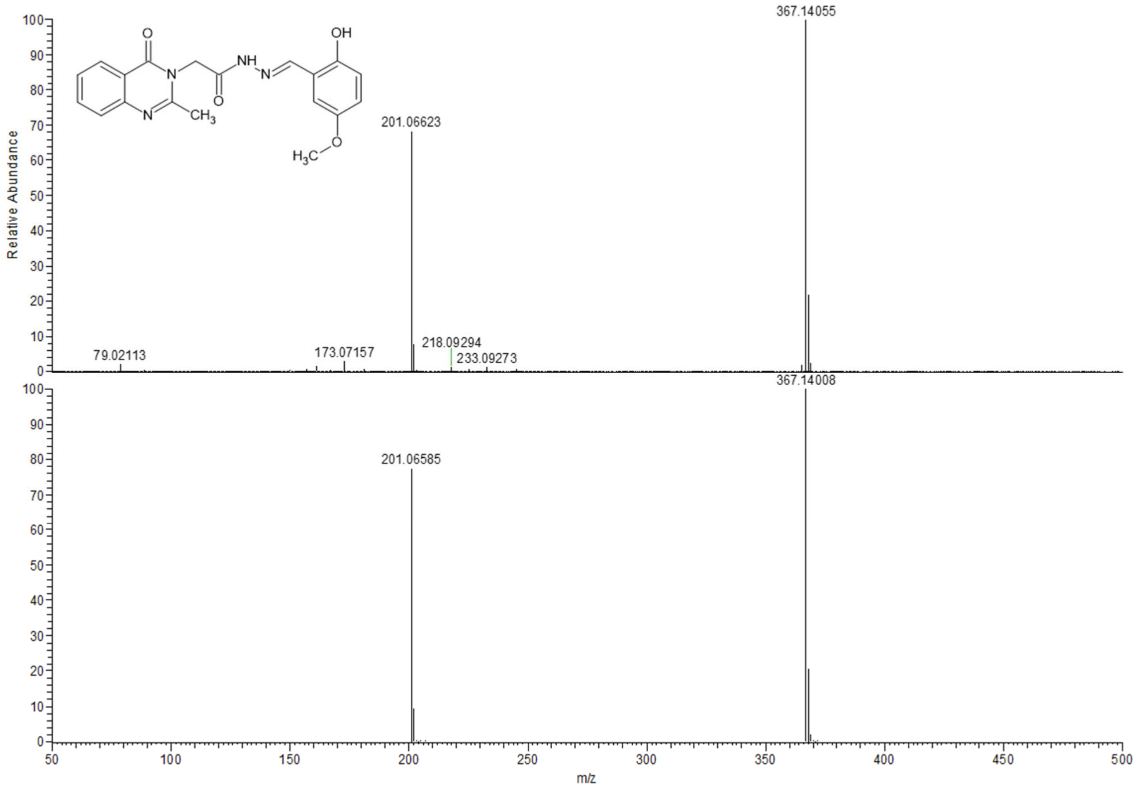

Figure S20. APCI+ MS spectrum of 1e in methanol. Experimental (up) and calculated (down) APCI+ MS spectra of 1e.

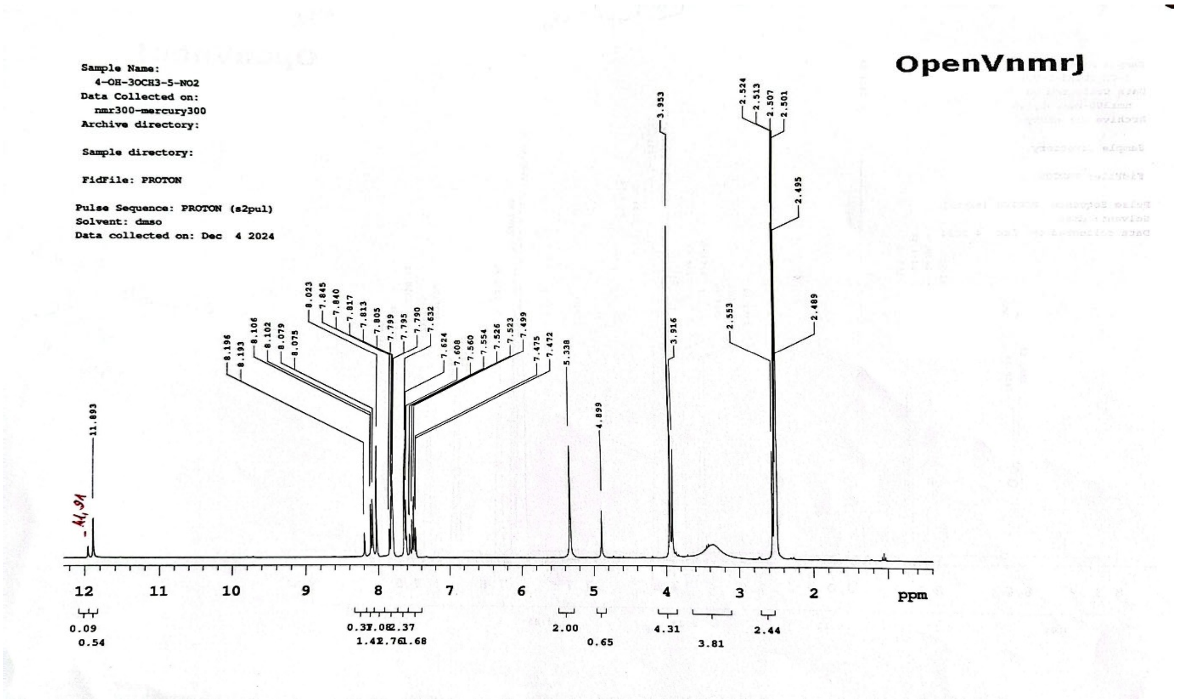

Figure S21. ^{1}H-RMN spectrum of N'-[(E)-(4-hydroxy-3-methoxy-5-nitrophenyl)methylidene]-2-(2-methyl-4-oxoquinazolin-3(4H)-yl)acetohydrazide (1f).

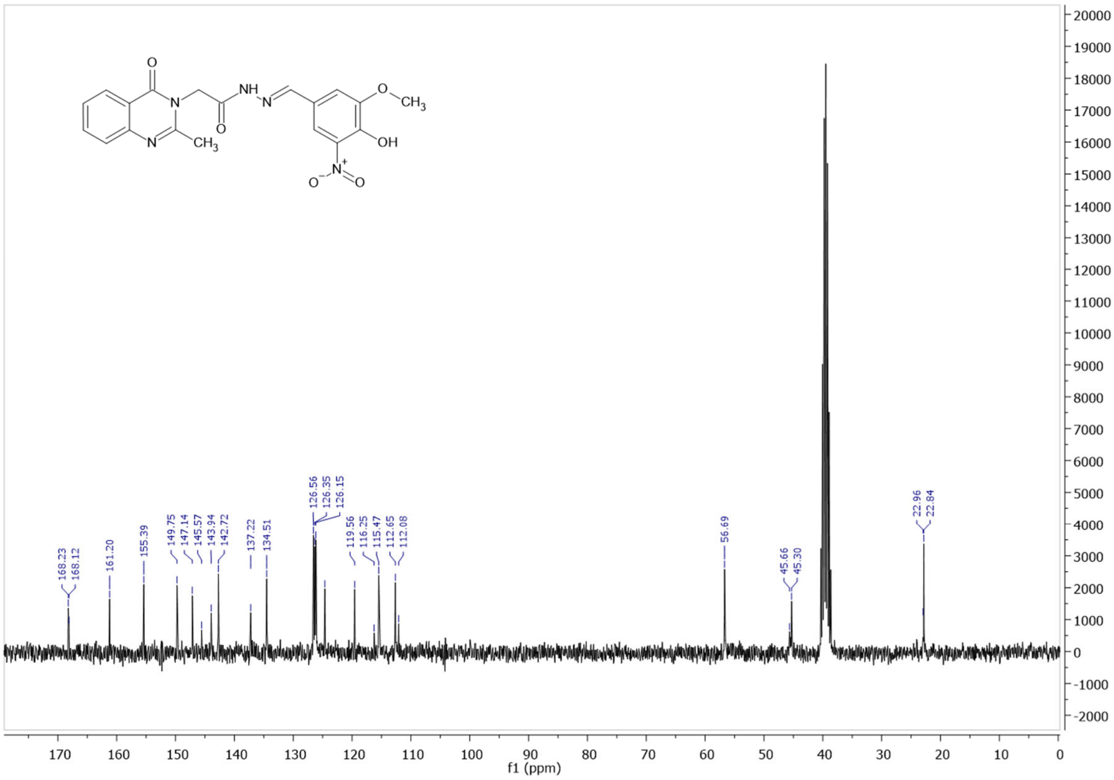

Figure S22. ^{13}C-RMN spectrum of N'-[(E)-(4-hydroxy-3-methoxy-5-nitrophenyl)methylidene]-2-(2-methyl-4-oxoquinazolin-3(4H)-yl)acetohydrazide (1f).

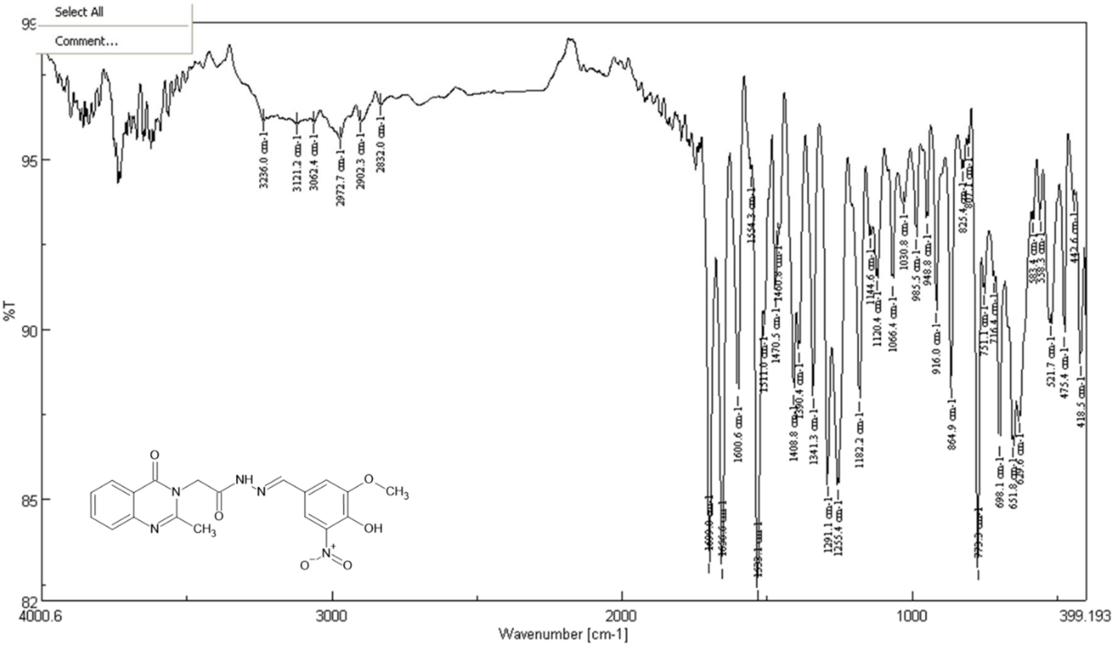

Figure S23. FT-IR spectrum of N'-[(E)-(4-hydroxy-3-methoxy-5-nitrophenyl)methylidene]-2-(2-methyl-4-oxoquinazolin-3(4H)-yl)acetohydrazide (1f).

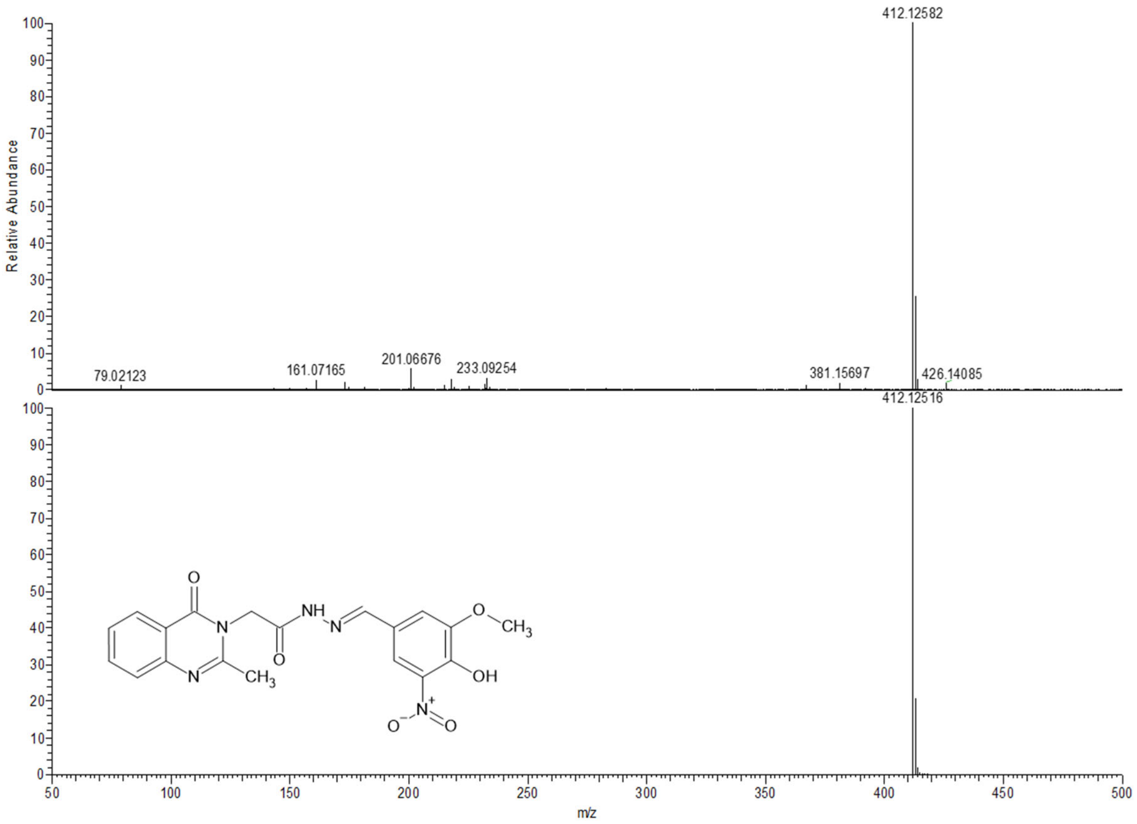

Figure S24. APCI+ MS spectrum of 1f in methanol. Experimental (up) and calculated (down) APCI+ MS spectra of 1f.

In silico pharmacodynamics

Table S1. Predicted druglikeness and leadlikeness properties of compounds 1a-1f (SwissADME).

Compound / Property	1a	1b	1c	1d	1e	1f
Molecular weight	415.24	494.14	366.37	366.37	366.37	411.37
#Rotatable bonds	5	5	6	6	6	7
#H-bond donors	2	2	2	2	2	2
#H-bond acceptors	5	5	6	6	6	8
Molar refractivity	102.74	110.44	101.53	101.53	101.53	110.36
TPSA	96.58	96.58	105.81	105.81	105.81	151.63
MLOG P	1.87	2.47	0.97	0.97	0.97	0.68
Lipinski #violations	0	0	0	0	0	1
Ghose #violations	0	1	0	0	0	0
Veber #violations	0	0	0	0	0	1
Egan #violations	0	0	0	0	0	1
Muegge #violations	0	0	0	0	0	1
Leadlikeness #violations	1	1	1	1	1	1

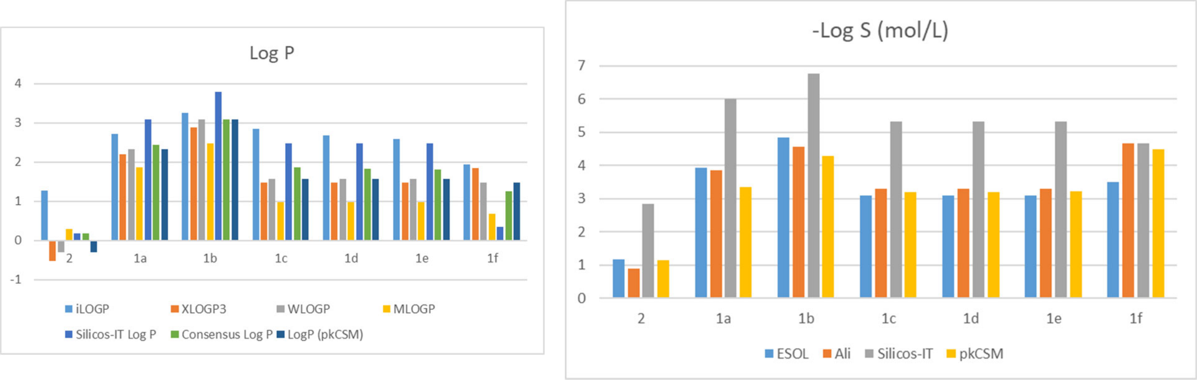

(**a**) (**b**)

Figure S25. LogP (a) and -LogS (b) predicted with different softwares (SwissADME, pkCSM) for compounds 1a-1f and acetohydrazide 2.

Table S2. Predicted absorption, distribution, metabolism, excretion and toxicity parameters for N-acyl-hydrazones 1a-1f.

Compound / Parameter	1a	1b	1c	1d	1e	1f
GI absorption *	high	high	high	high	high	low
BBB permeation *	no	no	no	no	no	no
P-gp substrate *	no	no	no	no	no	no
LogK_p – skin permeation (cm/s) *	-7.28	-7.27	-7.49	-7.49	-7.49	-7.5
CYP1A2 inhibitor *	no	yes	no	no	no	no
CYP2C19 inhibitor *	no	no	no	no	no	no
CYP2C9 inhibitor *	yes	yes	yes	yes	yes	no
CYP2D6 inhibitor *	no	no	no	no	no	no
CYP3A4 inhibitor *	yes	yes	no	no	no	no
Total Clearance (ml/min/kg) **	0.68	0.82	3.69	3.69	3.79	4.45
Oral Rat Acute Toxicity (LD_{50}) (mol/kg) **	2.05	2.03	2.01	2.44	2.18	2.61
Oral Rat Chronic Toxicity (LOAEL) (mg/kg_bw/day) **	196.7	370.4	597.9	753.7	769.4	925.6
hERG I **	no	no	no	no	no	no
hERG II **	no	yes	no	no	no	yes
AMES toxicity **	no	no	no	no	no	yes
Hepatotoxicity **	no	yes	yes	yes	yes	yes

* Predicted with SwissADME. ** Predicted with pkCSM.

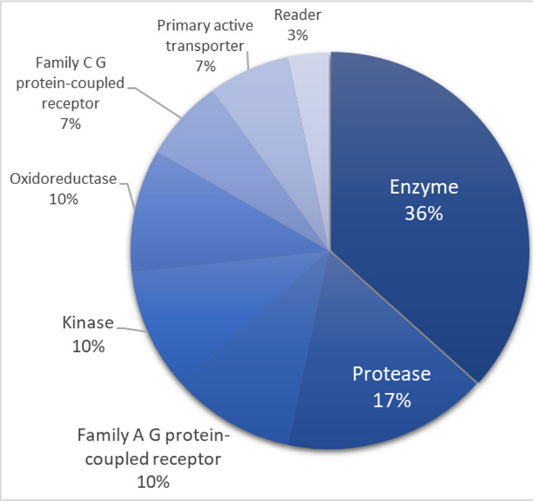

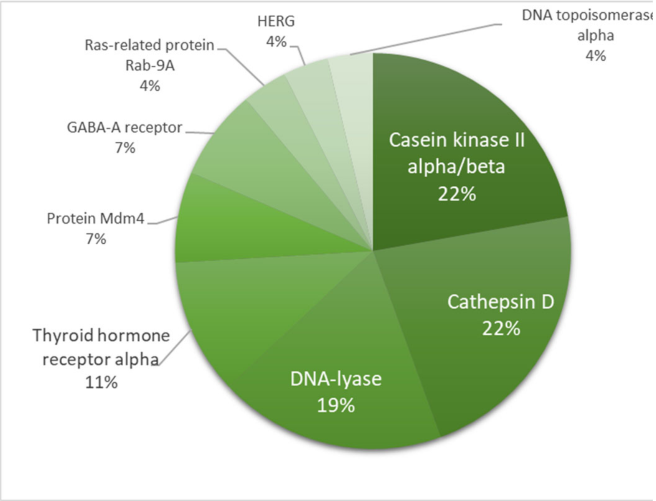

(**a**) (**b**)

Figure S26. Top target predictions for the series of N-acyl-hydrazones 1a-1f using SwissTargetPrediction (a) and SuperPred database (b).

Table S3. Top 5 protein targets for N-acyl-hydrazones 1a-1f (predicted with SwissTargetPrediction).

Compounds	Human molecular targets	Target class	Probability
1a	Matrix metalloproteinase 13	Protease	0,109339753
	Matrix metalloproteinase 1	Protease	0,109339753
	Hexokinase type IV	Enzyme	0,109339753
	P-glycoprotein 1	Primary active transporter	0,109339753
	Metabotropic glutamate receptor 4	Family C G protein-coupled receptor	0,109339753
1b	Telomerase reverse transcriptase	Enzyme	0,113285953
	Metabotropic glutamate receptor 4	Family C G protein-coupled receptor	0,113285953
	Adenosine A2a receptor	Family A G protein-coupled receptor	0,113285953
	Arachidonate 12-lipoxygenase	Enzyme	0,113285953
	DNA ligase 1	Enzyme	0,113285953
1c	LDL-associated phospholipase A2	Enzyme	0,113285953
	Multidrug resistance-associated protein 1	Primary active transporter	0,113285953
	Cyclooxygenase-2 (by homology)	Oxidoreductase	0,113285953
	Bromodomain-containing protein 4	Reader	0,113285953
	Kappa Opioid receptor	Family A G protein-coupled receptor	0,113285953
1d	Sphingosine kinase 2	Enzyme	0,113285953
	Sphingosine kinase 1	Enzyme	0,113285953
	Pyruvate dehydrogenase kinase isoform 1	Kinase	0,113285953
	Matrix metalloproteinase 1	Protease	0,113285953
	Matrix metalloproteinase 9	Protease	0,113285953
1e	Poly [ADP-ribose] polymerase-1	Enzyme	0,113285953
	Cystinyl aminopeptidase	Protease	0,113285953
	Hepatocyte growth factor receptor	Kinase	0,113285953
	Tyrosine-protein kinase receptor FLT3	Kinase	0,113285953
	Sphingosine kinase 2	Enzyme	0,113285953
1f	Alcohol dehydrogenase class III	Enzyme	0,106165761
	ATP-citrate synthase	Enzyme	0,106165761
	Egl nine homolog 1	Oxidoreductase	0,106165761
	4-hydroxyphenylpyruvate dioxygenase	Oxidoreductase	0,106165761
	Interleukin-8 receptor B	Family A G protein-coupled receptor	0,106165761

Table S4. Target prediction, indications, probability and model accuracy for N-acyl-hydrazones 1a-1f (predicted with SuperPRED).

Compounds	Human molecular targets	Target indications	Probability	Model Accuracy
1a	Casein kinase II alpha/beta	Cholangiocarcinoma	96%	99%
		Solid tumour/cancer		
	Thyroid hormone receptor alpha	Congestive heart failure	92%	99%
		High blood cholesterol level		
		Hypothyroidism		
		Wound healing		
	Cathepsin D	Hypertension	98%	99%
		Multiple sclerosis		
	DNA-(apurinic or apyrimidinic site) lyase	Glioma	98%	91%
		Melanoma		
		Ocular cancer		
		Solid tumour/cancer		
1b	Casein kinase II alpha/beta	Cholangiocarcinoma	94%	99%
		Solid tumour/cancer		
	Cathepsin D	Hypertension	98%	99%
		Multiple sclerosis		
	DNA-(apurinic or apyrimidinic site) lyase	Glioma	96,9%	91%
		Melanoma		
		Ocular cancer		
		Solid tumour/cancer		
1c	Casein kinase II alpha/beta	Cholangiocarcinoma	96%	99%
		Solid tumour/cancer		
	Cathepsin D	Hypertension	97%	99%
		Multiple sclerosis		
	DNA-(apurinic or apyrimidinic site) lyase	Glioma	96%	91%
		Melanoma		
		Ocular cancer		
		Solid tumour/cancer		
	Protein Mdm4	Acute myeloid leukaemia	93%	90,2%
		Haematological malignancy		
		Myelodysplastic syndrome		
		Solid tumour/cancer		
1d	Casein kinase II alpha/beta	Cholangiocarcinoma	94%	99%
		Solid tumour/cancer		
	Thyroid hormone receptor alpha	Congestive heart failure	94%	99%
		High blood cholesterol level		
		Hypothyroidism		
		Wound healing		
	Cathepsin D	Hypertension	97%	99%
		Multiple sclerosis		
	GABA-A receptor; alpha-1/beta-2/gamma-2	Alcohol dependence	93%	93%
		Anaesthesia		
		Anxiety disorder		
		Chronic pain		
		Cystitis		
		Depression		
		Epilepsy		

		Essential tremor or related tremors		
		Fibromyalgia		
		Headache		
		Inflammation		
		Insomnia		
		Ischemic reperfusion injury		
		Malaria		
		Muscle spasm		
		Respiratory distress syndrome		
	DNA-(apurinic or apyrimidinic site) lyase	Glioma	99%	91%
		Melanoma		
		Ocular cancer		
		Solid tumour/cancer		
	Ras-related protein Rab-9A	Fungal infection	93%	88%
1e	Casein kinase II alpha/beta	Cholangiocarcinoma	94%	99%
		Solid tumour/cancer		
	Thyroid hormone receptor alpha	Congestive heart failure	96%	99%
		High blood cholesterol level		
		Hypothyroidism		
		Wound healing		
	Cathepsin D	Hypertension	97%	99%
		Multiple sclerosis		
	GABA-A receptor; alpha-1/beta-2/gamma-2	Alcohol dependence	90%	93%
		Anaesthesia		
		Anxiety disorder		
		Chronic pain		
		Cystitis		
		Depression		
		Epilepsy		
		Essential tremor or related tremors		
		Fibromyalgia		
		Headache		
		Inflammation		
		Insomnia		
		Ischemic reperfusion injury		
		Malaria		
		Muscle spasm		
		Respiratory distress syndrome		
		Glioma		
		Melanoma		
		Ocular cancer		
		Solid tumour/cancer		
1f	Casein kinase II alpha/beta	Cholangiocarcinoma	94%	99%
		Solid tumour/cancer		
	Cathepsin D	Hypertension	94%	99%
		Multiple sclerosis		
	DNA-(apurinic or apyrimidinic site) lyase	Glioma	94%	91%
		Melanoma		
		Ocular cancer		
		Solid tumour/cancer		

	Protein Mdm4	Acute myeloid leukaemia	96,3%	90,2%
		Haematological malignancy		
		Myelodysplastic syndrome		
		Solid tumour/cancer		
	HERG	Angina pectoris	92,6%	90%
		Cardiac arrhythmias		
		Cardiac failure		
		Malaria		
		Multiple sclerosis		
		Ovarian cancer		
		Pain		
		Sleep-wake disorder		
	DNA topoisomerase II alpha	Solid tumour/cancer	91%	89%

Table S5. Predicted activity for N-acyl-hydrazones 1a-1f (Passonline simulations).

**Compounds**	**Activity**	**Pa**	**Pi**
1a	Antituberculosic	0,768	0,003
	Antiprotozoal (Amoeba)	0,334	0,048
	Antineoplastic (multiple myeloma)	0,291	0,088
	Anti-Helicobacter pylori	0,269	0,046
	Antineoplastic (brain cancer)	0,267	0,043
1b	Antituberculosic	0,728	0,004
	Antiprotozoal (Amoeba)	0,375	0,032
	Antiinfective	0,322	0,085
	Anti-Helicobacter pylori	0,279	0,039
	Antineoplastic (brain cancer)	0,22	0,094
1c	Antituberculosic	0,659	0,005
	Antineoplastic (multiple myeloma)	0,349	0,044
	Anti-Helicobacter pylori	0,32	0,022
	Antineoplastic (brain cancer)	0,278	0,036
	Antiprotozoal (Amoeba)	0,27	0,098
1d	Antituberculosic	0,661	0,005
	Antineoplastic (multiple myeloma)	0,326	0,058
	Anti-Helicobacter pylori	0,318	0,022
	Antineoplastic (brain cancer)	0,27	0,041
	Antineoplastic (bone cancer)	0,227	0,074
1e	Antituberculosic	0,689	0,005
	Anti-Helicobacter pylori	0,681	0,004
	Antineoplastic (multiple myeloma)	0,317	0,022
	Antineoplastic (brain cancer)	0,313	0,068
	Antineoplastic (bone cancer)	0,222	0,087
1f	Antituberculosic	0,723	0,004
	Antiprotozoal (Coccidial)	0,389	0,022
	Antiprotozoal (Trichomonas)	0,26	0,056
	Antineoplastic (brain cancer)	0,248	0,059
	Antineoplastic (bone cancer)	0,235	0,057

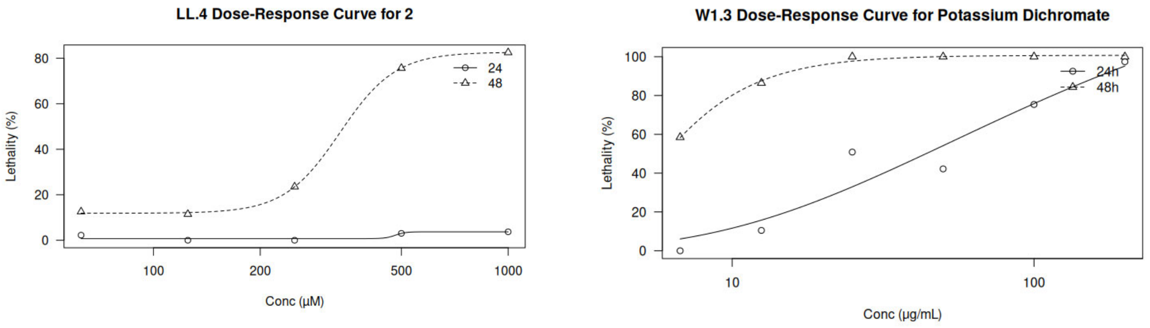

(a) (b)

Figure S27. Dose-response curves for compound 2 (2-(2-methyl-4-oxoquinazolin-3(4H)-yl)acetohydrazide) (a) and potassium dichromate (b) in the lethality test on Artemia franciscana. Lethality is expressed as the mean lethality %.

Table S6. Docking scores, molecule contributions, binding interactions of selected ligands in the active site of 4URO protein.

Ligand	MolDock Score	Molecule Contributions	Bond type	Bond Length (Å)
**Co- crystallized Novobiocin**	-125.51	ALA 98, AR84, ARG 144, ASN 54, ASP 57, ASP 81, ASP 89, GLN 91, GLU 58, GLY 85, GLY 125, ILE 86, ILE 102, ILE 175, PRO 87, SER 55, SER 128, THR 173	**Hydrogen bonds**	
			O sp^3 (O3) – O sp^3 from ASP 89	2.631
			O sp^3 (O3) – N sp^2 from GLN 91	3.421
			O sp^2 (O11) – N sp^2 from ARG 144	2.912
			O sp^2 (O11) – N sp^2 from ARG 144	2.659
			O sp^3 (O6) – O sp^2 from ASN 54	2.641
			N sp^2 (N1) – O sp^3 from SER 55	3.468
			N sp^2 (N1) – O sp^2 from ASP 81	2.788
			**Steric interactions**	
			C sp^2 (C18) - O sp^3 from ASP 89	3.062
**1b**	-91.57	ALA 61, AR84, ASN 54, ASP 57, ASP 81, GLU 58, GLY 83, GLY 85, ILE 51, ILE 86, ILE 175, LEU 60, SER 55, SER 128, THR 173	**Hydrogen bonds**	
			O sp^3 (O1) – N sp^2 from ARG 84	3.014
			N sp^2 (N1) - O sp^2 from GLU 58	3.088
			N sp^2 (N) - O sp^3 from THR 173	3.242
			**Steric interactions**	
			C sp^2 (C1) - C sp^3 from ASN 54	2.940
			C sp^2 (C18) - C sp^3 from THR 173	3.171
			C sp^2 (C18) - O sp^2 from ASP 81	2.672

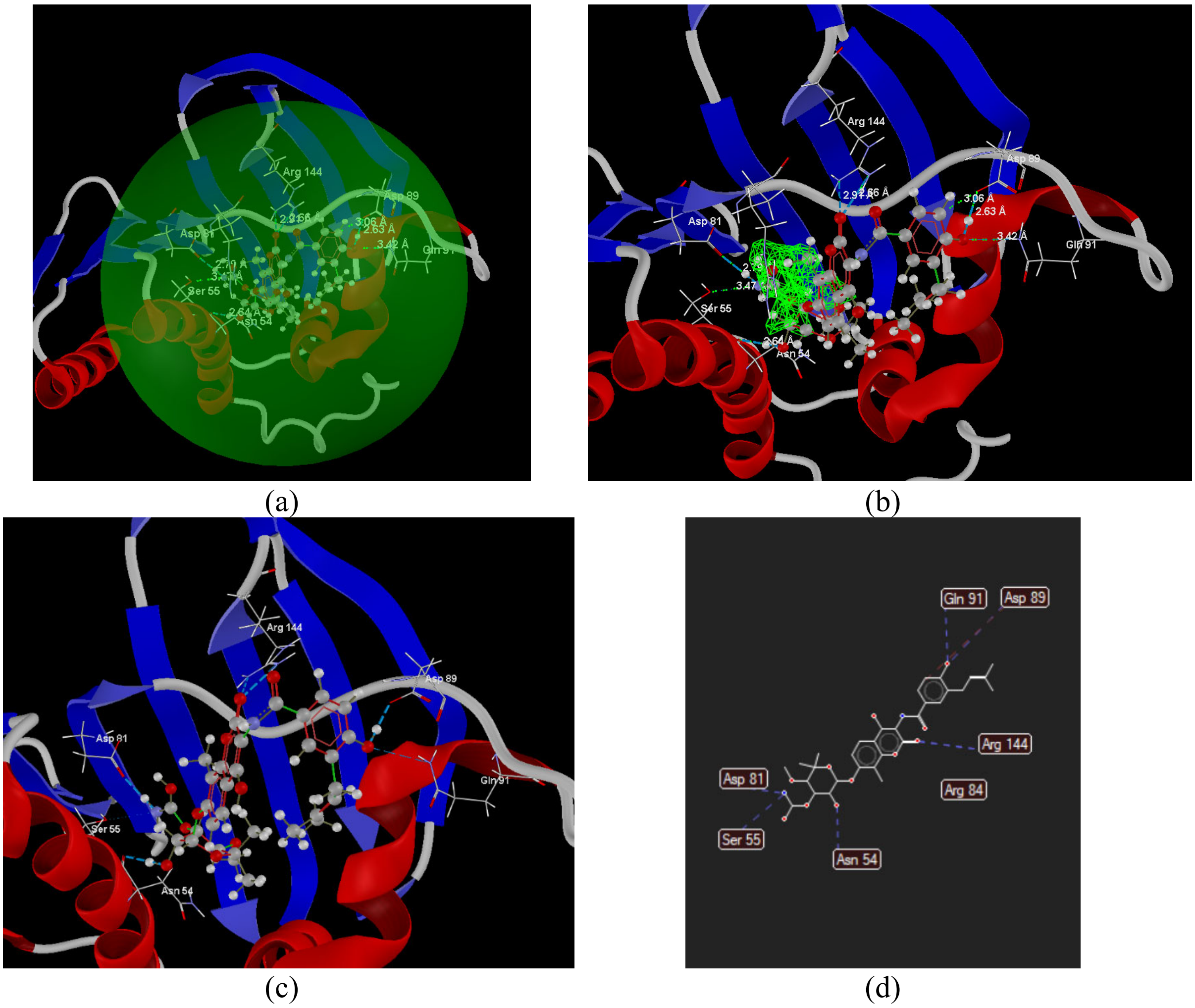

Figure S28. 4URO protein: Binding site (a) and binding pocket (b) of the co-crystallized novobiocin. Hydrogen bond (blue dotted lines) and steric interactions (red dotted) between novobiocin and amino acid residues from the binding site of 4URO (c – 3D structure, d – 2D structure).

Table S7. Docking scores, molecule contributions, binding interactions of selected ligands in the active site of 2W9H protein.

Ligand	MolDock Score	Molecule Contributions	Bond type	Bond Length (Å)
**Co- crystallized Trimethoprim**	-95.26	ALA 7, ASN 18, ASP 27, GLN 19, GLY 93, ILE 14, ILE 50, LEU 5, LEU 20, LEU 28, LEU 54, PHE 92, PHE 98, SER 48, THR 46, THR 111, VAL6, VAL 31.	**Hydrogen bonds**<br>O sp^3 (O13) – O sp^3 from SER 49<br>N sp^2 (N7) – O sp^2 from PHE 92<br>N sp^2 (N7) – O sp^2 from LEU 5<br>N sp^2 (N5) – N sp^2 from ALA 7<br>N sp^2 (N4) – O sp^3 from ASP 27<br>**Steric interactions**<br>N sp^2 (N2) – O sp^2 from ASP 27	 3.423<br>2.879<br>2.835<br>3.498<br>2.850<br><br>2.713
**1b**	-113.49	ARG 44, ASN 18, GLN 19, GLN 95, GLY 15, GLY 43, GLY 93, GLY 94, ILE 14, ILE 50, LEU 5, LEU 20, LEU 28, LYS 45, PHE 92, SER 49, THR 46, THR 96, THR 121.	**Hydrogen bonds**<br>O sp^3 (O1) – N sp^2 from GLY 94<br>O sp^3 (O1) – N sp^2 from THR 46<br>O sp^3 (O1) – O sp^3 from THR 46<br>N sp^2 (N1) - O sp^3 from THR 46<br>O sp^2 (CO) - O sp^3 from SER 49	 3.174<br>3.041<br>2.775<br>2.821<br>2.736

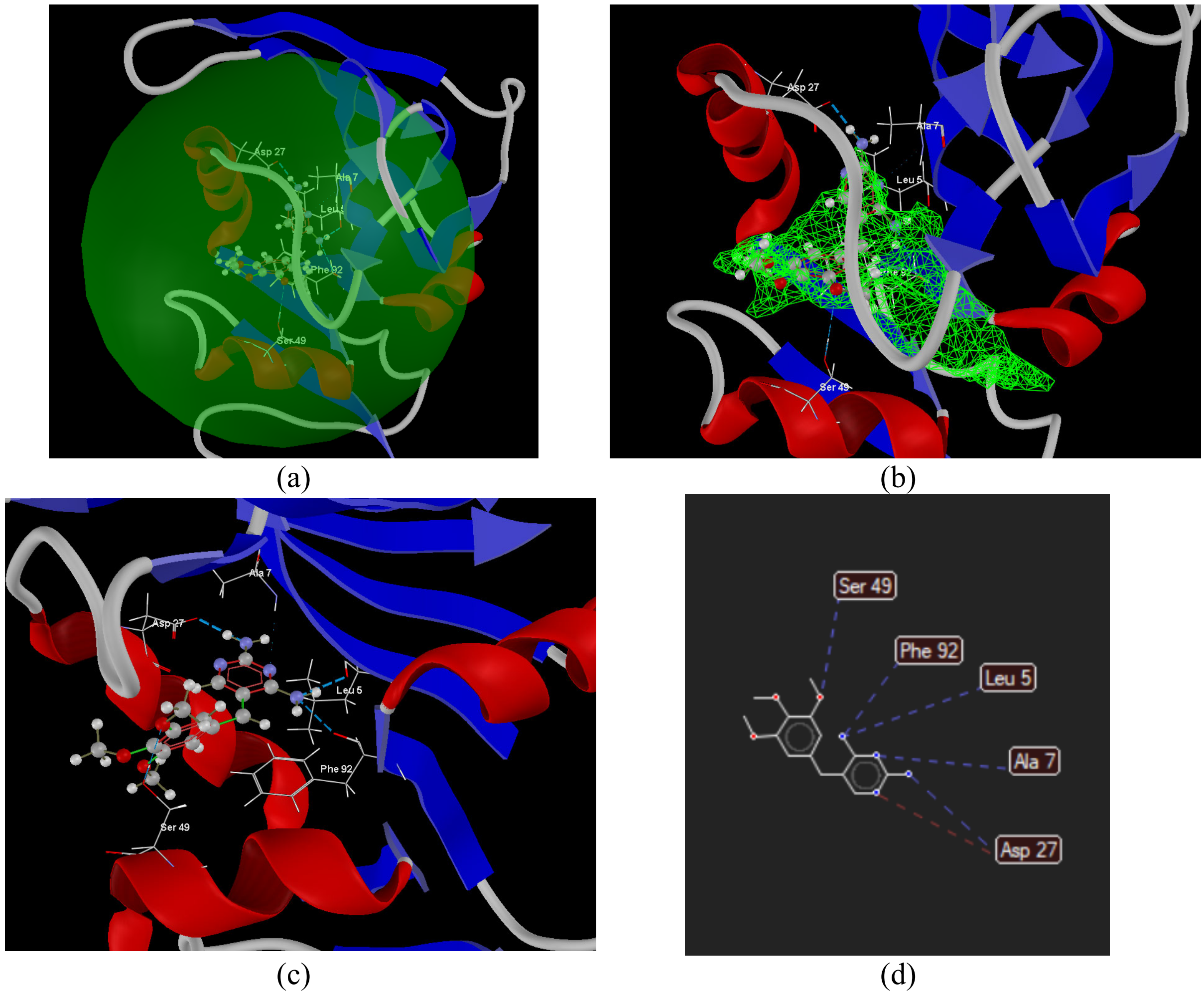

Figure S29. 2W9H protein: Binding site (a) and binding pocket (b) of the co-crystallized trimethoprim. Hydrogen bond (blue dotted lines) and steric interactions (red dotted) between trimethoprim and amino acid residues from the binding site of 2W9H (c – 3D structure, d – 2D structure).

Table S8. Docking scores, molecule contributions, and binding interactions of selected ligands in the active site of 5FSA protein.

Ligand	MolDock Score	Molecule Contributions	Bond type	Bond Length (Å)
**Co-crystallized Posoconazole**	-175.10	ALA 61, ALA 62, CYS 470, GLN 66, GLY 65, GLY 303, GLY 307, GY 308, HIS 377, ILE 131, ILE 304, LEU 87, LEU 88, LEU 121, LEU 376, MET 508, PHE 58, PHE 126, PHE 228, PHE 233, PHE 380, SER 378, SER 506, SER 507, THR 122, THR 311, TYR 64, TYR 118, TYR 132, TYR 505, TYR 509.	**Steric interactions**<br>C sp^2 (CAL) - O sp^2 from SER 506<br>C sp^2 (CAN) - O sp^2 from SER 506<br>C sp^3 (CAZ) - O sp^2 from SER 507<br>C sp^3 (CAX) - O sp^2 from SER 507<br>C sp^2 (CAJ) - C sp^2 from PHE 233<br>C sp^2 (CAK) - O sp^2 from MET 508<br>C sp^3 (CBB) - C sp^3 from LEU 121<br>N sp^2 (NBD) - O sp^2 from GLY 307	 3.006<br>2.849<br>3.089<br>3.115<br>3.083<br>3.154<br>3.107<br>3.110
**Ketoconazole**	-178.94	GLY 65, GLY 303, GLY 307, GY 308, HIS 377, ILE 304, LEU 87, LEU 88, LEU 121, LEU 300, LEU 376, MET 508, PHE 126, PHE 228, PHE 233, PHE 380, PRO 230, SER 378,	**Hydrogen bonds**<br>O sp^3 (O3) – O sp^3 from TYR 132<br>**Steric interactions**<br>Cl sp^3 (Cl 3) - C sp^3 from ILE 131	 3.080<br><br>3.029

		SER 506, SER 507, THR 122, THR 311, TYR 64, TYR 118, TYR 132, TYR 505.		
**1b**	-111.45	GLY 65, HIS 377, ILE 379, LEU 87, LEU 88, LEU 121, LEU 376, MET 508, PHE 58, PHE 126, PHE 228, PHE 233, PHE 380, PRO 230, SER 378, SER 506, SER 507, THR 122, TYR 64, TYR 118, TYR 132.	**Hydrogen bonds**<br>O sp^2 (CO) – O sp^3 from TYR 132<br>O sp^2 (CO-N) – O sp^3 from TYR 118<br>**Steric interactions**<br>C sp^2 (C2) – C sp^3 from GLY 307<br>C sp^2 (C7) – S sp^3 from MET 508	 3.049<br>3.036<br><br>3.160<br>3.014

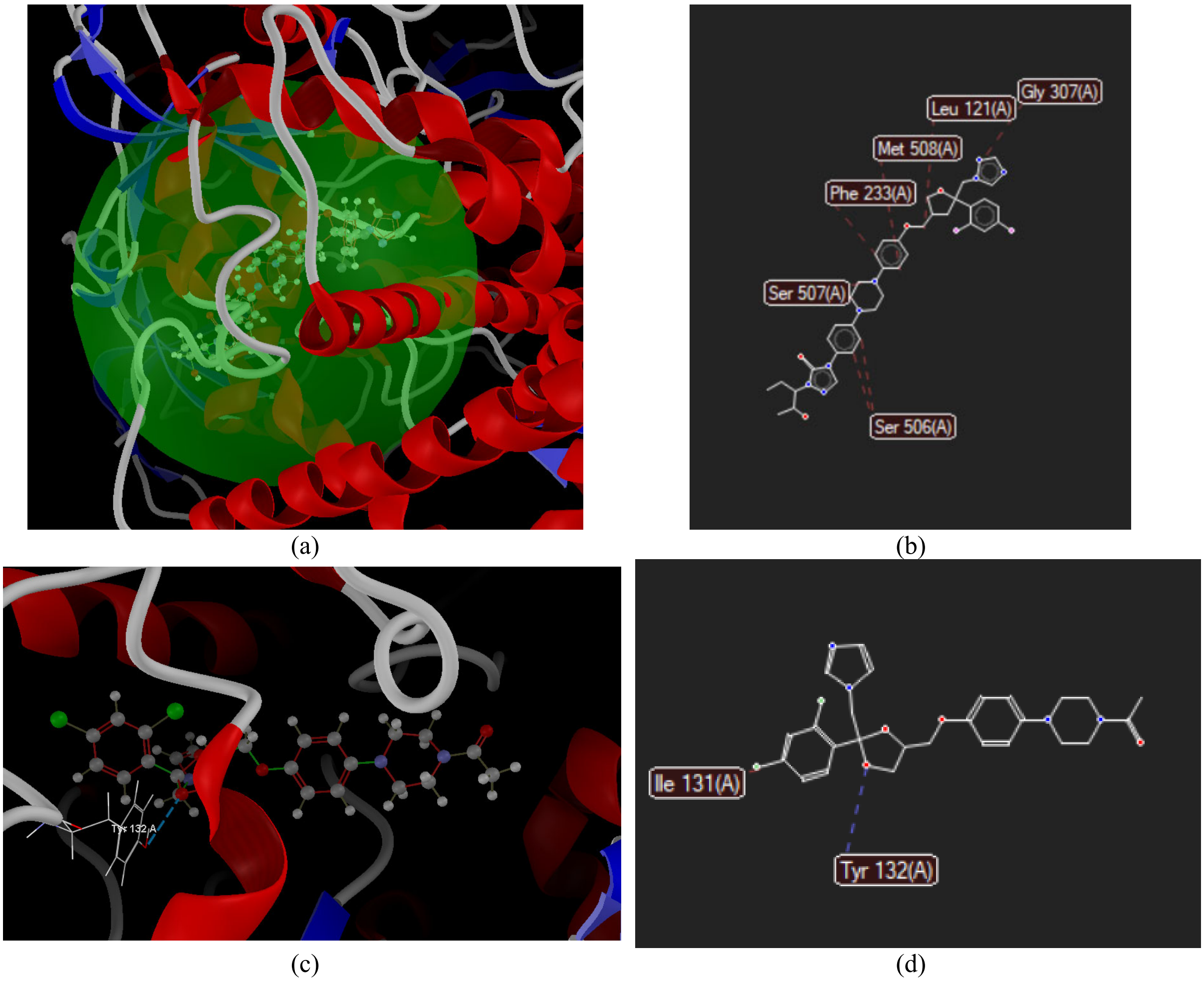

Figure S30. (a) Binding site of the co-crystallized posaconazole inside the 5FSA protein. (b) Steric interactions (red dotted) between co-crystallized posaconazole and amino acid residues from the binding site of 5FSA receptor (2D). Hydrogen bond (blue dotted lines) between ketoconazole and amino acid residues from the binding site of 5FSA receptor (c - 3D structure, d – 2D structure).

Table S9. Docking scores, molecule contributions, and binding interactions of selected ligands in the active site of 1XKK protein.

Ligand	MolDock Score	Molecule Contributions	Bond type	Bond Length (Å)

**Co-crystallized Lapatinib**	-177.84	ALA 743, ARG 776, ARG 803, ARG 841, ASP 800, ASP 855, CYS 775, CYS 797, GLN 791, GLY 719, GLY 796, ILE 744, ILE 789, LEU 718, LEU 777, LEU 788, LEU 792, LEU 799, LEU 844, LEU 858, LYS 745, MET 766, MET 793, MET 1002, PHE 856, THR 790, THR 854, VAL 726.	**Hydrogen bonds**<br>N sp^2 (N18) - N sp^2 from MET 793<br>O sp^2 (O3) - O sp^3 from PO4_81<br>**Steric interactions**<br>F sp^3 (F34) - C sp^2 from ARG 776<br>F sp^3 (F34) - O sp^2 from ARG 776<br>F sp^3 (F34) - O sp^3 from THR 790<br>Cl sp^3 (Cl3) - O sp^3 from LEU 788<br>C sp^2 (C19) - O sp^2 from GLN 791<br>C sp^3 (C1) - O sp^2 from ASP 800<br>O sp^2 (O4) - O sp^3 from ASP 800<br>C sp^3 (C1) - S sp^3 from CYS 797	 2.960<br>2.908<br><br>3.123<br>3.186<br>3.138<br>3.100<br>3.195<br>3.057<br>3.129<br>3.129
**1a**	-130.68	ALA 743, ARG 776, ASP 855, CYS 775, CYS 797, GLN 791, GLY 796, ILE 744, ILE 789, LEU 718, LEU 777, LEU 788, LEU 792, LEU 844, LEU 858, LYS 745, MET 766, MET 793, MET 1002, PHE 856, THR 790, THR 854, VAL 726.	**Hydrogen bonds**<br>O sp^2 (CON1) - O sp^3 from LYS 745<br>O sp^2 (CO) - O sp^2 from GLN 791<br>**Steric interactions**<br>C sp^2 (C8) - N sp^2 from MET 793<br>C sp^2 (C5) - O sp^2 from ALA 743<br>C sp^3 (C4) - C sp^3 from LYS 745<br>C sp^3 (C10) - C sp^3 from THR 790<br>O sp^2 (CO) - C sp^3 from ASP 855<br>O sp^2 (CO) - O sp^3 from ASP 855	 3.145<br>2.938<br><br>3.183<br>3.172<br>2.705<br>3.071<br>3.134<br>2.610
**1b**	-119.19	ALA 743, ARG 776, ARG 841, ASN 842, ASP 855, CYS 775, CYS 797, GLY 796, ILE 744, ILE 789, LEU 718, LEU 777, LEU 788, LEU 844, LEU 858, LYS 745, MET 766, MET 793, MET 1002, PHE 856, THR 790, THR 854, VAL 726.	**Hydrogen bonds**<br>O sp^2 (O1) – O sp^2 from ASP 855<br>O sp2 (O1) – N sp3 from LYS 745<br>O sp2 (CO) - O sp3 from THR 790<br>**Steric interactions**<br>O sp^2 (CON1) - C sp^3 from THR 790<br>O sp2 (CO) - C sp3 from THR 790<br>O sp2 (CO) - C sp3 from THR 790<br>C sp3 (C9) - C sp3 from LYS 745	 2.729<br>3.356<br>3.186<br><br>3.167<br>2.622<br>2.847<br>2.427
**1c**	-85.58	ALA 743, ARG 841, ASP 800, ASP 855, CYS 797, GLY 796, ILE 744, ILE 789, LEU 718, LEU 777, LEU 788, LEU 844, LEU 858, LEU 1001, LYS 745, MET 766, MET 793, MET 1002, PHE 795, PHE 790, THR 854, VAL 726.	**Hydrogen bonds**<br>O sp^2 (O1) – N sp^2 from ASP 855<br>O sp^2 (O1) – O sp^3 from THR 790<br>**Steric interactions**<br>C sp^3 (C11) - C sp^3 from MET 766	 3.281<br>2.925<br><br>2.738
**1d**	-92.73	ALA 743, ARG 841, ASN 842, ASP 800, ASP 855, CYS 797, GLY 719, GLY 721, ILE 744, ILE 789, LEU 718, LEU 777, LEU 788, LEU 844, LEU 858, LYS 745, MET 766, SER 720, THR 790, THR 854, VAL 726.	**Hydrogen bonds**<br>O sp^2 (CON1) - N sp^3 from LYS 745<br>O sp2 (CO) - N sp2 from ASP 855<br>O sp2 (CO) - O sp3 from THR 854<br>**Steric interactions**<br>O sp^2 (CON1) - O sp^2 from ASP 855<br>O sp^2 (CO) - C sp^2 from ASP 855<br>O sp^2 (CO) - O sp^3 from ASP 855	 3.096<br>3.345<br>2.962<br><br>3.032<br>3.028<br>3.083
**1e**	-128.01	ALA 743, ARG 776, ASP 855, CYS 775, CYS 797, GLN 791, GLY 796, ILE 744, ILE 789, LEU 718, LEU 777, LEU 788, LEU 792, LEU 844, LEU 858, LYS 745, MET 766, MET 793, MET 1002, PHE 856, THR 790, THR 854, VAL 726.	**Hydrogen bonds**<br>O sp^2 (CO) - N sp^2 from ASP 855<br>O sp^3 (O1) – O sp^2 from GLN 791<br>**Steric interactions**<br>O sp^2 (CO) - O sp^3 from ASP 855<br>C sp2 (C9) - N sp2 from ASP 855<br>N sp2 (N) - C sp3 from LEU 777<br>C sp^3 (C10 - C sp^3 from THR 790	 3.093<br>3.232<br><br>2.988<br>2.953<br>2.709<br>3.163

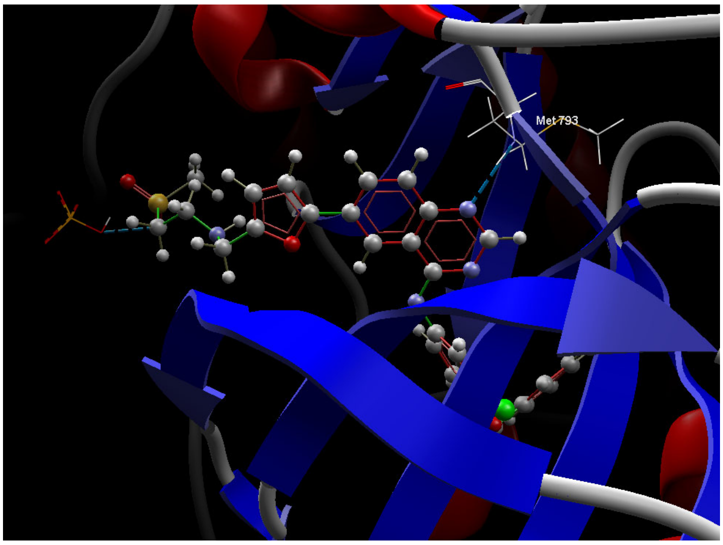
Met 793

(a)

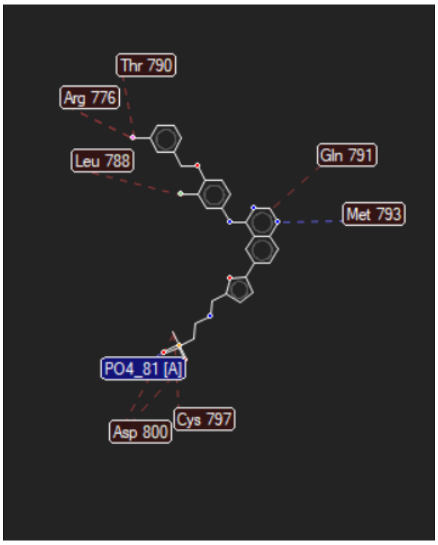
Thr 790
Arg 776
Leu 788
Gln 791
Met 793
PO4_81 [A]
Cys 797
Asp 800

(b)

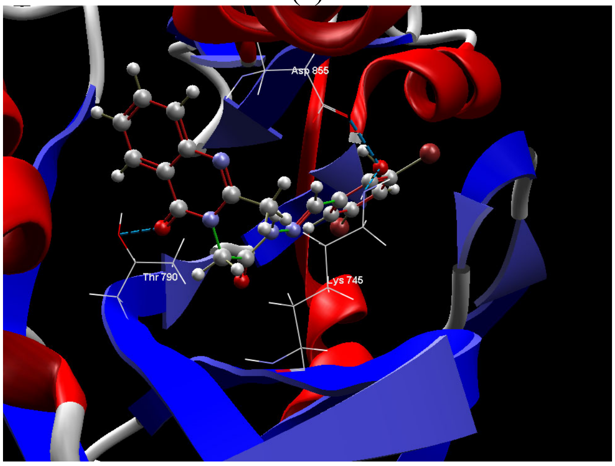
Asp 855
Thr 790
Lys 745

(c)

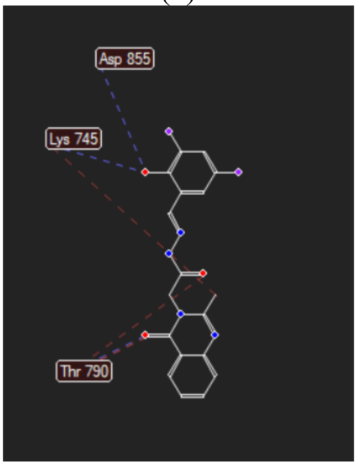
Asp 855
Lys 745
Thr 790

(d)

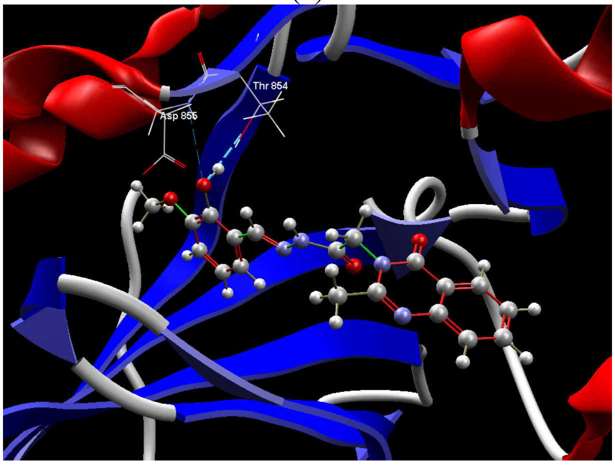
Thr 854
Asp 855

(e)

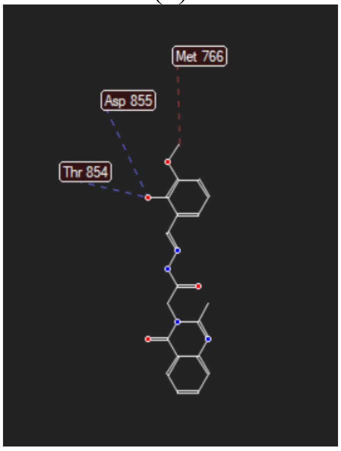
Met 766
Asp 855
Thr 854

(f)

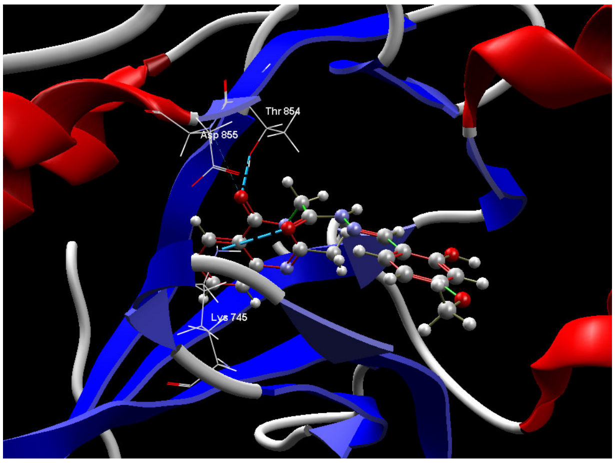

(g)

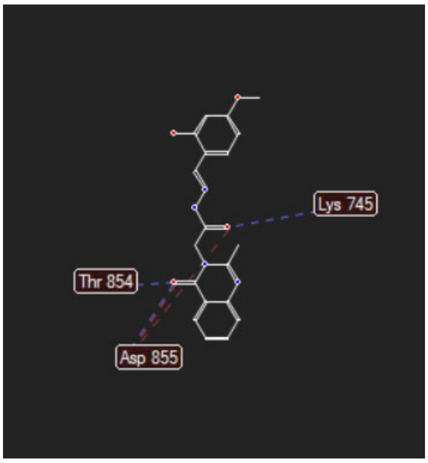

(h)

Figure S31. Hydrogen bond (blue dotted lines) and steric interactions (red dotted) selected ligands and the amino acid residues from the binding site of 1XKK protein: lapatinib (a – 3D structure, b - 2D structure), compound 1b (c – 3D structure, d - 2D structure), compound 1c (e – 3D structure, f - 2D structure), compound 1d (g – 3D structure, h - 2D structure).
